# Comparison of efficacy and safety of thrombus prevention strategies after abdominal and pelvic cancer surgery: Bayesian network based meta-analysis

**DOI:** 10.3389/fonc.2025.1445485

**Published:** 2025-02-11

**Authors:** Shiran Qin, Sitong Guo, Yucheng Yao, Ying He, Dandan Xu, Henghai Su, Xiaoyu Chen, Haoru Meng

**Affiliations:** ^1^ Department of Pharmacy, Guangxi Academy of Medical Sciences and the People’s Hospital of Guangxi Zhuang Autonomous Region, Nanning, China; ^2^ School of Pharmacy, Guangxi Medical University, Nanning, China; ^3^ School of Pharmacy, Guilin Medical College, Guilin, China

**Keywords:** cancer, surgery, venous thromboembolism, DOAC, meta-analysis

## Abstract

**Background:**

The occurrence of venous thromboembolism (VTE) after abdominal and pelvic cancer surgery increases the risk of mortality and disability. However, there is insufficient evidence supporting the choice of anticoagulation strategies.

**Methods:**

We searched PubMed, The Cochrane Library, Embase, and Web of Science for randomized controlled trials from inception to January 2024. Studies concerning thrombosis prevention after abdominal and pelvic surgery were included. Network meta-analysis(NMA) and direct meta-analysis (DMA) methods were employed to evaluate the efficacy and safety of various prophylactic strategies.

**Results:**

Twenty clinical trials involving a total of 4923 patients were included. The DMA results showed that low molecular weight heparin (LMWH) was more effective in preventing VTE compared to no treatment (OR = 1.96; 95% CI: 1.21 to 3.19), and LMWH plus physiotherapy was more effective than LMWH (OR = 10.95; 95% CI: 1.33 to 90.40). The NMA results indicated that DOACs (OR = 0.34; 95% CI: 0.11 to 0.76) and LMWH (OR = 0.51; 95% CI: 0.32 to 0.77) were significantly effective in preventing venous thrombosis compared with no treatment. The cumulative ranking probability curve (SUCRA) showed that direct oral anticoagulants (DOACs) were the best intervention. In terms of major bleeding, unfractionated heparin (UFH) had a higher risk than LMWH, physiotherapy, and no treatment, with statistically significant differences. The SUCRA analysis indicated that physiotherapy was the best intervention for major bleeding.

**Conclusion:**

Existing evidence suggests that DOACs can provide better thromboprophylaxis efficacy for patients after abdominal and pelvic cancer surgery, achieving an optimal balance between efficacy and safety. LMWH has become an intervention with efficacy second only to DOACs, with similar safety.

**Systematic Review Registration:**

https://www.crd.york.ac.uk/prospero/ **, identifier CRD42024513090**.

## 1 Introduction

Tumor-associated venous thromboembolism (TAVTE) refers to the occurrence of venous thromboembolism (VTE) in cancer patients. VTE includes pulmonary embolism (PE) and deep vein thrombosis (DVT), with an incidence rate of 4% to 20%. It is a major cause of death, second only to cancer itself. According to studies, the rate of venous thromboembolis in cancer patients population is about 4 to 7 times higher than that of the general population, and it is increasing annually (1–3).

The incidence of VTE varies depending on the type and site of cancer, and trauma surgery also increasing the risk of VTE. High-risk cancer sites for VTE include the brain, pancreas, stomach, bladder, gynecological organs, lungs, lymphoma, and kidneys (4–6). Risk factors for pathological VTE include tumor compression, alterations in hemodynamics caused by surgery, and the expression of tumor factors that increase coagulation components. It can be concluded that patients undergoing major abdominal and pelvic cancer surgery face a particularly high of VTE and these patients often also have risk factors such as advanced age, obesity, and prolonged periods of inactivity. The occurrence of VTE increases the risk of death for cancer patients by 2-6 times and can also lead to long-term disability (7). Therefore, preventing VTE in patients undergoing abdominal and pelvic cancer surgery is a crucial clinical concern.

Currently, the preventive strategies for VTE include pharmacological and physical interventions. Pharmacological prophylaxis options mainly include low molecular weight heparin (LMWH), unfractionated heparin (UFH), fondaparinux, and direct oral anticoagulants (DOACs). Physical prevention methods options primarily consist of the use of graduated compression stockings (GCS) and intermittent pneumatic compression (IPC), among others. The guidelines from the Chinese Society of Clinical Oncology (CSCO) (2), American Society of Hematology (ASH) (8), and the National Comprehensive Cancer Network (NCCN) (9) and other related guideline (10) recommend a 4-week period of anticoagulant thromboprophylaxis for high-risk surgical oncology patients. Suggest using a combination of mechanical and pharmacological prophylaxis for VTE high-risk patients with low risk of major bleeding, instead of using physiotherapy alone. Recommended pharmacological options for prevention include LMWH, UFH, or fondaparinux (2, 8–10). NCCN and the American Society of Clinical Oncology (ASCO) also recommend the use of apixaban and rivaroxaban as options for extended thromboprophylaxis after cancer surgery (10, 11). However, the strength of these recommendations is weaker due to limited evidence, with only three high-quality randomized controlled studies providing the basis (12–14). Moreover, the data for apixaban is specifically limited to gynecological cancer patients. Therefore, in light of these limitations, we conducted direct and network meta-analysis to compare the effectiveness and safety of each prophylactic strategy following abdominal pelvic cancer surgery, with the aim of providing more robust evidence for post-operative prevention options in this patient population.

## 2 Materials and methods

The current study follows the Preferred Reporting Items for Systematic Reviews and Meta-Analyses of Network Meta-Analyses (PRISMA-NMA) framework (15). The complete protocol was registered in PROSPERO with registration number CRD42024513090. Since it does not involve personal information of patients, ethical approval is not required for this study.

### 2.1 Searching strategies and eligibility criteria

We searched PubMed, The Cochrane Library, Embase, and Web of Science for randomized controlled trials (RCTs) on postoperative anticoagulation in abdominal and pelvic tumors from inception until January 12, 2024. The search was carried out by combining subject headings with free words, without limiting language. Additionally, we supplemented the search terms by reviewing the reference lists of the articles we found.

The included studies were RCTs that met the following inclusion criteria: 1) Included cancer patients aged 18 years and older who underwent abdominal or pelvic surgery. Abdominal or pelvic cancer was defined as malignancies of the gastrointestinal tract (except the esophagus), genitourinary tract, and gynecological malignancies. 2) The intervention included: LMWH, UFH, DOACs, fondaparinux, LMWH plus physiotherapy, DOACs plus physiotherapy, no treatment (including placebo), and physiotherapy. Physiotherapy refers to measures aimed at preventing and controlling thrombus formation through the use of specific physical devices and techniques. These methods facilitate the acceleration of venous return in the lower limbs and reduce blood stasis through mechanical principles, thereby lowering the risk of deep vein thrombosis (DVT) in high-risk populations. The physical prevention methods included in this study were: graduated compression stockings (GCS) and intermittent pneumatic compression (IPC), elastic stockings (ES), sequential compression devices (SCD), external pneumatic compression (EPS). 3) The outcomes collected included the primary outcomes of venous thromboembolism and major bleeding events, and the secondary outcomes of bleeding and adverse events. VTE was defined as a composite of PE and DVT, including symptomatic and asymptomatic cases. The occurrence of VTE required confirmation through diagnosis. Diagnosis of PE included CT scan, D-dimer, CT pulmonary angiography, and ventilation/perfusion scan. Diagnosis of asymptomatic or symptomatic DVT included color Doppler ultrasound, venography, D-dimer testing, CT venography, magnetic resonance venography, radioactive iodine fibrinogen uptake test, and impedance plethysmography (16). Major bleeding events, bleeding events, and adverse events were defined according to the criteria in different studies (bleeding events considered all bleeding, including major bleeding, while adverse events considered other adverse events excluding bleeding events). We accepted the authors’ definitions. Initially, all-cause mortality was included as an outcome, but this endpoint was ultimately not analyzed due to most study reports having a follow-up time of 30 days, during which few deaths occurred, and because this outcome was lacking in most studies.

### 2.2 Study selection and data extraction

Two researchers independently screened the literature, study selection, extracted data, and cross-checked according to the inclusion criteria. They first excluded duplicate studies in EndNote X9.1, then screened by title and abstract, and finally determined inclusion by reviewing the full text. In case of disagreement, a third researcher resolved the differences. The data extracted from the included studies included information on the study, study design, baseline characteristics, intervention measures, Duration, follow-up time, and outcome indicators.

### 2.3 Study quality assessment

Two researchers referenced the Cochrane Handbook’s risk of bias tool to evaluate the risk of bias in the included studies. Evaluation criteria included random sequence generation, allocation concealment, blinding of participants and personnel, blinding of outcome assessment, incomplete outcome data, selective reporting, and other biases. Each criterion was judged as having low risk of bias, high risk of bias, or unclear risk of bias. Disagreements were resolved through discussion, and risk of bias graphs were generated using Review Manager 5.3.

### 2.4 Statistical analysis

We employed direct meta-analysis (DMA) combined with network meta-analysis (NMA) to compute the odds ratio (OR) for binary outcomes, selecting the 95% confidence interval (CI). For direct comparisons of ≥2 RCTs, fixed-effects pairwise meta-analysis was conducted. When I^2^ > 50% and P < 0.05 indicated heterogeneity among studies, a random-effects model was chosen. If substantial heterogeneity was observed, the sources of heterogeneity were explored, and sensitivity analyses and subgroup analyses were performed. If heterogeneity among studies could not be reduced, only descriptive analysis of the obtained results was conducted.

We performed the Bayesian NMA using the BUGSnet package and the Gemtc package in R, version 4.3.2. The optimal effect model was determined based on leverage plots and Deviance Information Criterion (DIC). Convergence of iterations and stability of the model were assessed using trace plots, density plots, and Potential Scale Reduction Factor (PSRF). In cases of closed loops, we tested the assumption of transitivity, examining whether direct and indirect comparisons were consistent. Systematic analysis was conducted using node-splitting methods. Consistency was deemed good if P > 0.05, indicating consistency, while P < 0.05 indicated inconsistency among nodes. To calculate the probability ranking of each intervention and assess the likelihood of each intervention being ranked first, the probabilities were aggregated and reported as the surface under the cumulative ranking curve (SUCRA). SUCRA values range from 100% for the most favorable intervention to 0% for the least favorable intervention. When more than 10 clinical trials were included, publication bias was assessed.

## 3 Results

### 3.1 Search results and study characteristics

**Figure 1:**
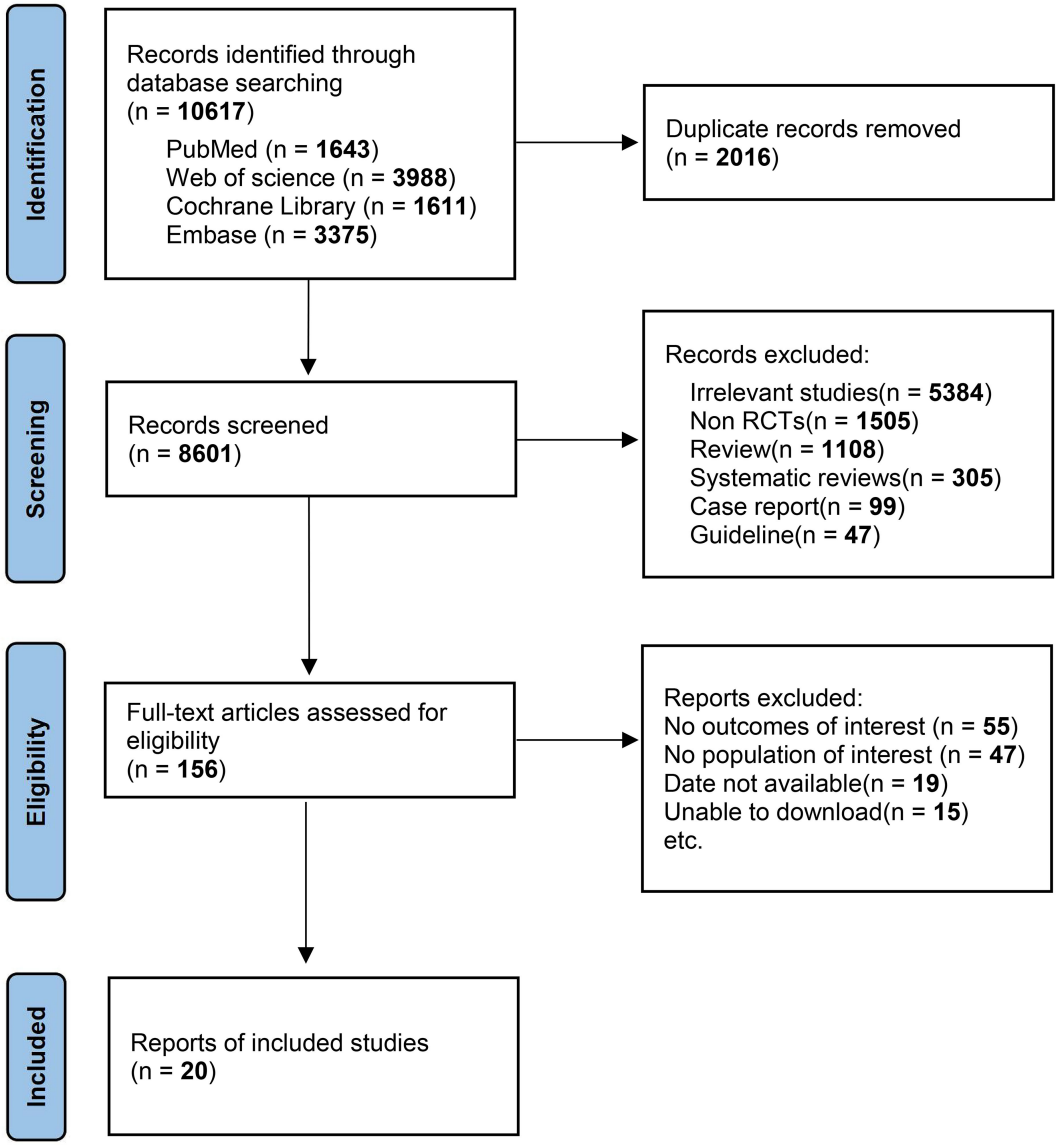
Flow chart of the study selection process.

The research process is illustrated in **Figure 1**. Initially, 10,587 articles were screened, following the initial screening that excluded drugs such as coumarins and antiplatelet agents, as well as repetitive and irrelevant studies, 156 records remained. Subsequently, after a full-text review, 20 randomized controlled trials (12–14, 17–33) were included, involving a total of 4,923 patients undergoing abdominal and pelvic cancer surgery. The clinical and methodological characteristics of these included studies are detailed in **Supplementary Table 3**. Six prophylactic venous thromboembolism (VTE) regimens were included, with an no treatment group serving as the control, comprising 4 monotherapies and 2 combination therapies involving physiotherapy. Monotherapy regimens included DOACs, LMWH, UFH, and physiotherapy, while combination therapies included DOACs plus physiotherapy and LMWH plus physiotherapy. Studies related to fondaparinux were excluded as they did not meet the inclusion criteria. Baseline characteristics of the patients are presented in **Supplementary Table 4**, with the participants’ mean age ranging from 60.63 to 63.61 years, mean BMI of 24.89 kg/m2, mean surgical duration ranging from approximately 3.3 to 3.4 hours, and the observation period ranging from 7 to 42 days postoperatively.

### 3.2 Risk of bias

Quality assessment was conducted for 20 studies based on random sequence generation, allocation concealment, blinding of participants and personnel, blinding of outcome assessment, incomplete outcome data, selective reporting, and other biases. One study (23) was deemed to have a “high risk of bias” due to subjective factors affecting the intervention implementation and failure to meet allocation concealment. Another study (33) was rated as “high risk of bias” due to incomplete outcomes. The remaining studies were categorized as “low risk of bias” or “unclear risk of bias” for each criterion based on outcome reporting. Detailed quality assessments for each outcome indicator in the studies can be found in **Supplementary Figures S6–S9**.

### 3.3 Direct meta-analysis

Meta-analyses were performed for direct comparisons containing 2 or more RCTs. In addition, direct meta-analyses of LMWH vs LMWH plus physiotherapy and LMWH plus physiotherapy vs DOACs plus physiotherapy had also been conducted in order to compare the effect of drug combined with mechanical prevention of VTE. A total of 20 direct comparisons were generated, of which 2 from VTE outcome showed heterogeneity: no treatment vs UFH (I^2^ = 77%) and physiotherapy vs LMWH (I^2^ = 62%). Two direct comparisons showed significant results: LMWH plus physiotherapy demonstrated significantly more efficacy in preventing VTE than LMWH alone, with an OR of 10.95 (1.33 to 90.40), and LMWH was superior to no treatment in preventing VTE 1.96 (1.21 to 3.19). No heterogeneity was observed in the other direct comparison results, and the differences were not statistically significant. DMA results can be found in **Supplementary Figures S1–S5**.

### 3.4 Network meta-analysis

**Figure 2:**
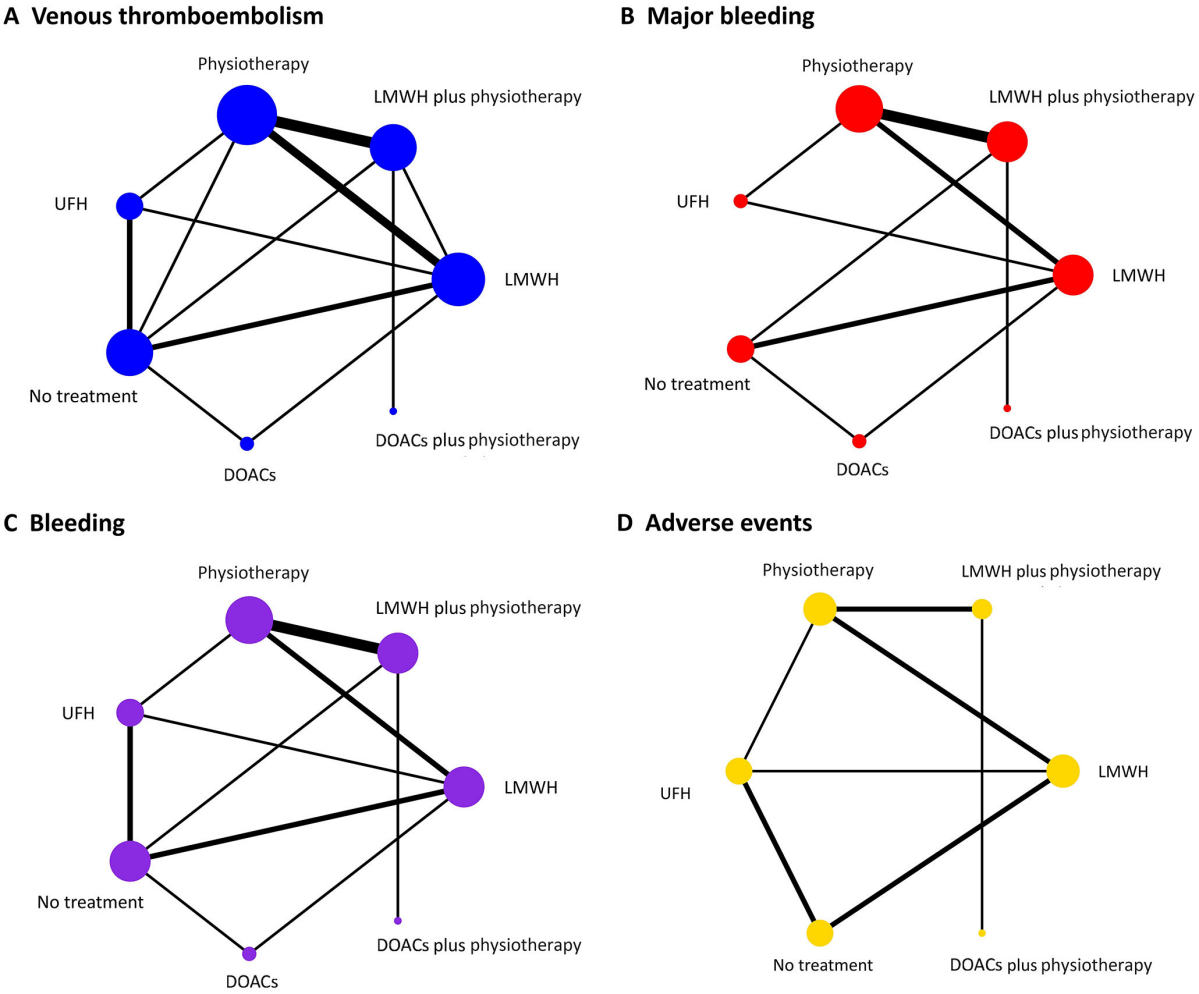
Network plots of primary and secondary outcomes. **(A)** Venous thromboembolism; **(B)** Major bleeding; **(C)** Bleeding; **(D)** Adverse events. Each node indicates a treatment modality and the straight line between two nodes indicates a direct comparison. The size of nodes and the thickness of lines between nodes are directly proportional to the sample size and research quantity. LMWH, low molecular weight heparin; UFH, unfractionated heparin; DOACs, direct oral anticoagulants.

We performed a network meta-analysis of 20 randomized trials and separately conducted direct meta-analysis for potentially aberrant outcomes (RCT<2). Convergence diagnostics indicated that PSRF approached 1, with stable fluctuations, suggesting satisfactory convergence and model stability (**Supplementary Figures S12–S15**). The network evidence plot (**Figure 2**) revealed the presence of closed loops, prompting node-splitting analysis, the results showed inconsistency between the direct and indirect comparisons of LMWH vs LMWH plus physiotherapy in the VTE outcome indicators (P < 0.05), while consistency was good for the remaining outcomes at each node (P > 0.05) (**Supplementary Figures S16–S19**).

**Figure 3:**
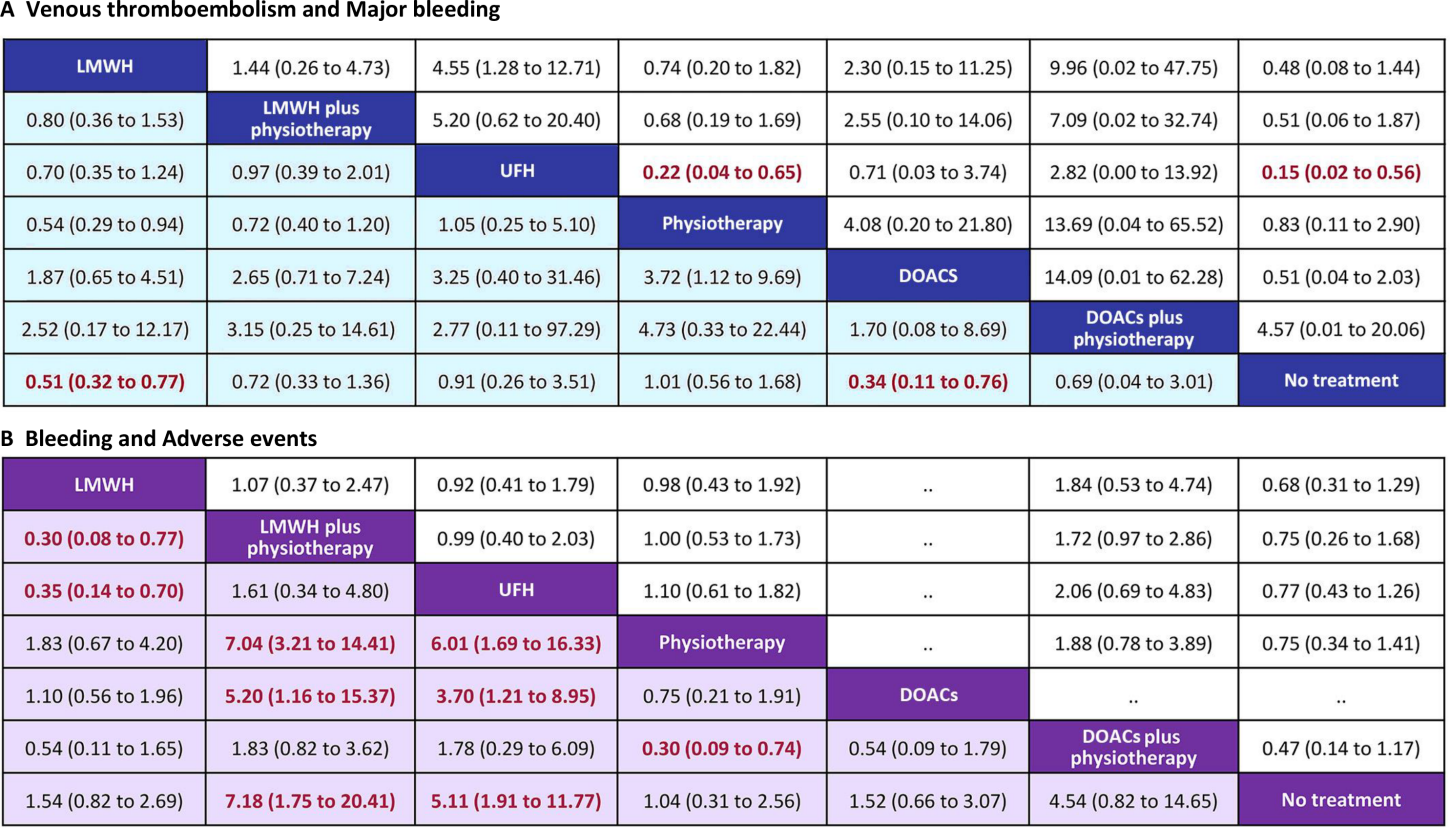
Summary of the four outcomes of Bayesian network meta-analysis. **(A)** venous thromboembolism (lower purple triangle) and major bleeding (upper white triangle); **(B)** bleeding (lower purple triangle) and adverse events (upper white triangle). Effect sizes are presented as OR of means with 95% Crl. Figure should be read from left to right, OR<1 favour the column-deffning treatment and means that the treatment in the column is associated with lower risk for the outcome than the treatment in the row. To obtain the reverse comparison OR value, reciprocals should be taken. Significant results are indicated by red bold font. LMWH, low molecular weight heparin; UFH, unfractionated heparin; DOACs, direct oral anticoagulants; OR, odds ratio; Crl, credible interval.

**Figure 4:**
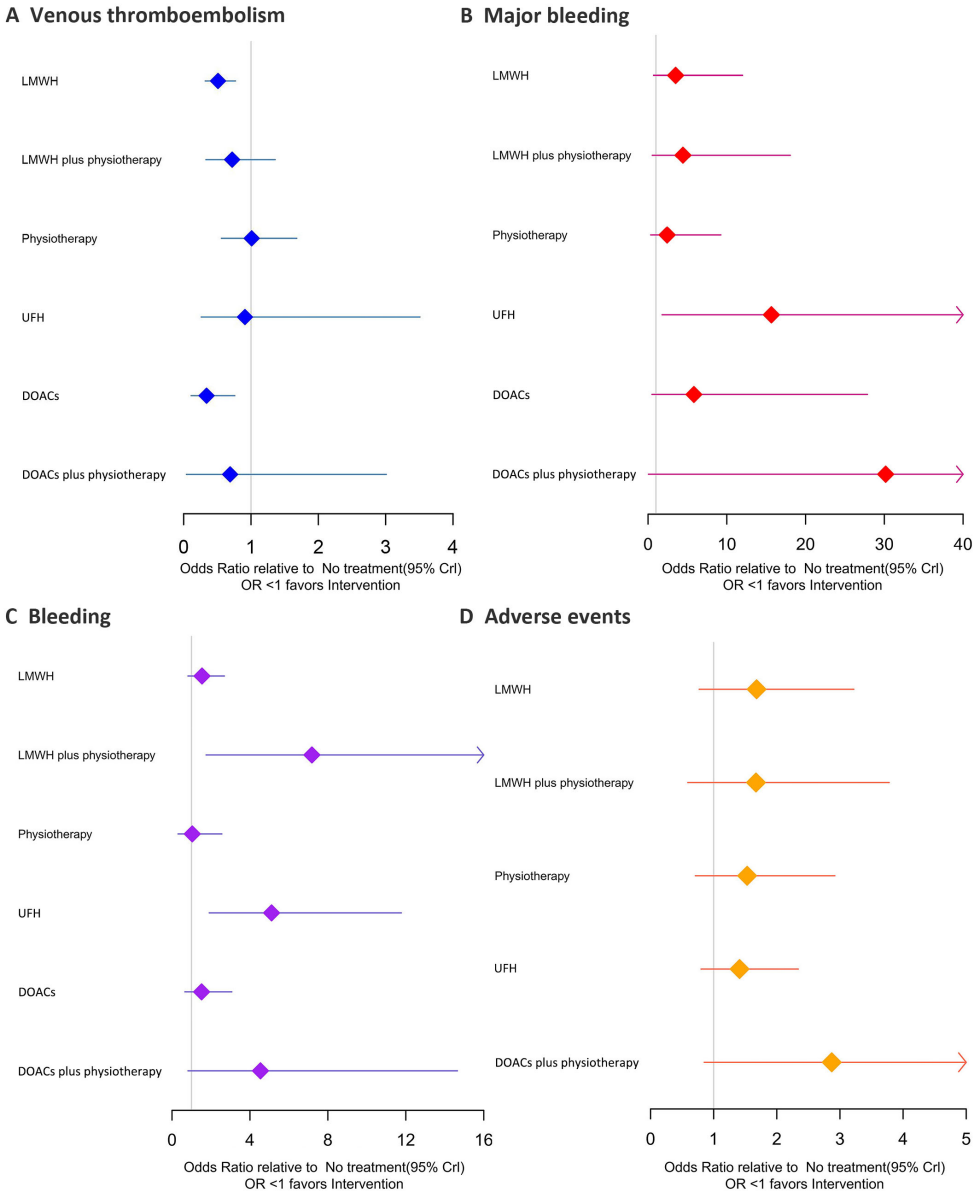
Forest plots of the four outcomes. **(A)** Venous thromboembolism; **(B)** Major bleeding; **(C)** Bleeding; **(D)** Adverse events. Effect sizes are presented as OR of means with 95% Crl. LMWH, low molecular weight heparin; UFH, unfractionated heparin; DOACs, direct oral anticoagulants; OR, odds ratio; Crl, credible interval.

**Figure 5:**
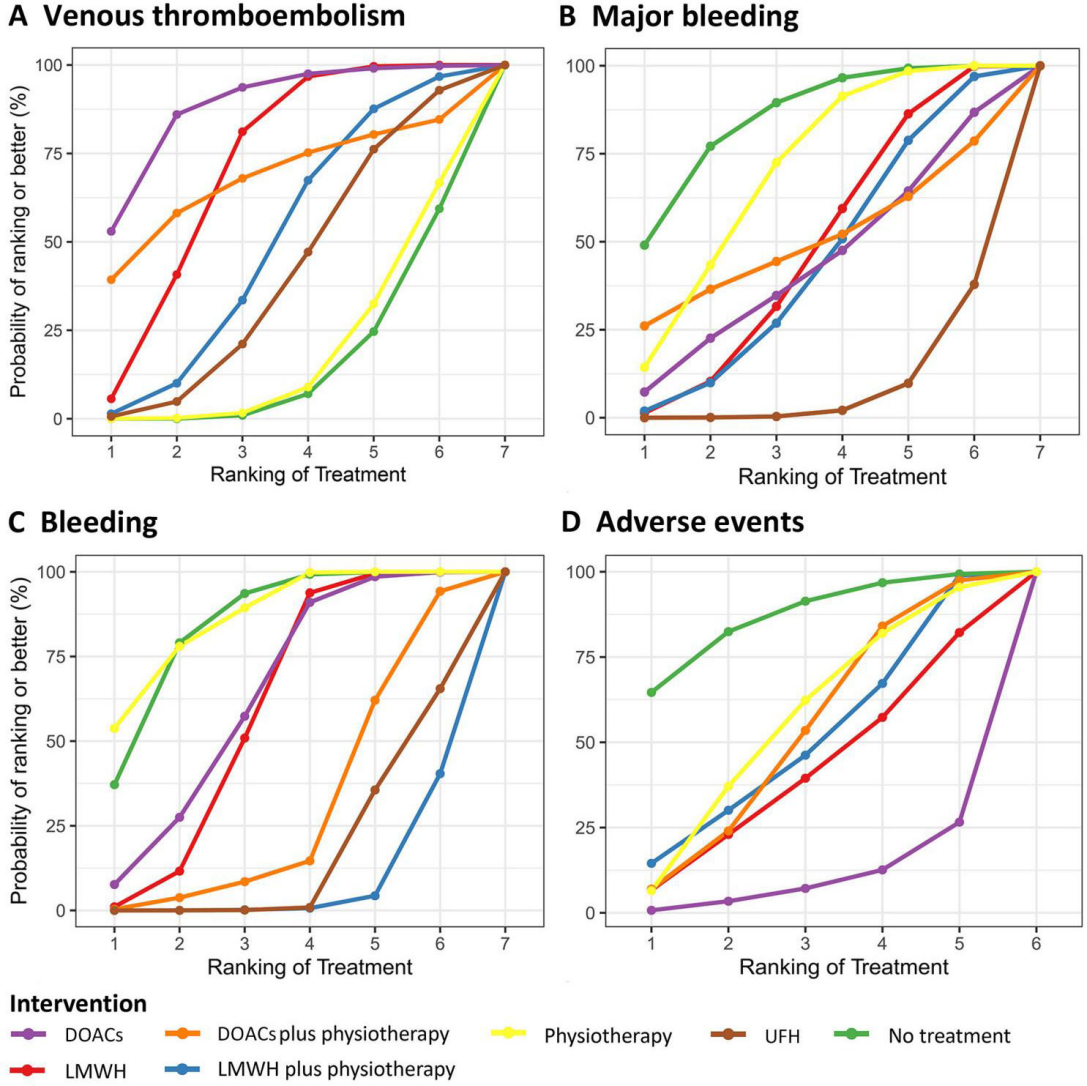
SUCRA for primary and secondary outcomes. **(A)** Venous thromboembolism; **(B)** Major bleeding; **(C)** Bleeding; **(D)** Adverse events. Figure displays the probabilities of ranking first to seventh or eighth. The larger the area under the curve, the higher the ranking and better the efficacy. SUCRA, surface under the cumulative ranking curve; LMWH, low molecular weight heparin; UFH, unfractionated heparin; DOACs, direct oral anticoagulants.

A NMA of 19 RCTs on VTE was showed that (**Figures 3**–**5**) among the four monotherapy regimens, DOACs and LMWH were the two most effective strategies for preventing VTE, with respective OR (95% CI) of 0.34 (0.11 to 0.76) and 0.51 (0.32 to 0.77) compared to no treatment, the differences were statistically significant. The results of direct comparison of the two combination regimens were selected due to the influence of error (**Supplementary Figure S5**). LMWH plus physiotherapy demonstrated significantly superior efficacy in preventing VTE compared to LMWH alone, with an OR of 10.95 (1.33 to 90.40). DOACs plus physiotherapy showed a potentially better VTE prevention effect than LMWH plus physiotherapy, but the difference was not statistically significant with an OR of 0.64 (0.11, 3.85). The top ranked interventions in the SUCRA were DOACs, LMWH, DOACs plus physiotherapy.

Results of 14 studies of major bleeding showed that (**Figures 3**–**5**) revealed that among the various interventions, physiotherapy exhibited the lowest rate of major bleeding compared to no treatment, with an OR (95% CI) of 0.83 (0.11 to 2.90), indicating no statistically significant difference. UFH had a significantly higher rate of major bleeding compared to no treatment, physiotherapy, and LMWH, with ORs of 0.15 (0.02 to 0.56), 0.22 (0.04 to 0.56), and 4.55 (1.28 to 12.71), respectively. There was no significant difference between LMWH plus physiotherapy and LMWH 1.44 (0.26 to 4.73). In direct comparisons, LMWH plus physiotherapy was associated with a higher incidence of major bleeding than DOACs plus physiotherapy (**Supplementary Figure S5**), but the difference was not statistically significant, with an OR of 0.96 (0.06 to 15.45). The top three active treatments in lowering major bleeding were physiotherapy, LMWH, DOACs plus physiotherapy.

Secondary safety results showed that (**Figures 3**–**5**) in terms of bleeding, compared with no treatment, UFH and LMWH had the highest probability of bleeding, with an OR (95% CI) of 5.11 (1.91 to 11.77) and 7.18 (1.75 to 20.41). The best intervention was physiotherapy. In terms of adverse events, there were no statistically significant differences among the 6 drug comparisons, with UFH identified as the optimal intervention.

### 3.5 Subgroup and sensitivity analyses

Subgroup analyses of the duration of thromboprophylaxis and the location of cancer development are shown in **Supplementary Figures S20, S21**. Sensitivity analysis was performed by excluding study Maxwell 2001 (24), and the heterogeneity of physiotherapy versus LMWH was reduced to 0 (**Supplementary Figure S22**).

### 3.6 Publication bias

The publication bias analysis results were presented using funnel plots. The outcome measure of venous thromboembolism showed a slight asymmetry in the funnel plot, while the funnel plots for the other outcome measures were generally symmetric, indicating no apparent publication bias (**Supplementary Figures S23, S24**).

## 4 Discussion

VTE is a serious complication after abdominal and pelvic cancer surgery, which not only affects patient prognosis and interferes with chemotherapy regimens but also increases the risk of death (2). The main pathogenesis of VTE involves stasis of blood flow, hypercoagulability, and endothelial injury. The prolonged postoperative recovery makes the venous return of the lower limbs slow and blood flow stasis, and tumor factors increase the procoagulant component. Preventive strategies should be taken to prevent postoperative VTE in clinical practice. Heparins (such as LMWH, UFH, fondaparinux) are commonly used anticoagulants in clinical practice. They exert anticoagulant effects by significantly enhancing the affinity of antithrombin III for coagulation factors, leading to the immediate inactivation of thrombin. DOACs is a novel alternative choice for the prevention and treatment of thromboembolic diseases. It can exert anticoagulant effects by inhibiting individual coagulation factors in the coagulation cascade, affecting two critical targets in the process of thrombus formation, namely factor IIa and factor Xa, thereby achieving rapid onset of action and high bioavailability. physiotherapy can effectively increase patients’ venous blood flow velocity, promote blood circulation, reduce blood stasis to improve the hypercoagulable state of blood flow, thus achieving the goal of preventing VTE.

This study employed NMA and DMA to investigate the effectiveness and safety of six preventive strategies in postoperative thromboprophylaxis for abdominal pelvic cancer. The aim was to identify the most effective and safe preventive strategy with minimal adverse effects. Following stringent inclusion criteria, a total of 20 randomized controlled trials (RCTs) involving 4923 patients were included. Our study results indicated that among monotherapy regimens, DOACs demonstrated the highest efficacy in preventing postoperative thrombosis, followed by LMWH. physiotherapy demonstrated the best safety profile in terms of major bleeding and bleeding events, while UFH had the best safety profile in terms of adverse events. Among combination regimens, DMA results indicated that DOACs plus physiotherapy had the best efficacy in preventing thrombosis and remained the safest in terms of major bleeding and bleeding events. Regarding whether medications should be combined with physical prevention regimens, we found LMWH plus physiotherapy showed superior efficacy over LMWH in direct comparison but inferior efficacy to LMWH in indirect comparison, there is inconsistency. Therefore, we chose to report the more reliable DMA results, and considered that in the prevention of VTE, LMWH plus physiotherapy was more effective than LMWH alone; As a small sample intervention, only one literature was included, DOACs plus physiotherapy did not show advantages compared with DOACs, which may be related to the small sample size and the analysis accuracy of the program package. Currently, more studies support the use of combination therapy rather than monotherapy. The ASH expert panel has suggested that combination therapy with medication and physiotherapy is more favorable for thromboprophylaxis. It is necessary to apply drug combined with physical prevention in patients with high risk of VTE. With respect to safety, we was found that LMWH plus physiotherapy might increase the risk of bleeding. ASH indicated that compared to monotherapy, combination thromboprophylaxis may potentially increase the risk of major bleeding, although there was minimal difference in bleeding outcomes between the two in other studies, the evidence remains highly uncertain, and therefore, the assessment of their safety showed no significant differences (9). We believe that physiotherapy alone is not suitable as a preventive measure without a high risk of bleeding. Patients with abdominal and pelvic cancer often face a high risk of VTE after surgery. In this study, physiotherapy has the same effect on preventing VTE compared with no treatment, and SUCRA results rank the bottom. Physiotherapy alone is difficult to meet the anticoagulation needs and does not effectively reduce the incidence of postoperative thrombosis. On the basis of anticoagulant drug prevention, IPC prevention can bring more benefits to patients. In addition, the effectiveness of physiotherapy in preventing VTE also varies according to the type of device used, and the different equipment conditions in different medical institutions may cause differences in the preventive effect of physiotherapy. A meta-analysis (34) of 70 trials showed that IPC was more effective than antithrombotic GCS in reducing DVT and that IPC was equivalent to drug prevention and associated with a lower risk of bleeding, which also reflects some differences in physiotherapy. For patients at extremely high risk of bleeding or those who have experienced bleeding events, consideration should be given to the use of physical preventive measures to mitigate the risk of bleeding or re-bleeding.

In terms of anticoagulant drug selection, LMWH and UFH have been recommended as first-line medications in many guidelines (2, 7–11). DOACs have a lower level of recommendation due to lack of supporting evidence. ASCO (11) recommends apixaban or rivaroxaban as a prophylactic regimen following initial LMWH or UFH treatment. NCCN (10) only recommends apixaban for prevention in gynecological cancer surgery, mainly due to limited available data. Our NMA supplements the evidence for the use of DOACs in preventing postoperative thrombosis in cancer patients. The results of this study show that DOACs is the best strategy we consider. In terms of effectiveness, DOACs ranks first in preventing postoperative VTE, and is more likely to reduce the risk of postoperative VTE. In terms of safety, the bleeding risk with DOACs may be lower than with LMWH. However, since the outcome measures in this study did not involved severe adverse events, the assessment of DOACs in adverse events, though not ideal. Most adverse events were mild, such as dizziness and joint pain, which did not significantly affect safety assessment and did not have a significant impact on patient health. It is worth noting that we found a significant increase in bleeding and major bleeding complications with UFH, which raises safety concerns. Caution should be exercised when using UFH in patients at high risk of bleeding. In terms of economy, the cost of DOACs is lower than that of LMWH and UFH. The cost of short-term preventive application is not high, and the cost-effectiveness is more favorable to DOACs. However, considering the risk of disability after VTE or the costs related to other syndromes will increase the cost-effectiveness of VTE prevention, and more comprehensive economic evaluation evidence is needed. Our results confirmed the effectiveness of pharmacological interventions combined with physiotherapy in preventing VTE. Among the pharmacological options, DOACs emerged as the optimal choice, and the subgroup analysis also confirmed the safety of long-term anticoagulation (over 4 weeks). However, In clinical prevention decisions, each anticoagulant agent has bleeding risks, and it is necessary to assess the bleeding risk of the patient individually and carefully consider the benefit-risk balance. Regarding the selection of DOACs drugs, rivaroxaban should be used with caution in patients at risk of major gastrointestinal bleeding, and apixaban is recommended because of the higher risk of GI complications associated with rivaroxaban (10, 35).

This study is the first to use Bayesian network analysis combined with traditional meta-analysis to evaluate the safety and efficacy of different prevention strategies for abdominal and pelvic cancer after surgery, considering the effect of DOACs plus physiotherapy as one of the prevention options. DMA supplemented the impact of inconsistency between LMWH plus physiotherapy and LMWH in this study to ensure the rigor of the results, and the sensitivity analysis results were robust. Our study demonstrated that DOACs is the best option for VTE prevention after abdominal and pelvic cancer surgery, with the optimal balance between benefits and risks. DMA analysis provided some evidence to support the use of drug plus physiotherapy in anticoagulation regimens. According to the DMA results, we found that LMWH plus physiotherapy showed significant effects. However, due to the lack of RCTs directly comparing DOACs with DOACs plus physiotherapy among the included studies, indirect comparison evidence was insufficient to fully confirm the efficacy and safety of DOACs plus physiotherapy. We also support the consideration of DOACs plus physiotherapy for VTE prevention after abdominal and pelvic cancer surgery in future clinical applications. Other limitations include: 1) This study was based on Bayesian theory and primarily evaluated the efficacy and safety of different intervention strategies, but it did not address the economic evaluation. Long-term thrombosis prevention after abdominal-pelvic cancer surgery is crucial; however, the economic costs associated with thrombosis prevention and bleeding events require a more comprehensive economic assessment. This represents one of the limitations of the current study. 2) Few included studies on DOACs, unable to specify the subdivision of DOACs. Additionally, the duration of thromboprophylaxis varied in each study. 3) In terms of study population selection, we primarily focused on patients undergoing abdominal and pelvic cancer surgeries, which are considered one of the high-risk groups for VTE. As other high-risk VTE populations, such as hospitalized patients with prolonged immobility or those undergoing orthopedic surgeries, were not included, the generalizability of our findings to all high-risk VTE populations for thromboembolism prevention is limited. This is also a limitation of our study. 4) The BUGSnet software package is unable to accurately analyze small sample studies, and the overall number of studies included in this research is limited. Specifically, the sample size and number of studies for intervention DOACs plus physiotherapy were relatively small, and there were some differences compared to other interventions. As a result, the true effect of intervention DOACs plus physiotherapy in preventing postoperative abdominal-pelvic VTE could not be fully reflected in our analysis. In the future, we hope that more studies will make up for the existing shortcomings, carry out more in-depth economic evaluation, and comprehensively optimize the thrombosis prevention strategy of abdominal and pelvic cancer surgery, which will not only improve the long-term prognosis of patients, but also provide a more scientific basis for clinical practice decision-making.

## 5 Conclusion

In summary, the traditional meta-analysis as well as the network meta-analysis results indicated that DOACs might have provided better thromboprophylaxis for postoperative abdominal pelvic cancer patients. It could achieve a better balance between efficacy and safety. The combination of pharmacological and physical therapy could achieve better anticoagulant status. Given the limitations in sample sizes across studies, these conclusions necessitate further validation through high-quality, large-scale clinical trials.

## Data Availability

The original contributions presented in the study are included in the article/**Supplementary Material**. Further inquiries can be directed to the corresponding authors.
