# Supplementary material for: Comparison of efficacy and safety of thrombus prevention strategies after abdominal and pelvic cancer surgery: Bayesian network based meta-analysis
Source: Front Oncol. 2025 Feb 11;15:1445485. doi: 10.3389/fonc.2025.1445485 (PMC11851121; doi:10.3389/fonc.2025.1445485)
Supplement: Supplementary file 1 [file DataSheet1.docx]

***Supplementary*** ***material***

Supplementary Figure 1. Results of pairwise meta-analysis of venous thromboembolism

Supplementary Figure 2. Results of pairwise meta-analysis of major bleeding

Supplementary Figure 3. Results of pairwise meta-analysis of bleeding

Supplementary Figure 4. Results of pairwise meta-analysis of adverse events

Supplementary Figure 5. Individual direct comparison

Supplementary Figure 6. Risk of bias of venous thromboembolism

Supplementary Figure 7. Risk of bias of major bleeding

Supplementary Figure 8. Risk of bias of bleeding

Supplementary Figure 9. Risk of bias of adverse events

Supplementary Figure 10. Leverage plots of random model and fixed model

Supplementary Figure 11. Leverage plots of consistency model and inconsistency model

Supplementary Figure 12. Convergence: venous thromboembolism

Supplementary Figure 13. Convergence: major bleeding

Supplementary Figure 14. Convergence: bleeding

Supplementary Figure 15. Convergence: adverse events

Supplementary Figure 16. Nodes-splitting analysis of venous thromboembolism

Supplementary Figure 17. Nodes-splitting analysis of major bleeding

Supplementary Figure 18. Nodes-splitting analysis of bleeding

Supplementary Figure 19. Nodes-splitting analysis of adverse events

Supplementary Figure 20. SUCRA plots of subgroup 1: duration of administration

Supplementary Figure 21. SUCRA plots of subgroup 2: site of cancer occurrence

Supplementary Figure 22. Forest plot for sensitivity analysis

Supplementary Figure 23. Funnel plots of venous thromboembolism and major bleeding

Supplementary Figure 24. Funnel plots of bleeding and adverse events

**
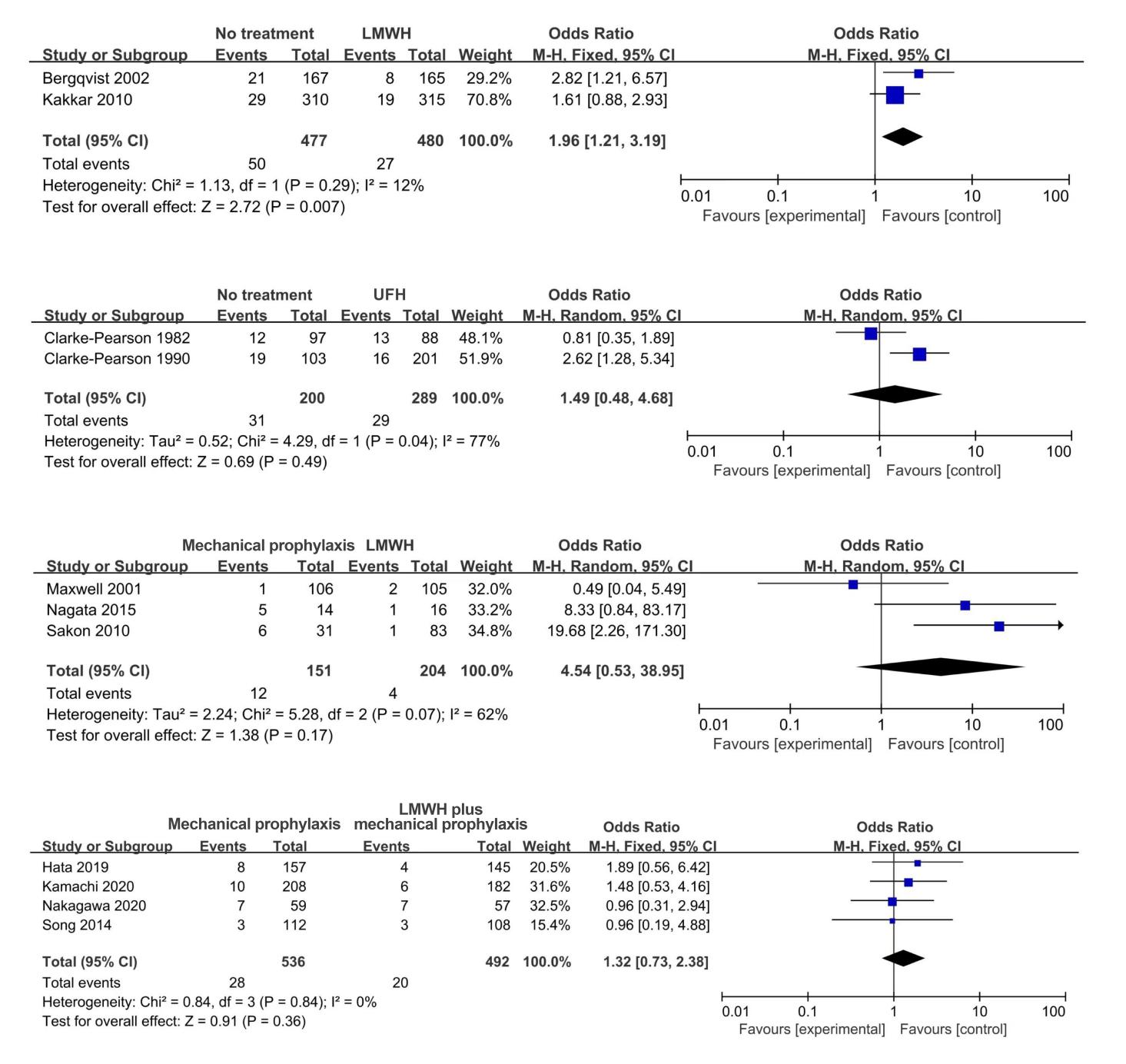
**

**Supplementary Figure 1.** Results of pairwise meta-analysis of venous thromboembolism

**Abbreviation:** LMWH: low molecular weight heparin; LMWH plus physiotherapy: low molecular weight heparin plus physiotherapy; UFH: unfractionated heparin

**Note:** From the Figure we could see, there exist significant heterogeneity between No treatment vs UFH and between Physiotherapy vs LMWH, thus we use random effect model to estimate the results Translation and subsequently, a sensitivity analysis was conducted. I^2^ > 50% indicates indicates significant heterogeneity

**
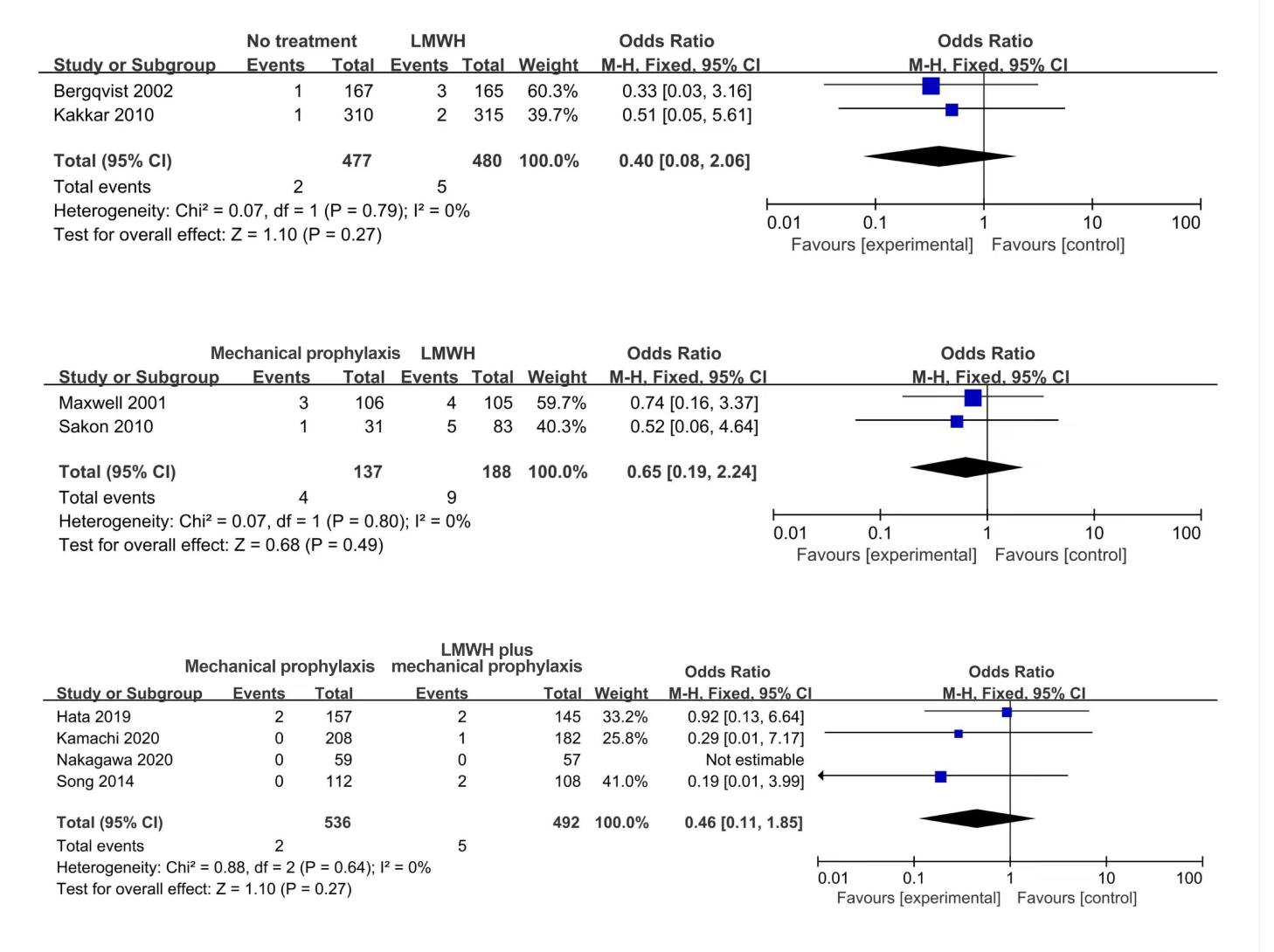
**

**Supplementary Figure 2.** Results of pairwise meta-analysis of major bleeding

**Abbreviation:** LMWH: low molecular weight heparin

**Note:** From the Figure we could see, there exists no significant heterogeneity between any two arms, which allows the use of fixed effect model to estimate the results. I^2^ ＜ 50% indicates indicates no significant heterogeneity

**
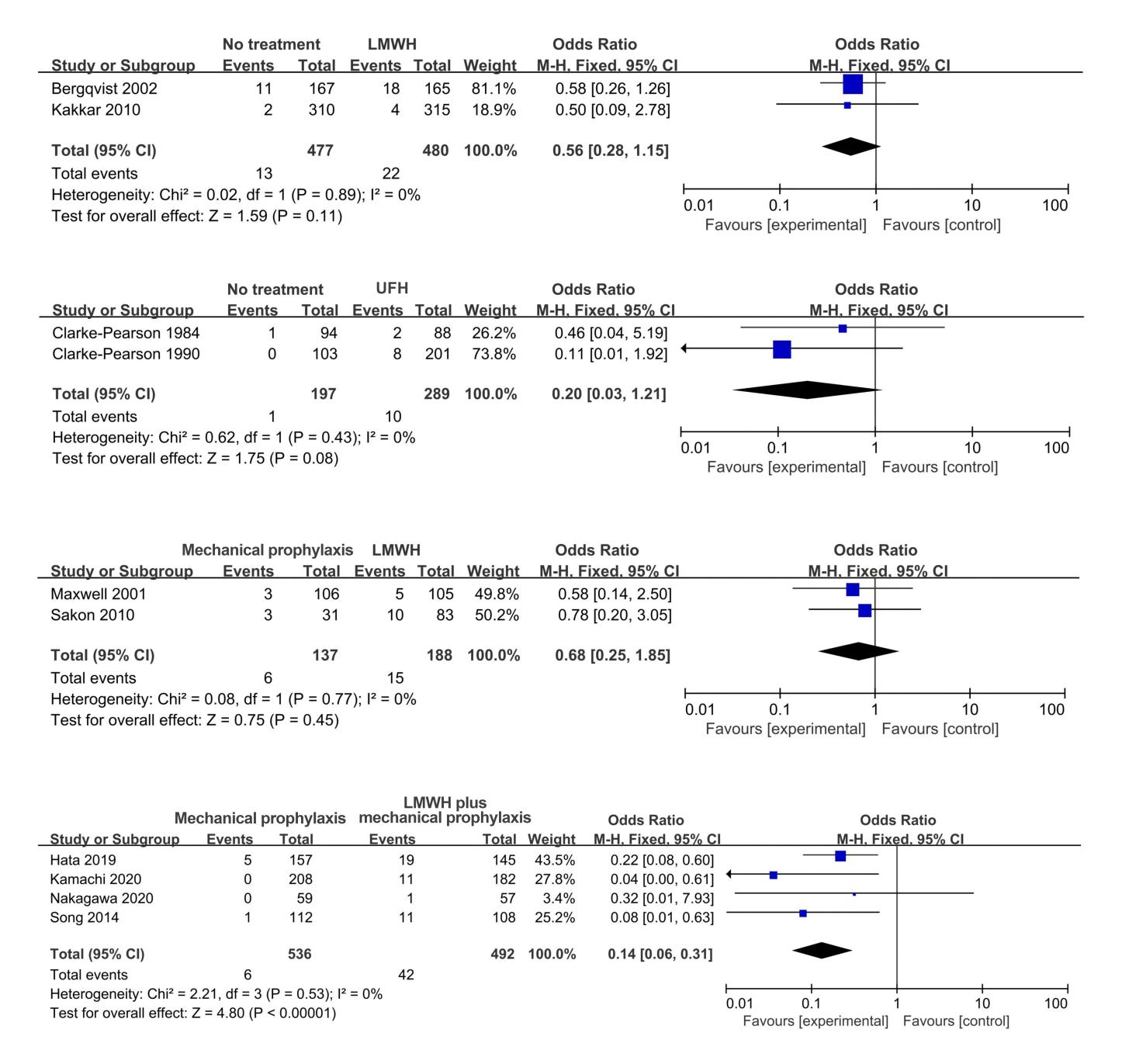
**

**Supplementary Figure 3.** Results of pairwise meta-analysis of bleeding

**Abbreviation:** LMWH: low molecular weight heparin; UFH: unfractionated heparin

**Note:** From the Figure we could see, there exists no significant heterogeneity between any two arms, which allows the use of fixed effect model to estimate the results. I^2^ ＜ 50% indicates indicates no significant heterogeneity

**
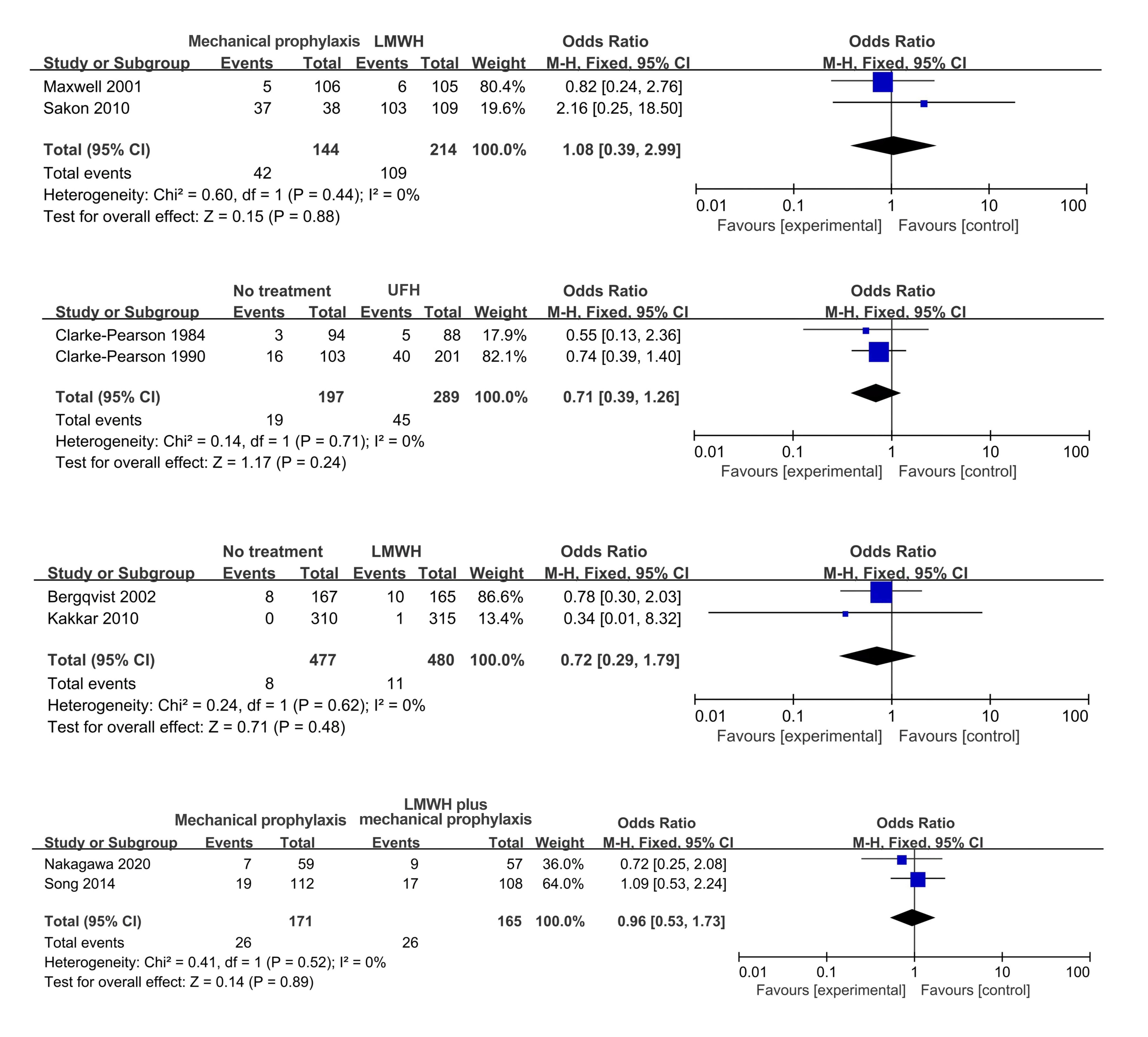
**

**Supplementary Figure 4.** Results of pairwise meta-analysis of adverse events

**Abbreviation:** LMWH: low molecular weight heparin; UFH: unfractionated heparin

**Note:** From the Figure we could see, there exists no significant heterogeneity between any two arms, which allows the use of fixed effect model to estimate the results. I^2^ ＜ 50% indicates indicates no significant heterogeneity


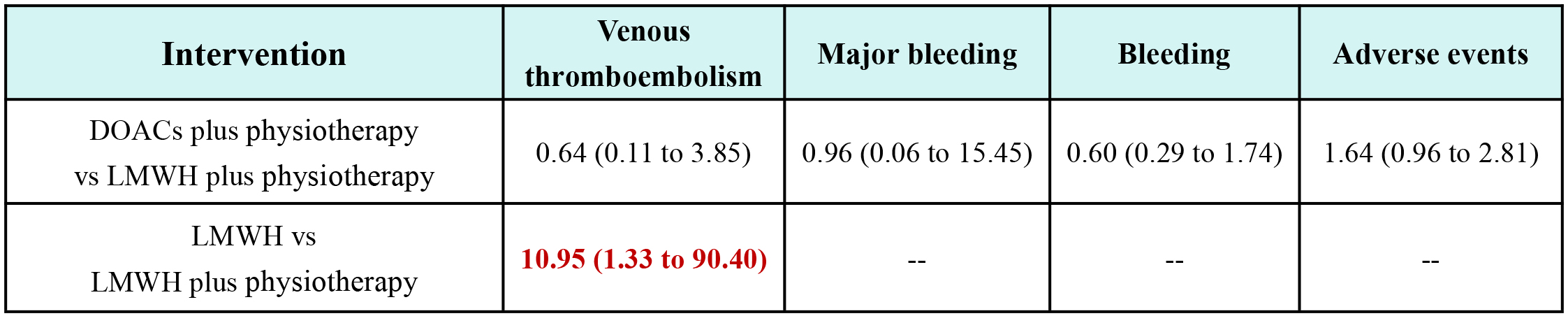


**Supplementary Figure 5.** Individual direct comparison

**Abbreviation:** LMWH: low molecular weight heparin; DOACs: direct oral anticoagulants


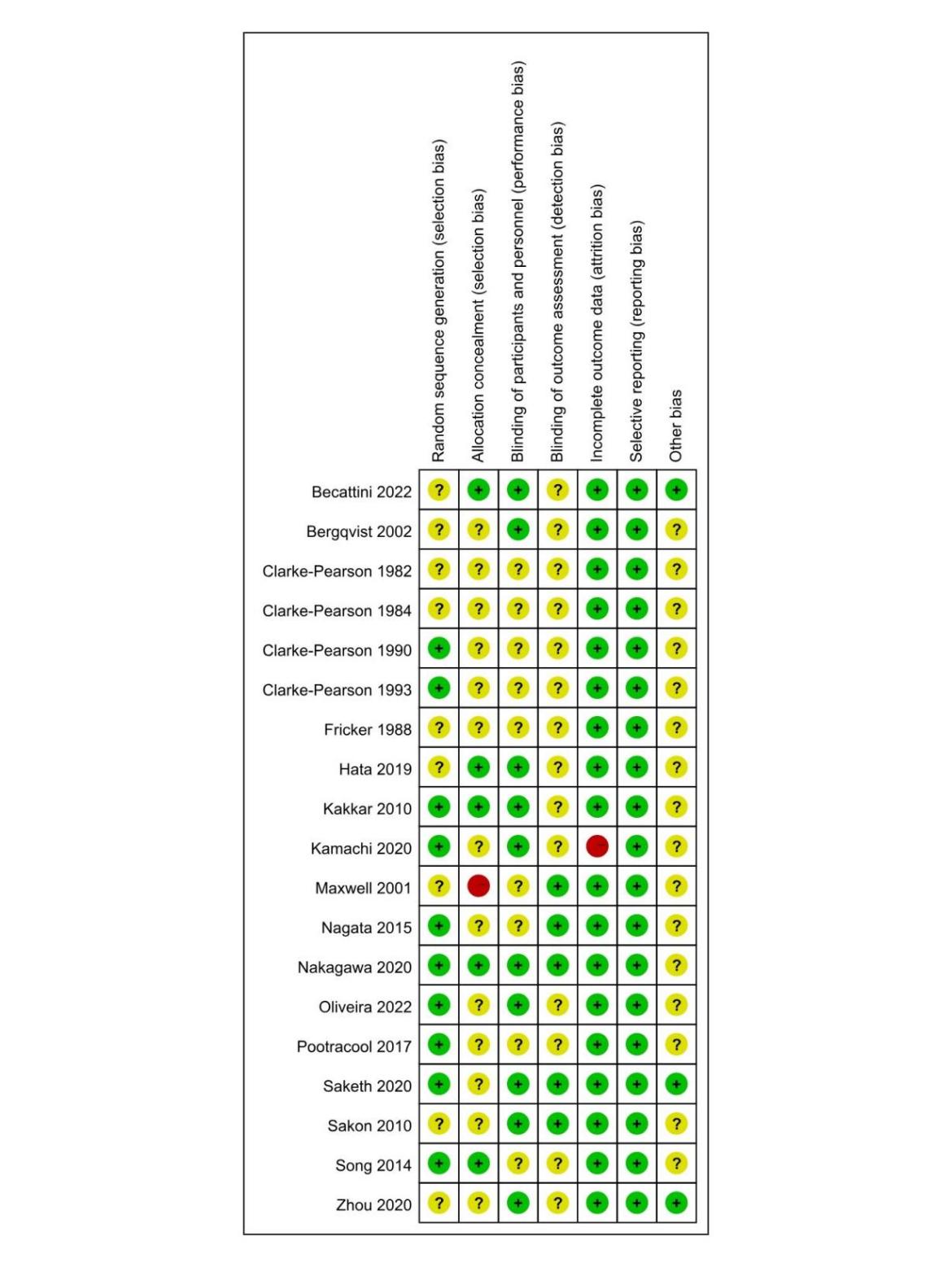


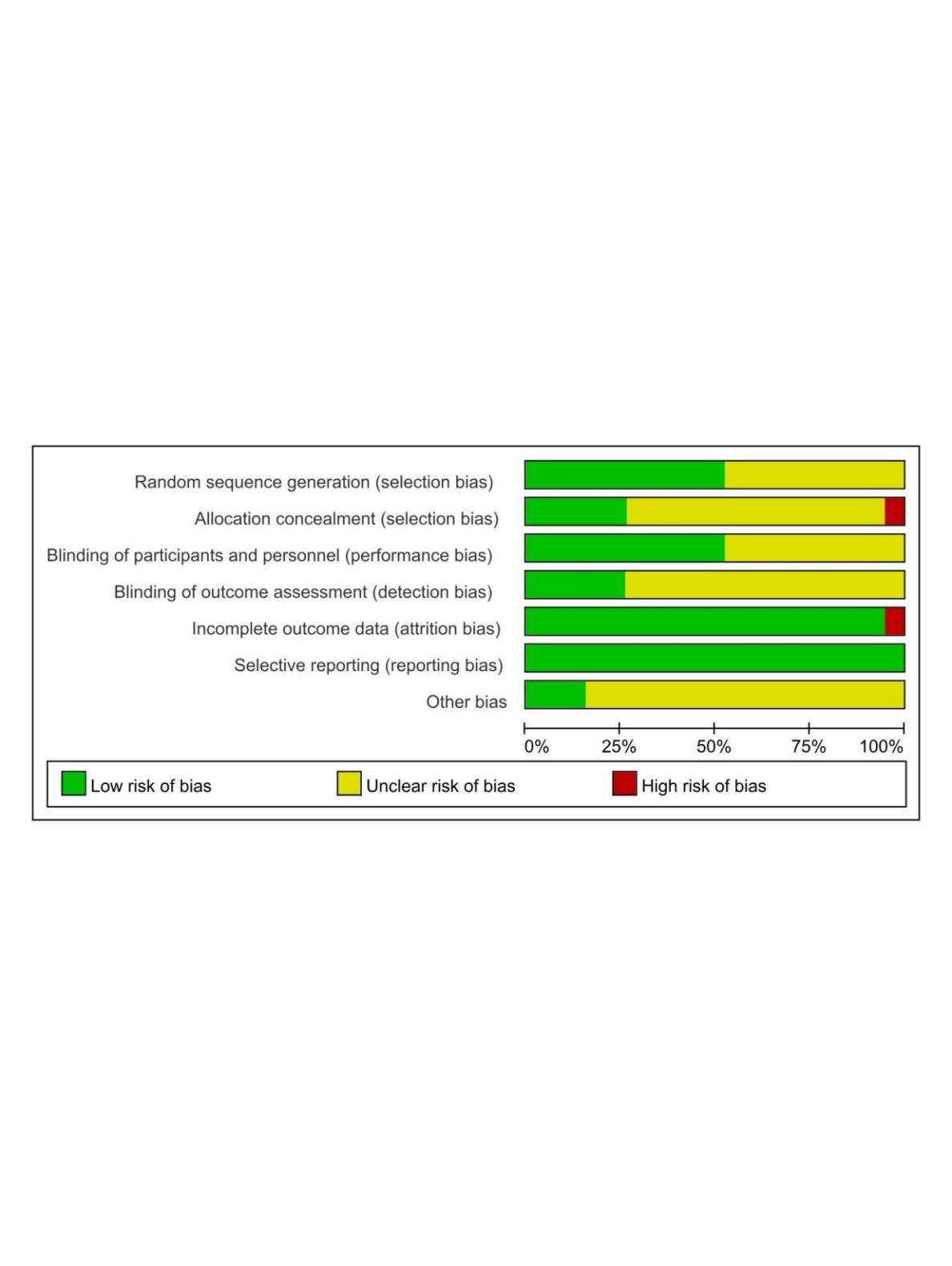


**Supplementary Figure 6.** Risk of bias of venous thromboembolism

**Note:** Green: Low risk of bias, Yellow: Unclear risk of bias, Red: High risk of bias


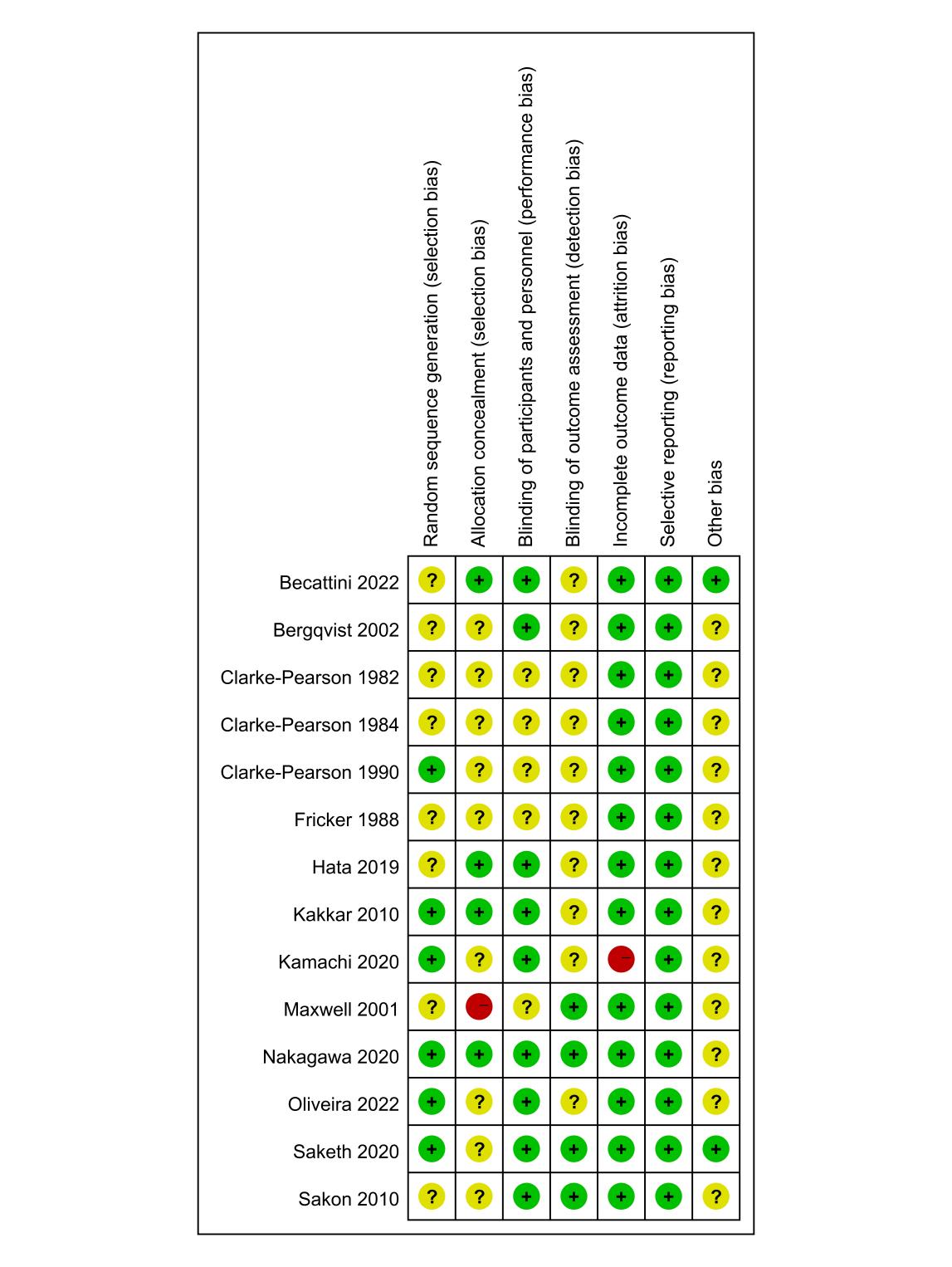


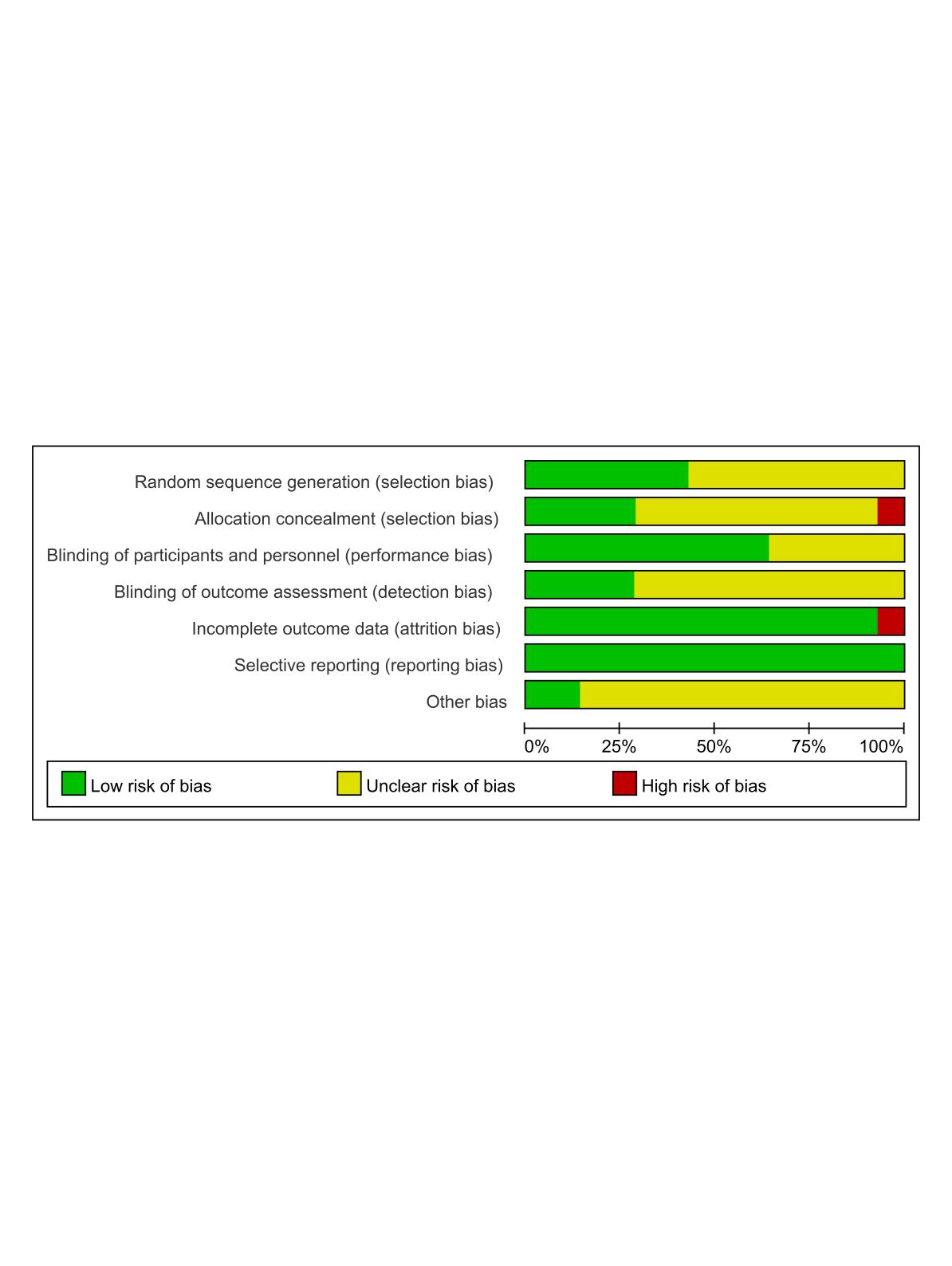


**Supplementary Figure 7.** Risk of bias of major bleeding

**Note:** Green: Low risk of bias, Yellow: Unclear risk of bias, Red: High risk of bias


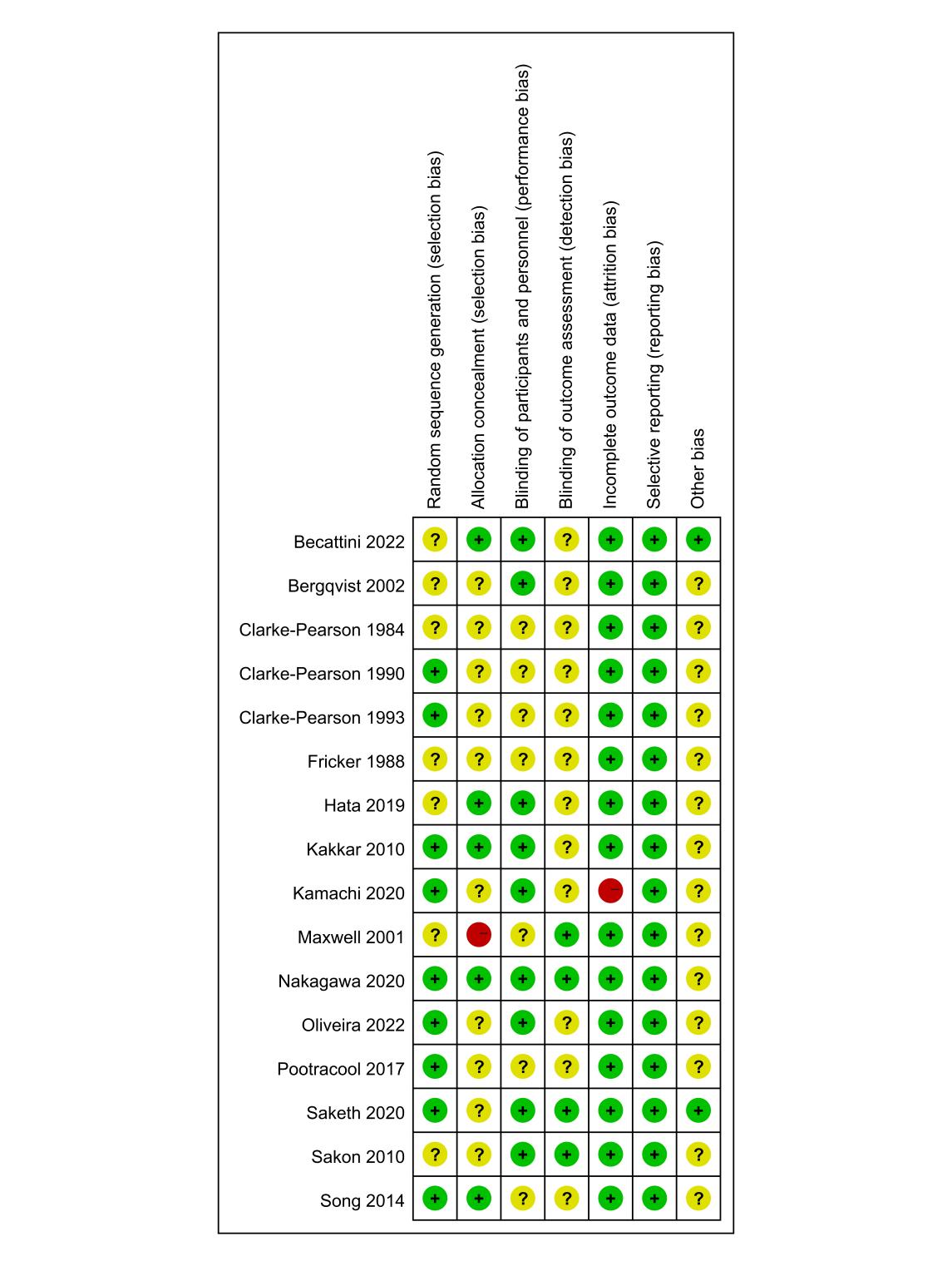

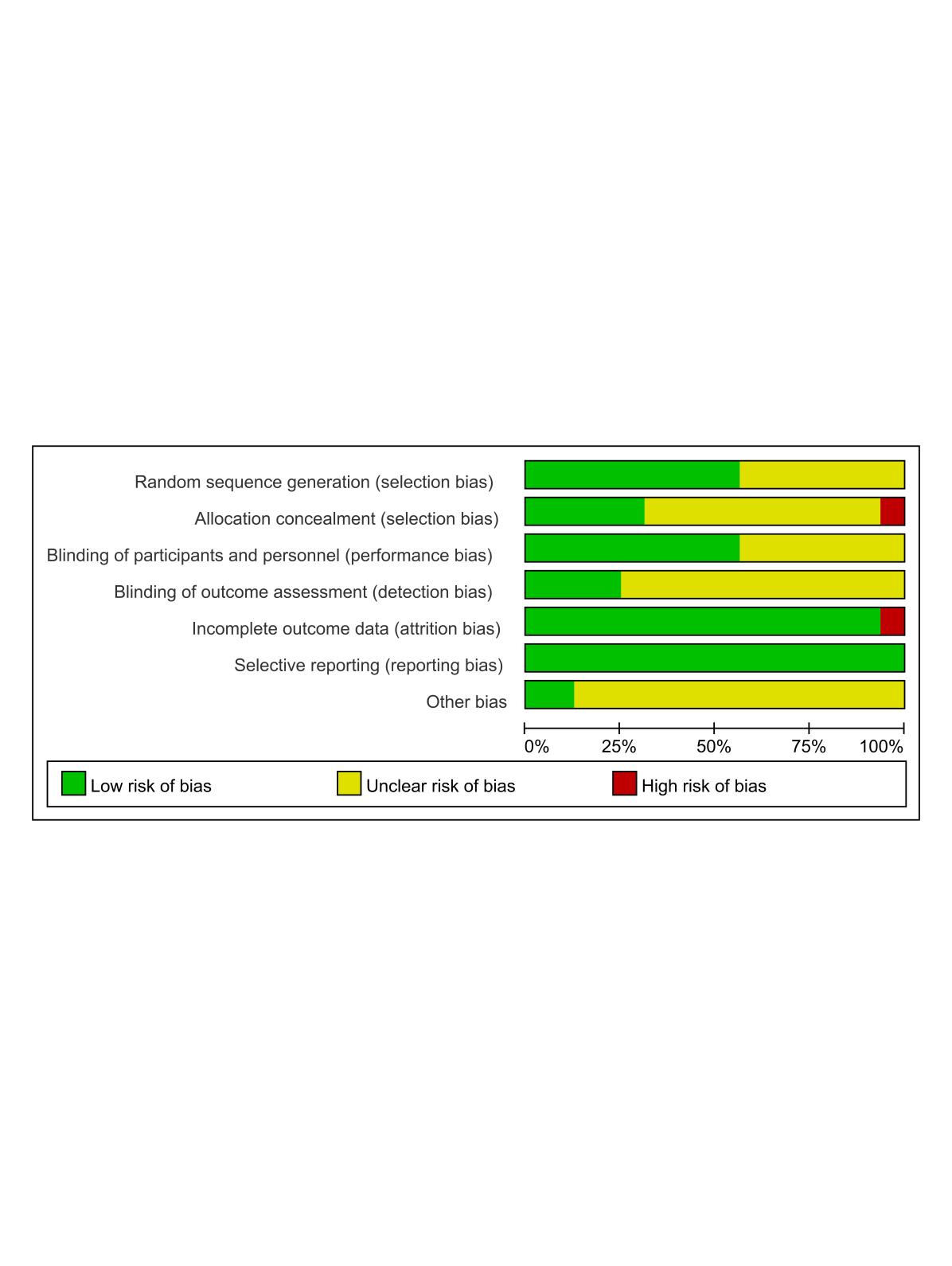


**Supplementary Figure 8.** Risk of bias of bleeding

**Note:** Green: Low risk of bias, Yellow: Unclear risk of bias, Red: High risk of bias


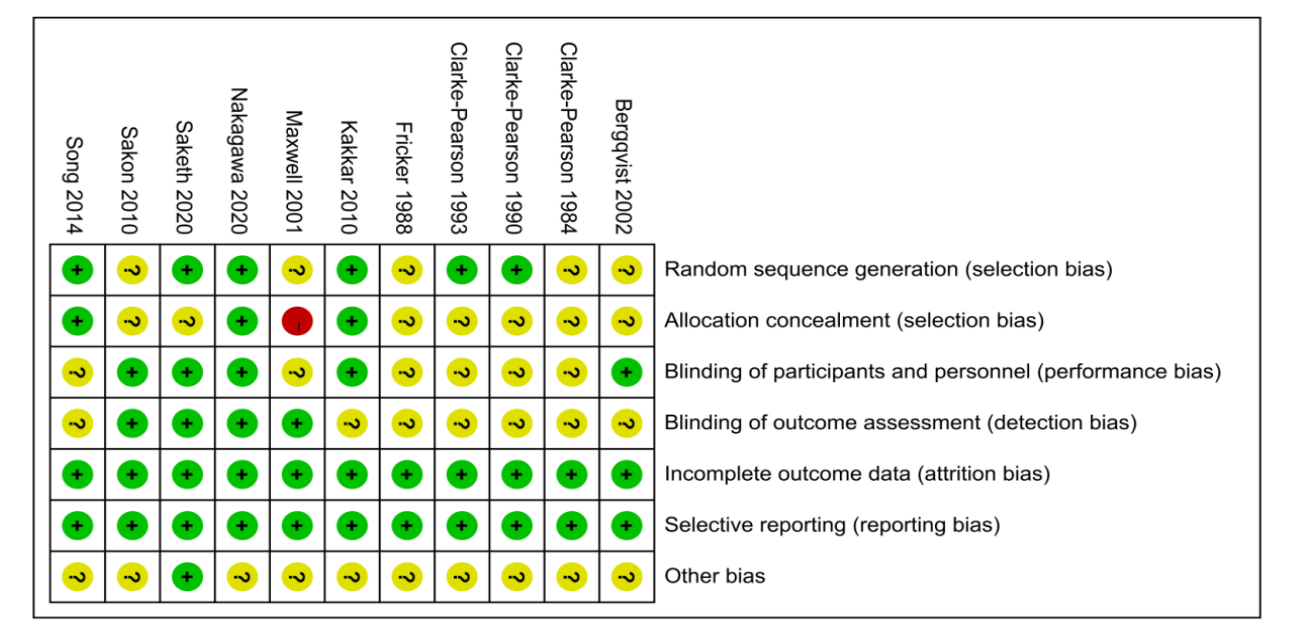


**
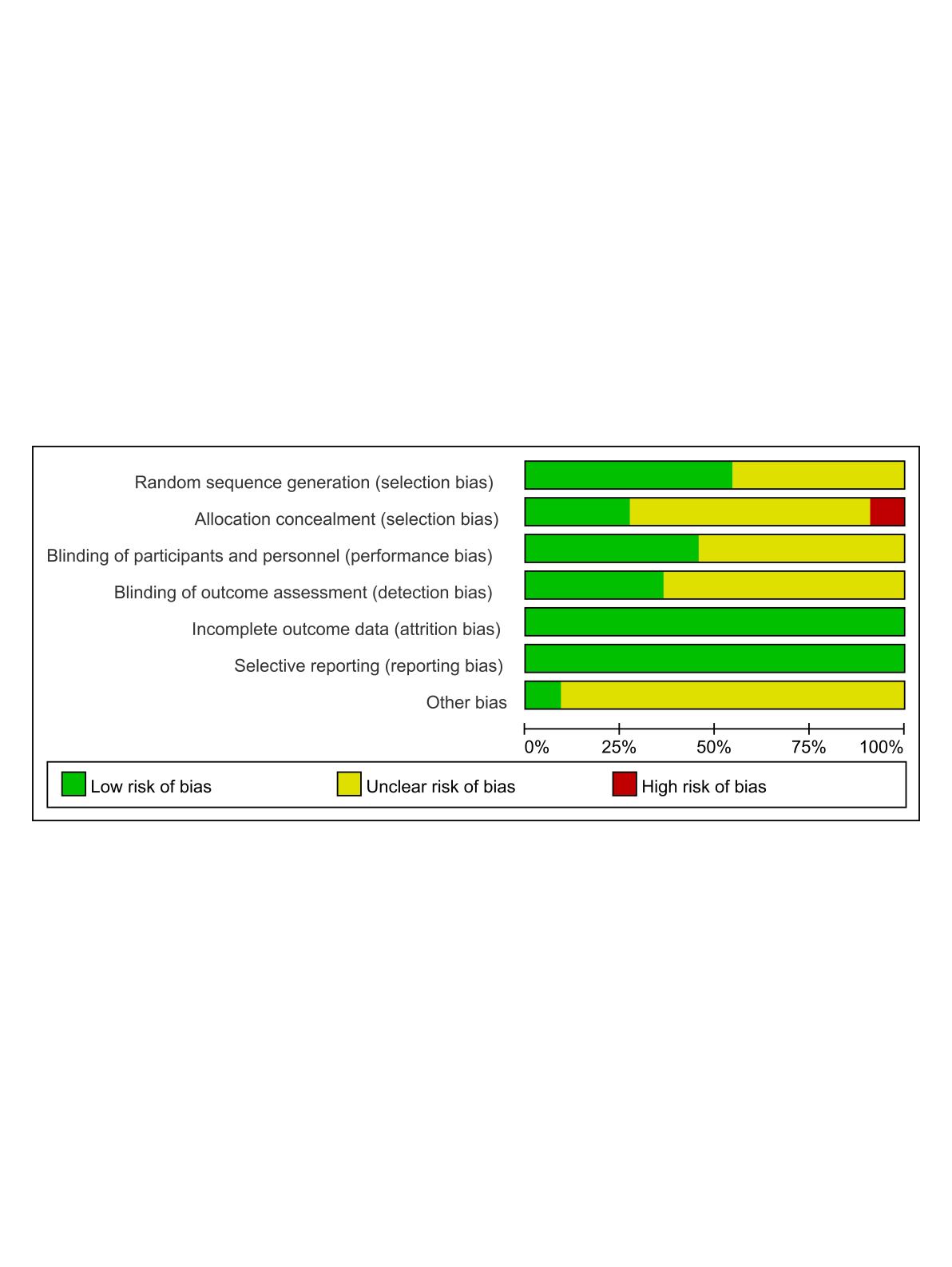
**

**Supplementary Figure 9.** Risk of bias of adverse events

**Note:** Green: Low risk of bias, Yellow: Unclear risk of bias, Red: High risk of bias

**a) Venous thromboembolism b) Major bleeding**


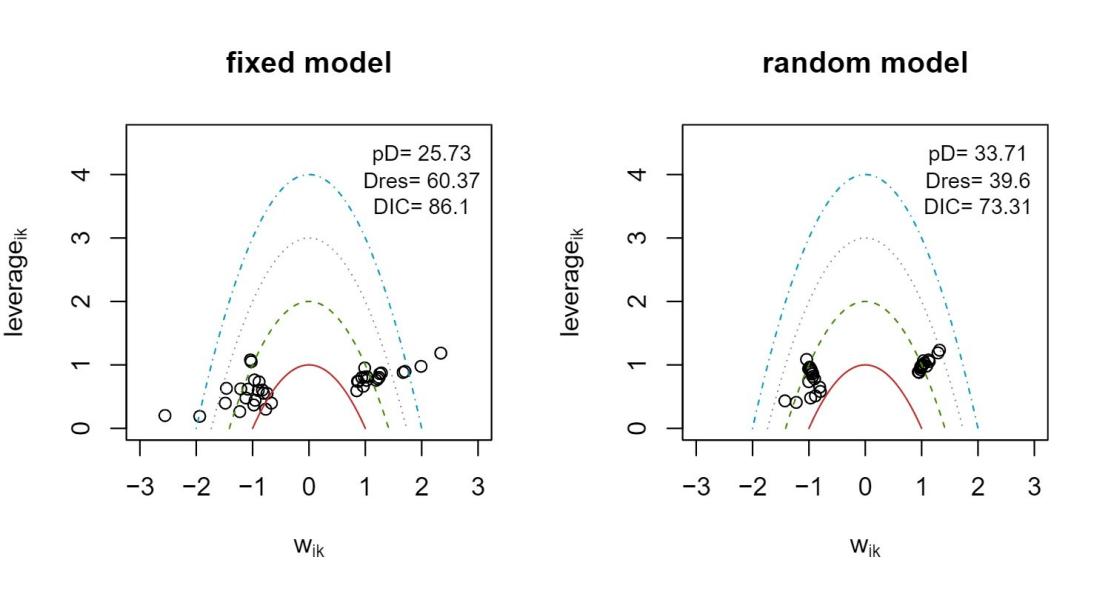

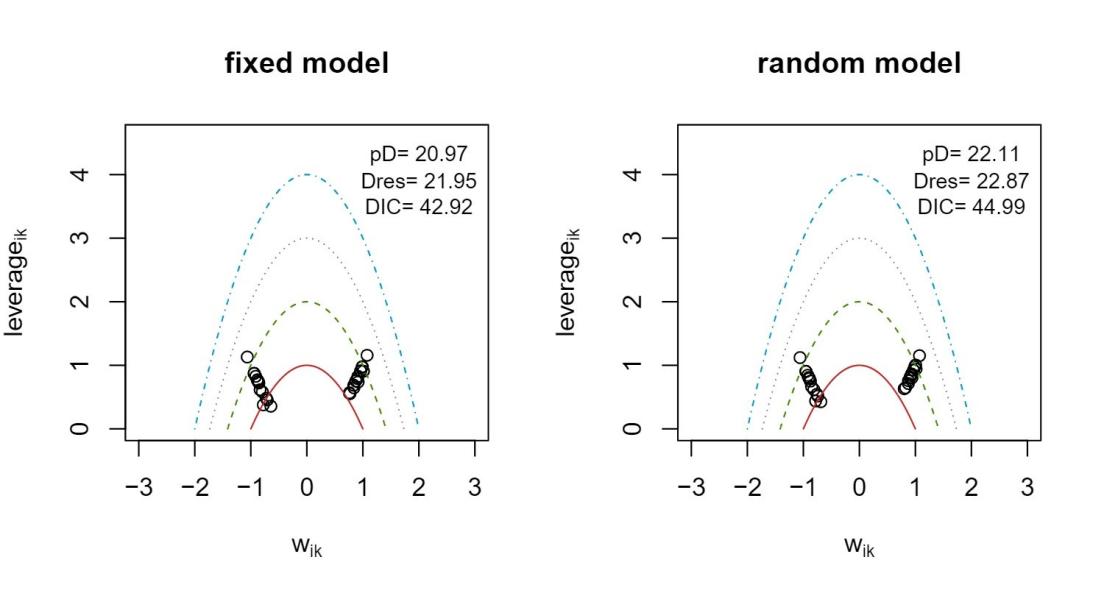


**c) Bleeding d) Adverse events**


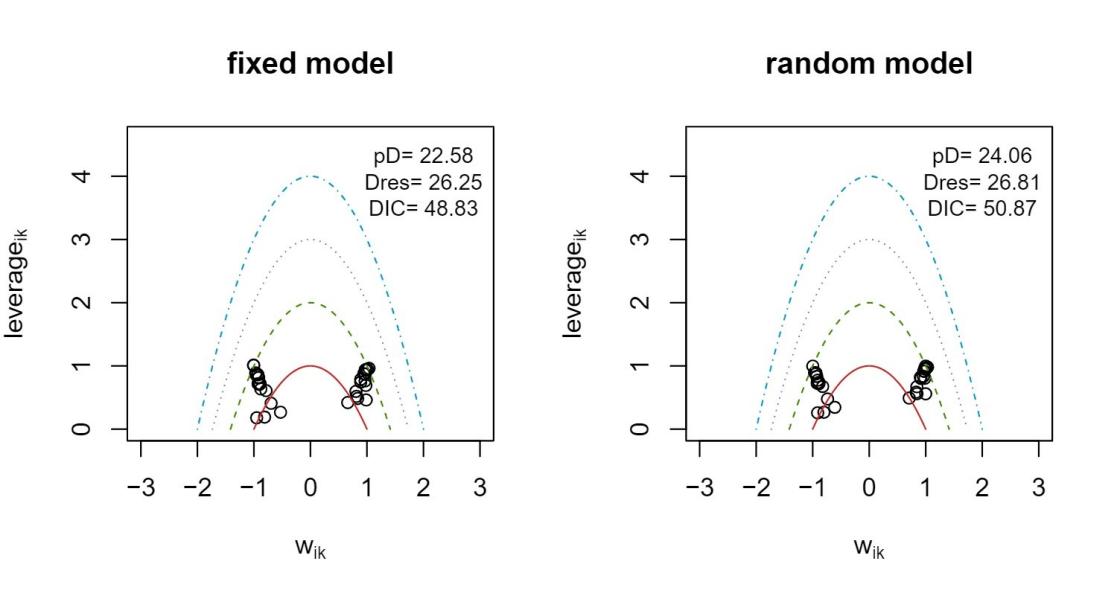

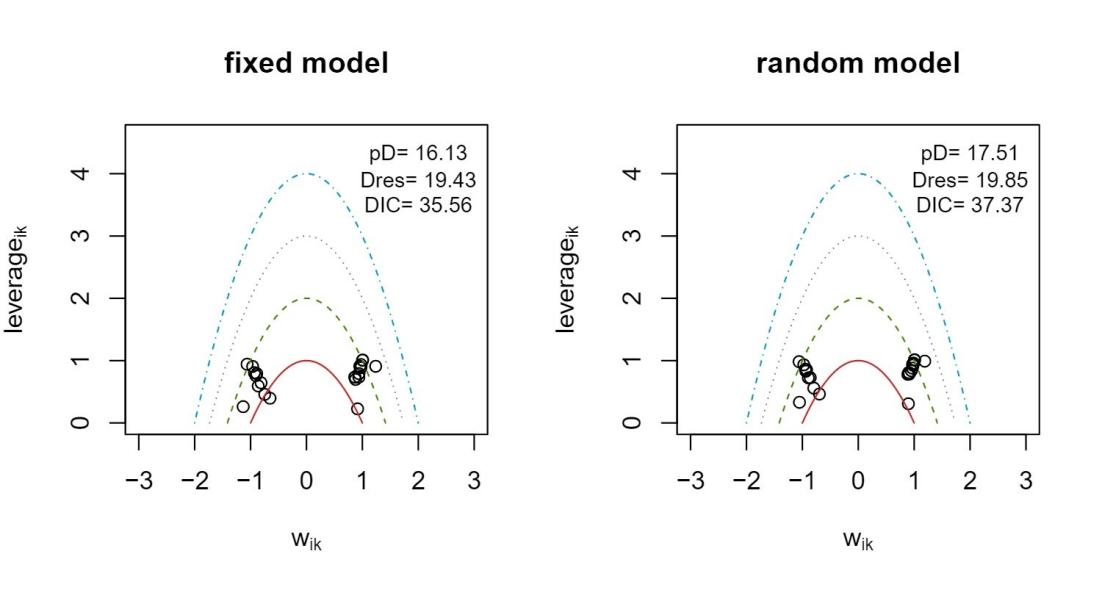


**Supplementary Figure 10.** Leverage plots of random model and fixed model

**Abbreviation:** DIC: deviance information criterion

**Note:** From the Figure we could see , the inconsistency model of venous thromboembolism has abnormal values (some data points are outside the purple arc). Combined with the DIC value, venous thromboembolism fit random model, and the other outcome indicators fit fixed model to estimate the pooling results

1. **Venous thromboembolism b) Major bleeding**


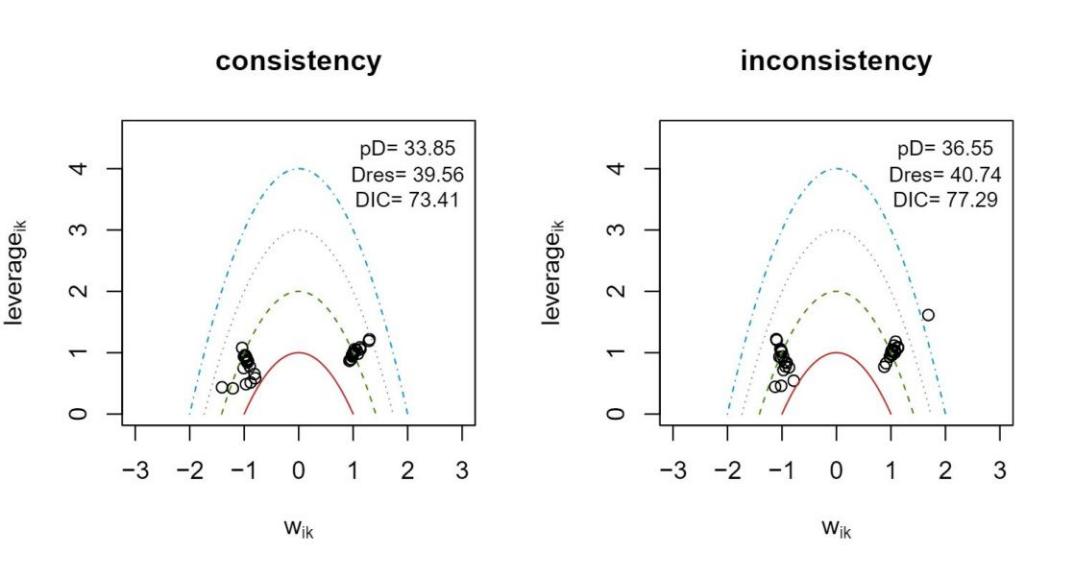

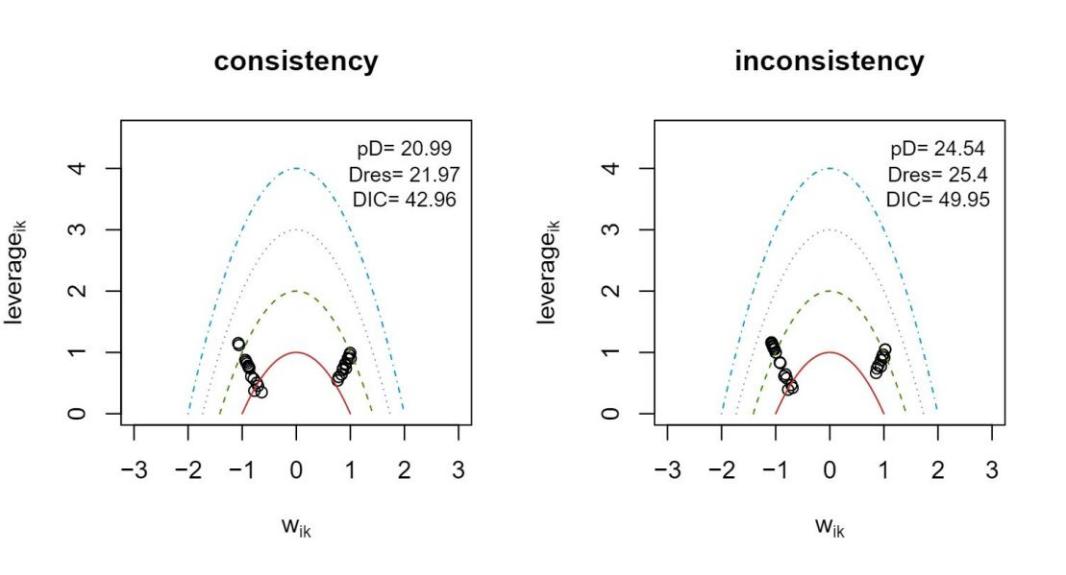


**c) Bleeding** **d) Adverse events**


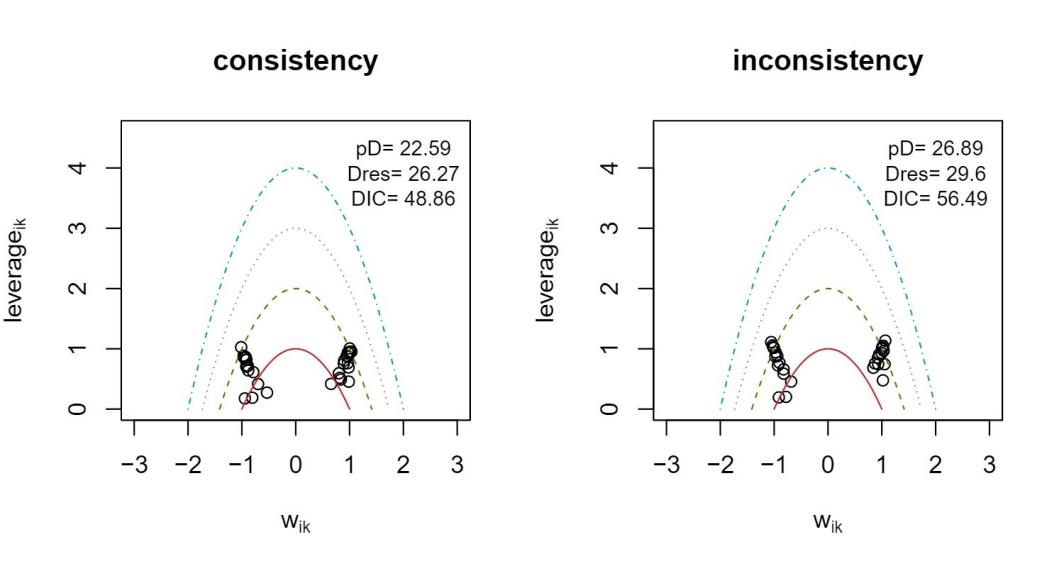

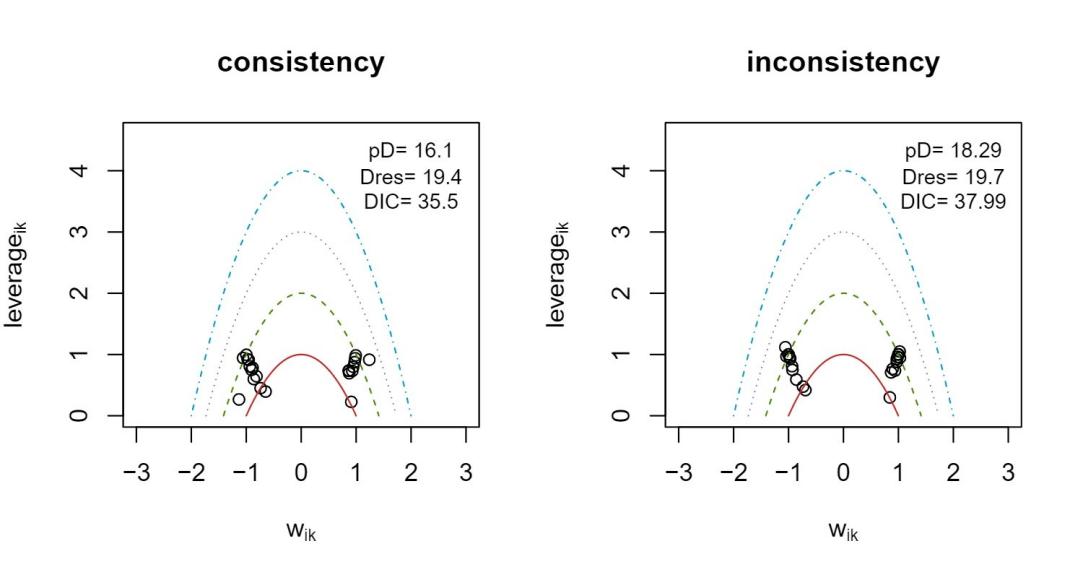


**Supplementary Figure 11.** Leverage plots of consistency model and inconsistency model

**Abbreviation:** DIC: deviance information criterion

**Note:** From the Figure we could see , the inconsistency model of venous thromboembolism has abnormal values (a data point is outside the purple arc). Combined with the DIC value, venous thromboembolism fit random model, and the other outcome indicators fit fixed model to estimate the pooling results


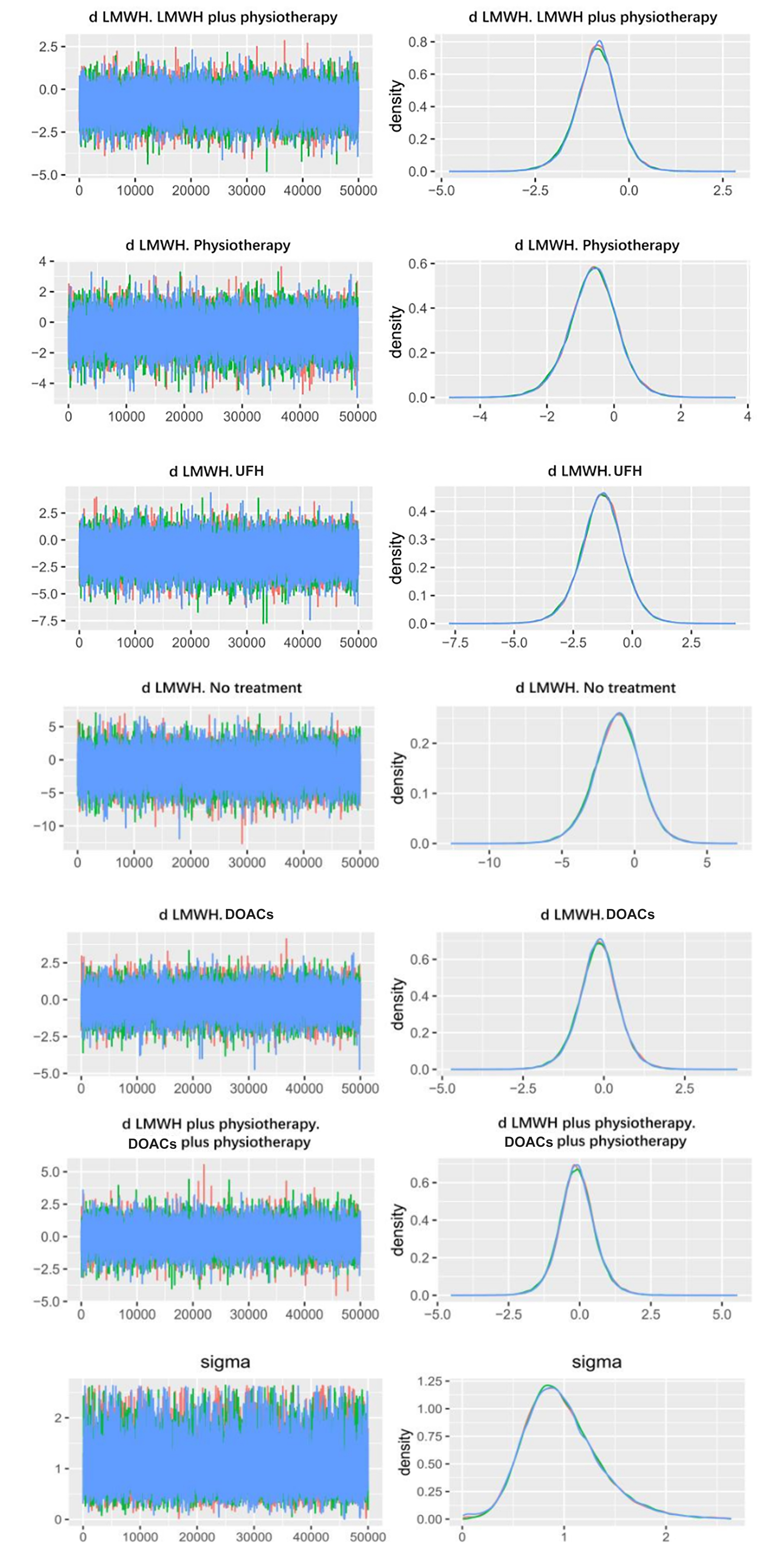


**Supplementary Figure 12.** Convergence: venous thromboembolism

**
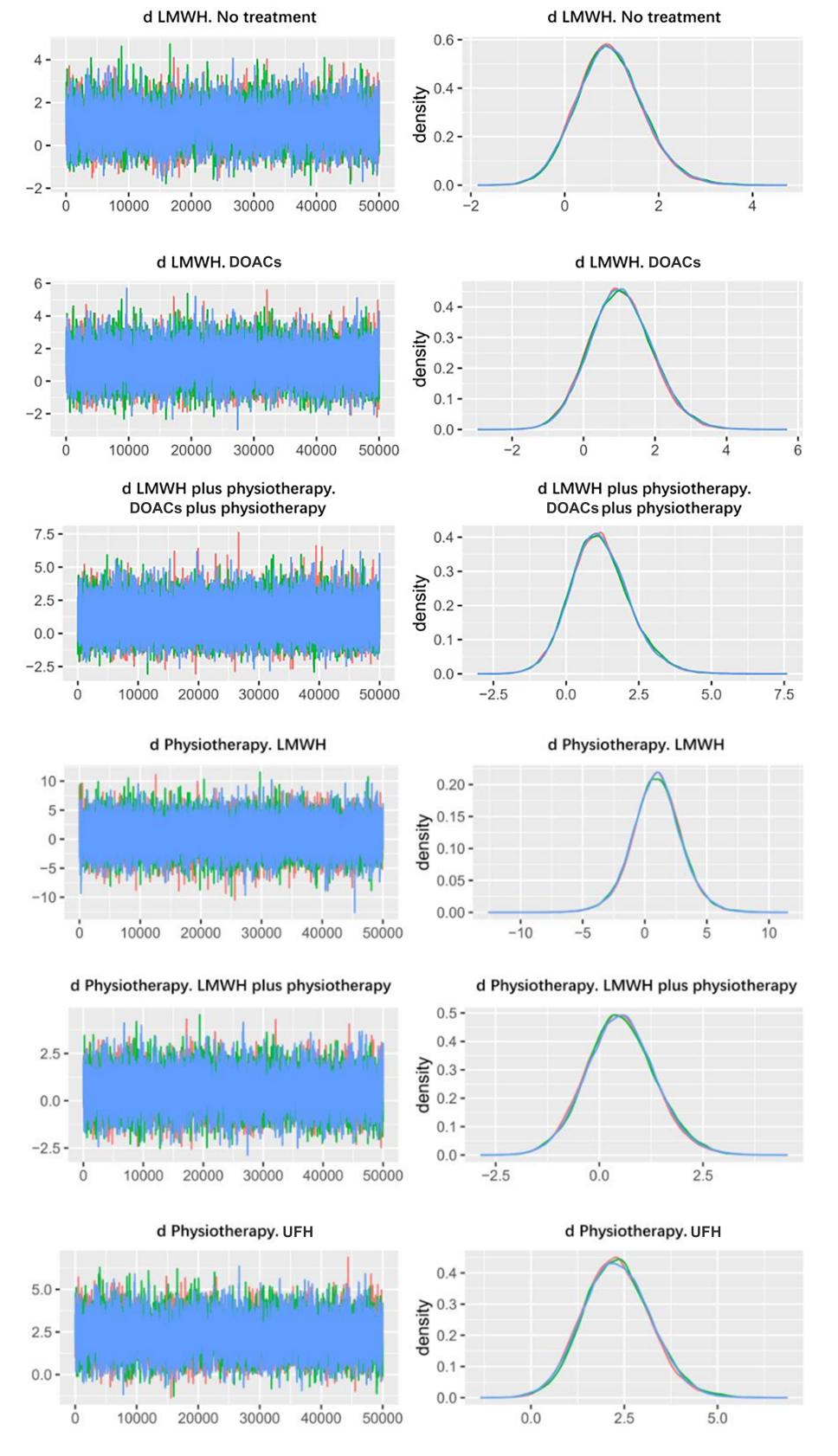
**

**Supplementary Figure 13.** Convergence: major bleeding

、


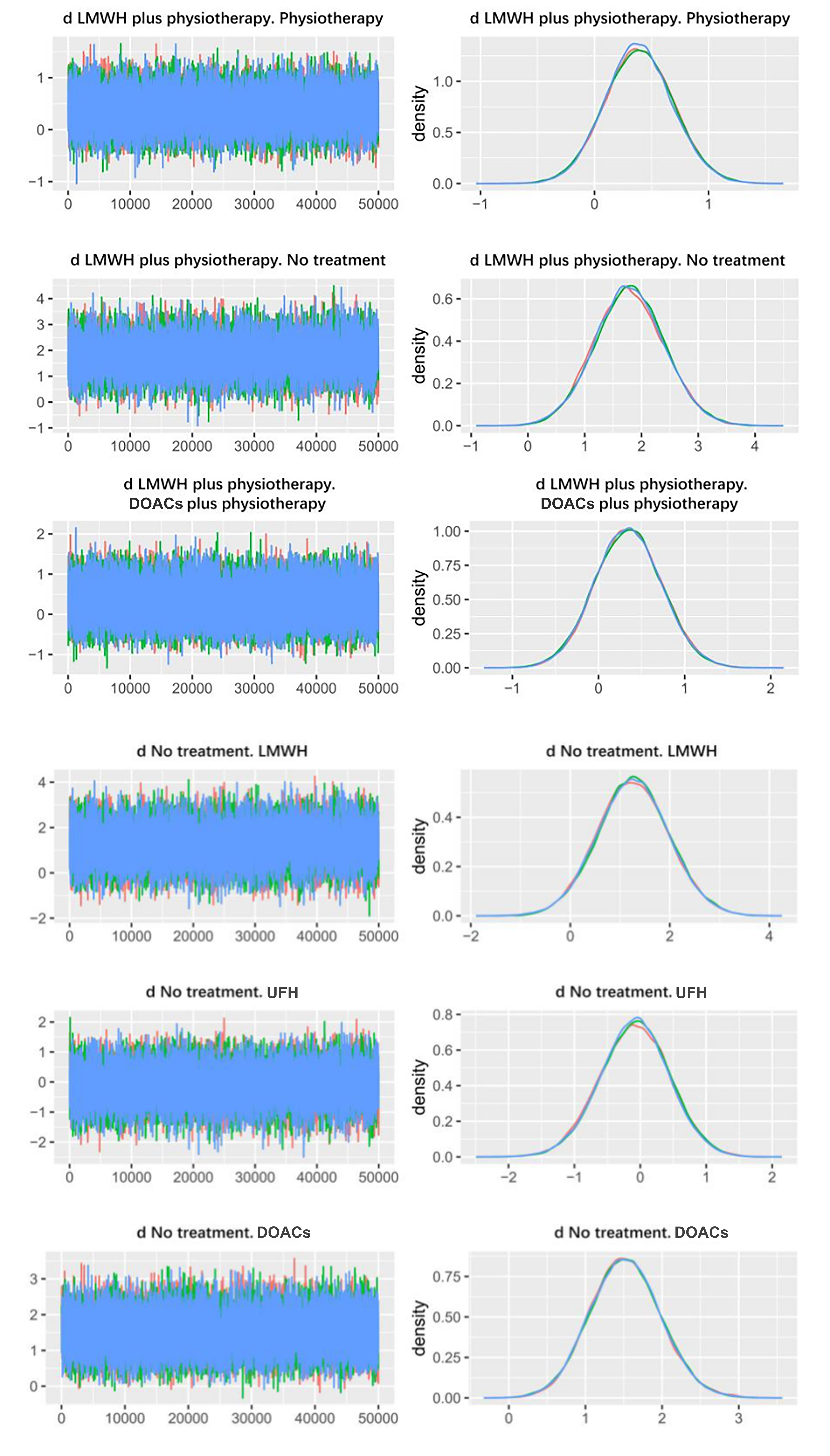


**Supplementary Figure 14.** Convergence: bleeding

、


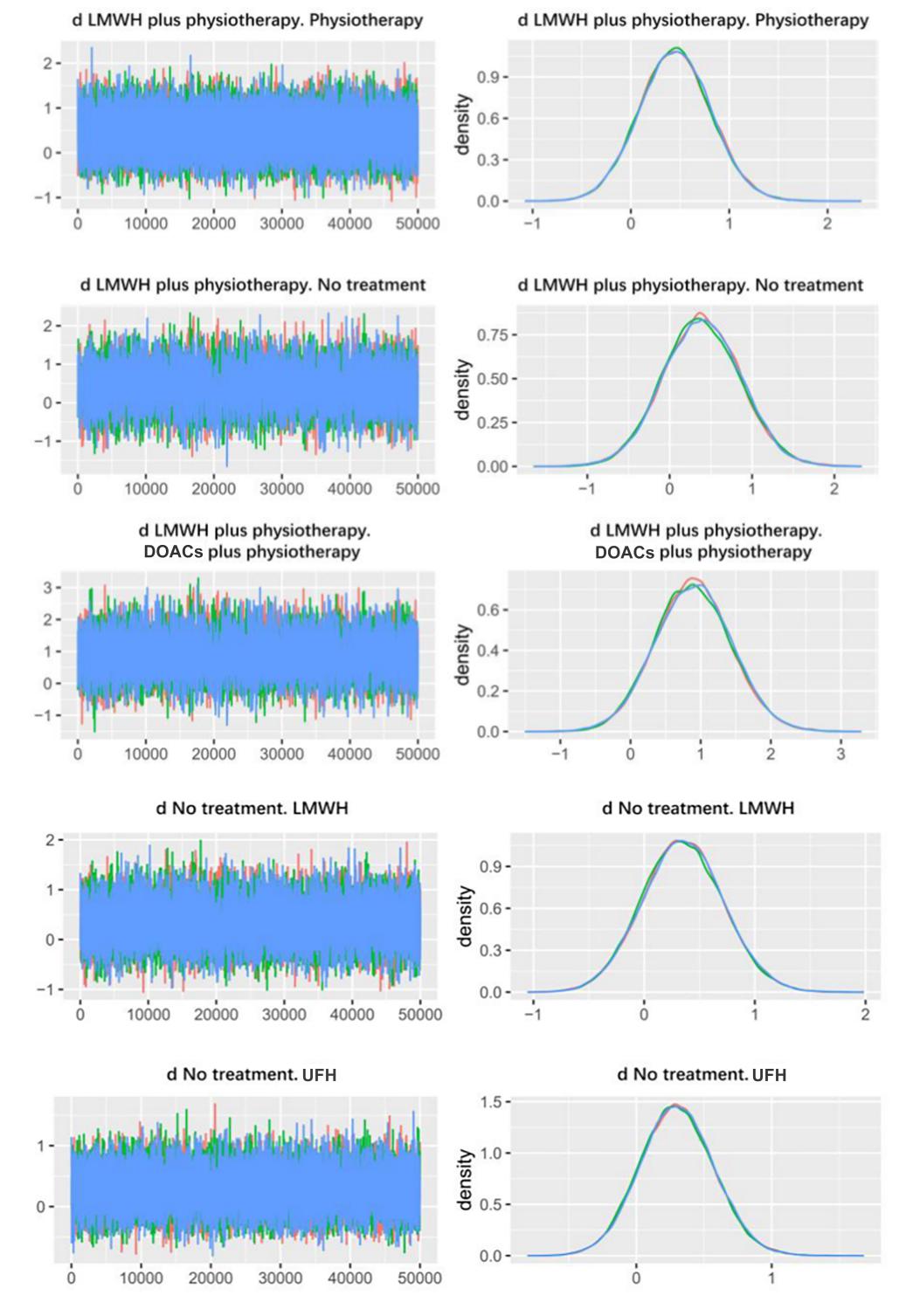


**Supplementary Figure 15.** Convergence: adverse events

**
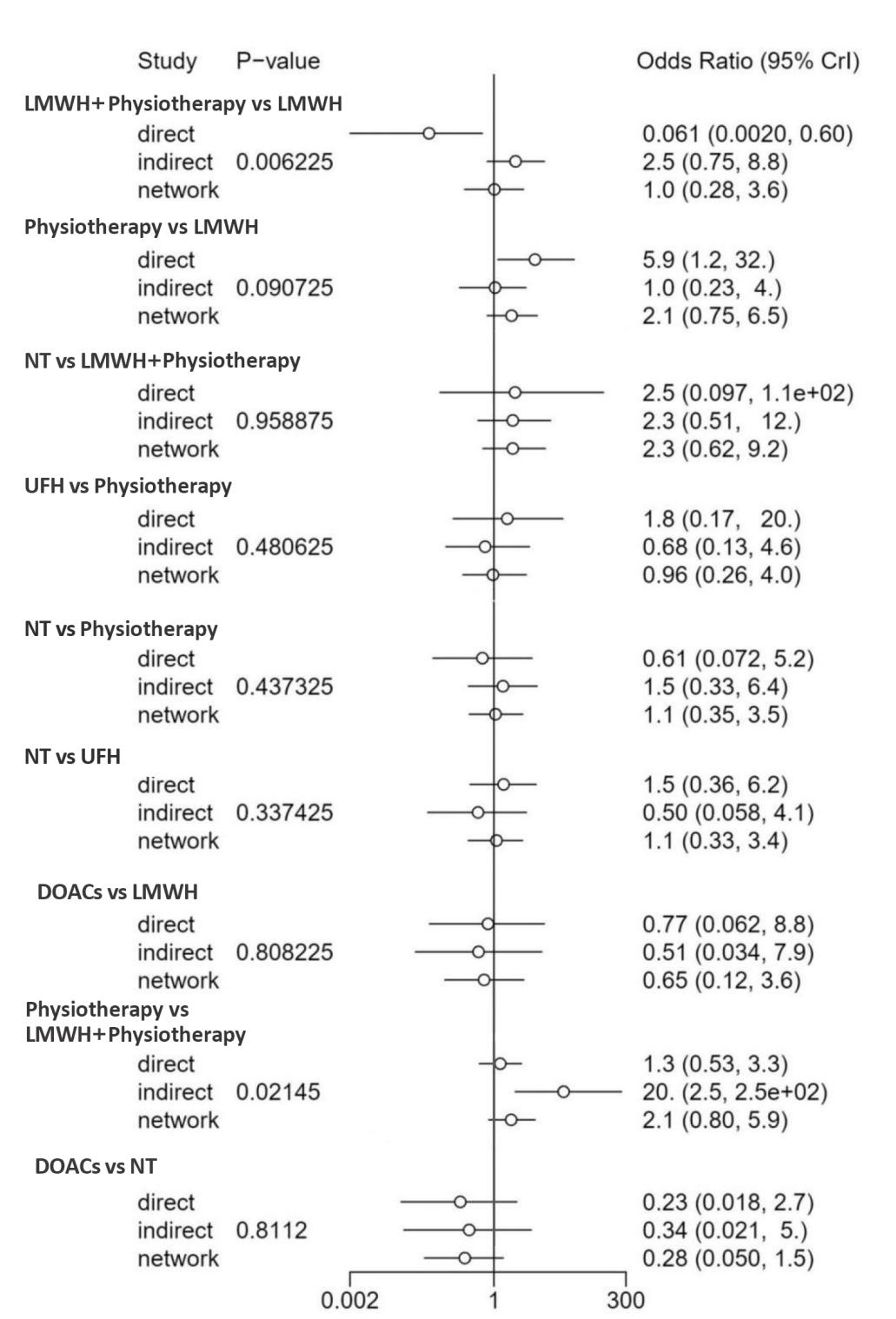
**

**Supplementary Figure 16.** Nodes-splitting analysis of venous thromboembolism

**Abbreviation:** LMWH: low molecular weight heparin; LMWH＋Physiotherapy: low molecular weight heparin (LMWH) plus physiotherapy; UFH: unfractionated heparin; NT: no treatment; DOACs: direct oral anticoagulants

**Note:** Analysis result shows evidence of statistical inconsistency, mainly in the direct LMWH plus physiotherapy vs LMWH comparison. Closer inspection reveals that the direct comparative odds ratio of Physiotherapy vs LMWH plus physiotherapy is less than that of Physiotherapy vs LMWH, which affect the results of the indirect comparison. Therefore, for inconsistencies, we choose to report direct comparison results. P-value ＜ 0.05 indicates that there is significant inconsistency

**
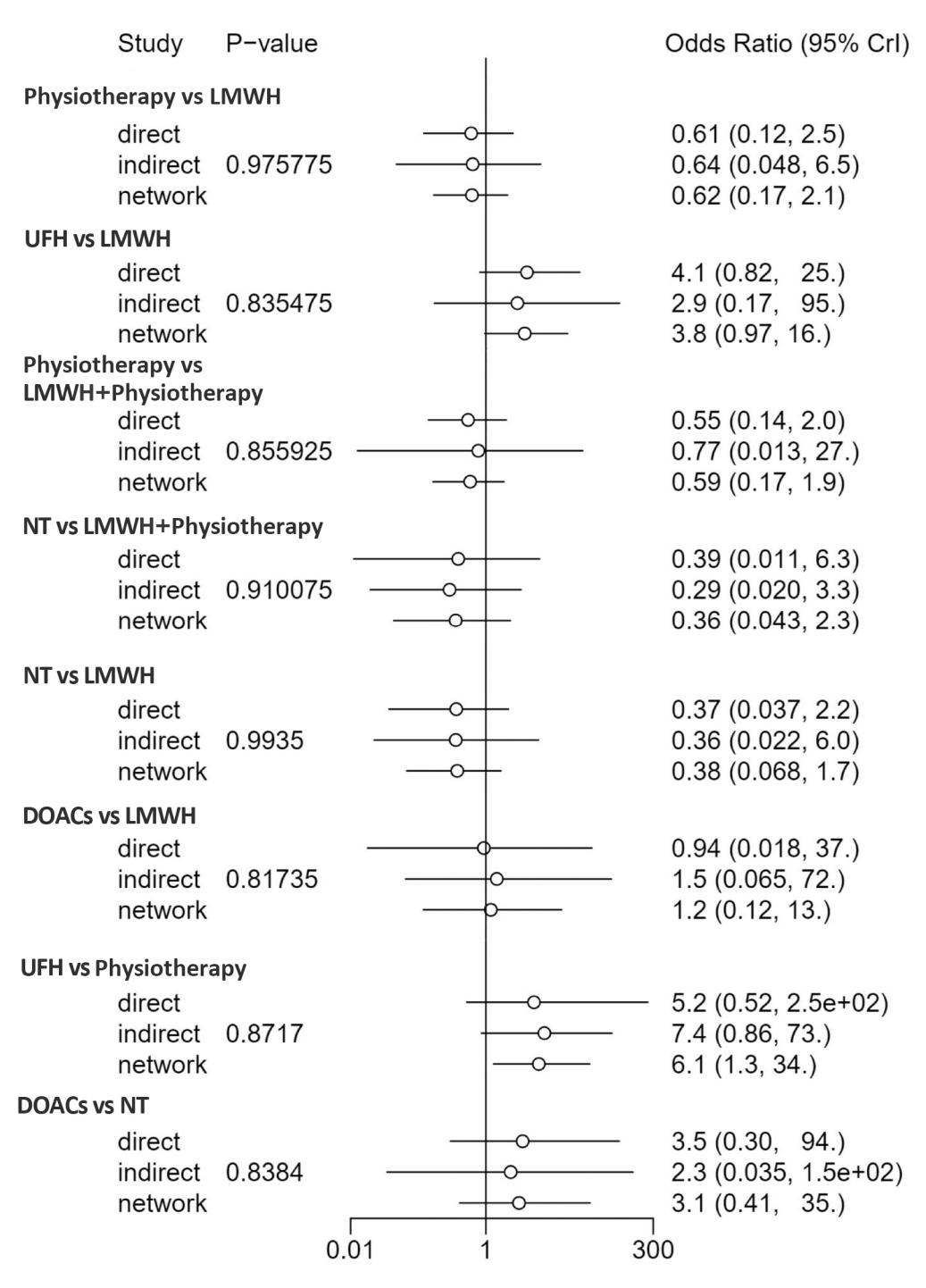
**

**Supplementary Figure** **17.** Nodes-splitting analysis of major bleeding

**Abbreviation:** LMWH: low molecular weight heparin; LMWH＋Physiotherapy: low molecular weight heparin (LMWH) plus physiotherapy; UFH: unfractionated heparin; NT: no treatment; DOACs: direct oral anticoagulants

**Note:** P-value > 0.05 indicates that there is no significant inconsistency which means the direct, indirect and network comparison is consistent


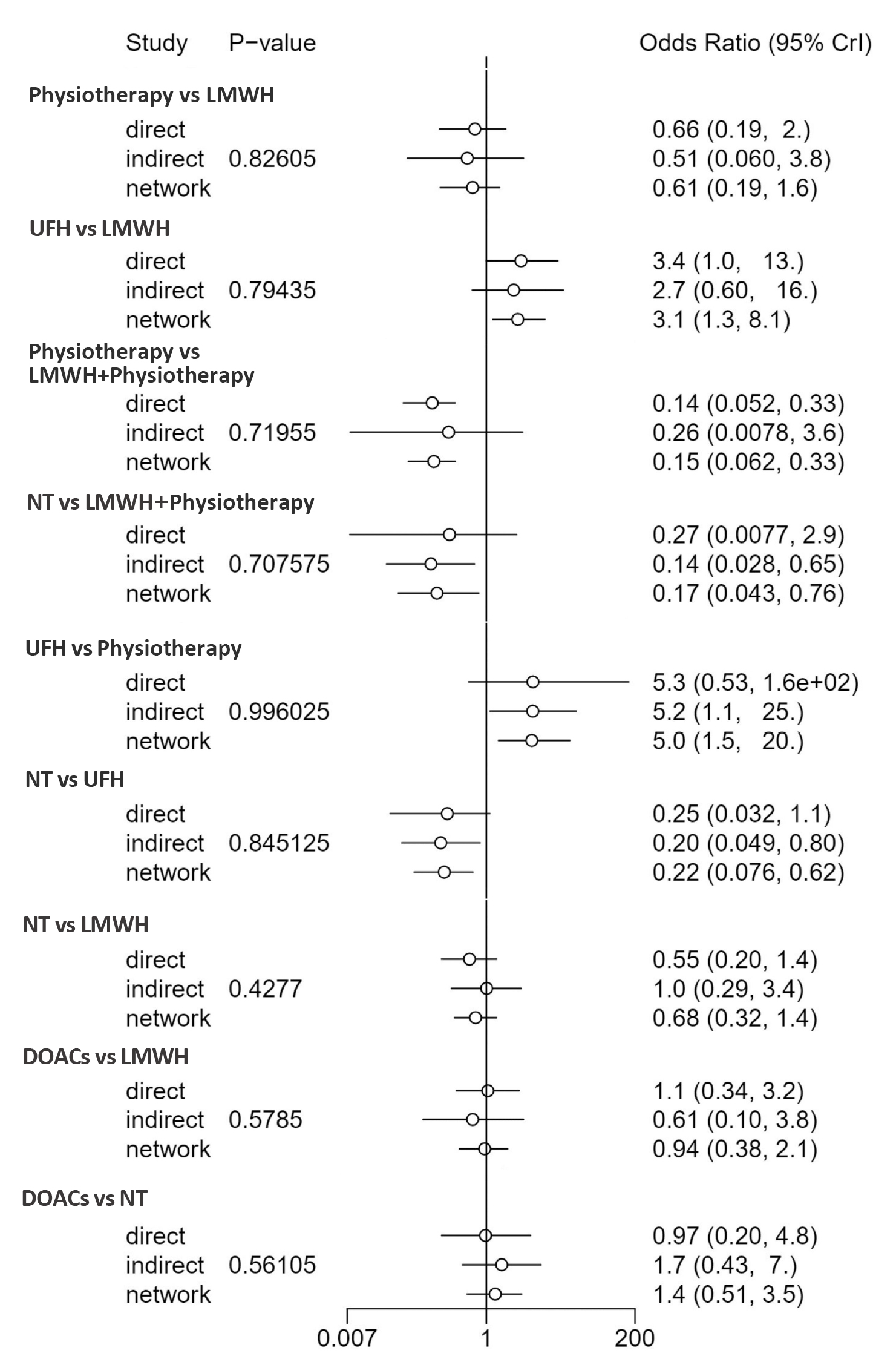


**Supplementary** **Figure** **18.** Nodes-splitting analysis of bleeding

**Abbreviation:** LMWH: low molecular weight heparin; LMWH＋Physiotherapy: low molecular weight heparin (LMWH) plus physiotherapy; UFH: unfractionated heparin; NT: no treatment; DOACs: direct oral anticoagulants

**Note:** P-value > 0.05 indicates that there is no significant inconsistency which means the direct, indirect and network comparison is consistent

**
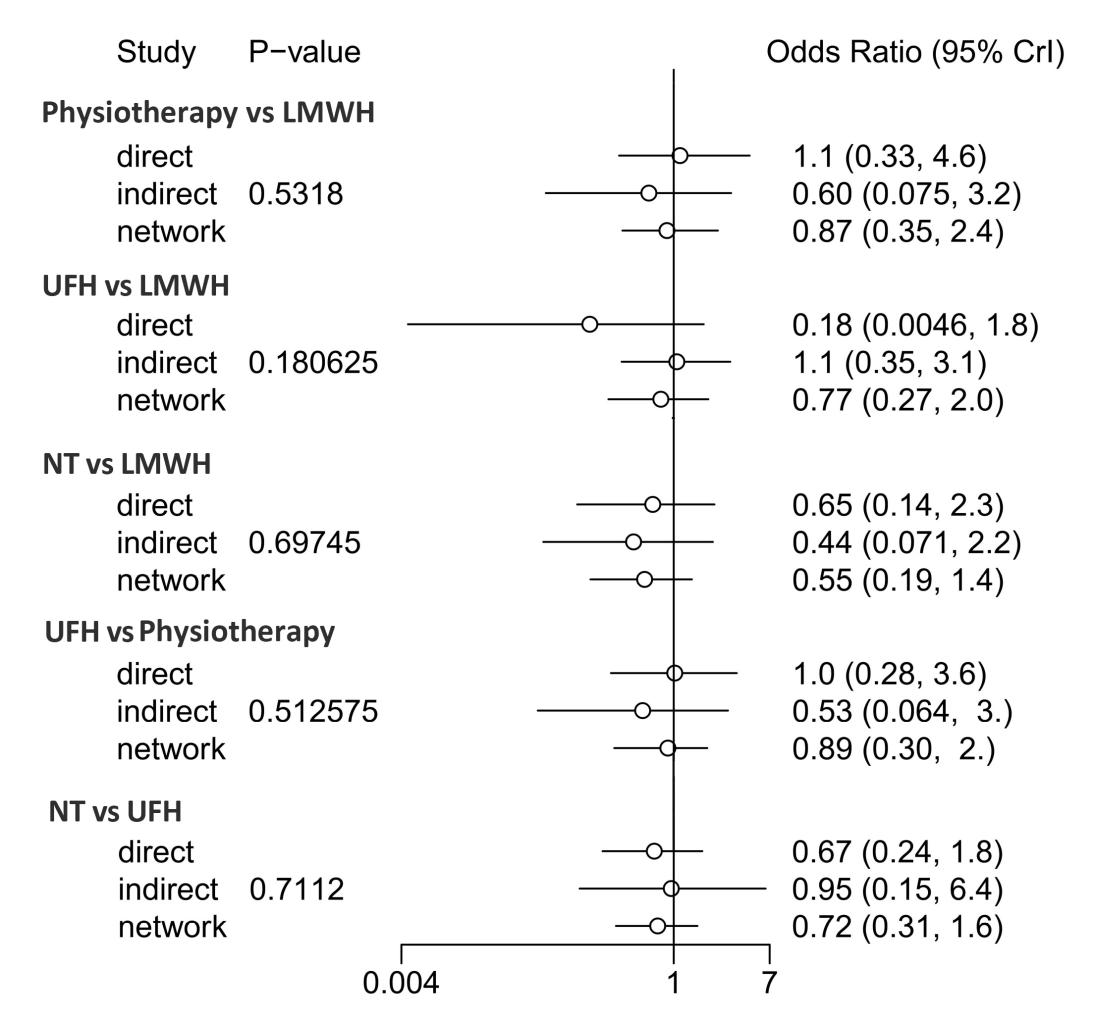
**

**Supplementary Figure** **19.** Nodes-splitting analysis of adverse events

**Abbreviation:** LMWH: low molecular weight heparin; LMWH＋Physiotherapy: low molecular weight heparin (LMWH) plus physiotherapy; UFH: unfractionated heparin; NT: no treatment; DOACs: direct oral anticoagulants

**Note:** P-value > 0.05 indicates that there is no significant inconsistency which means the direct, indirect and network comparison is consistent

**(A)** **Extended administration time**

a) Venous thromboembolism b) Major bleeding


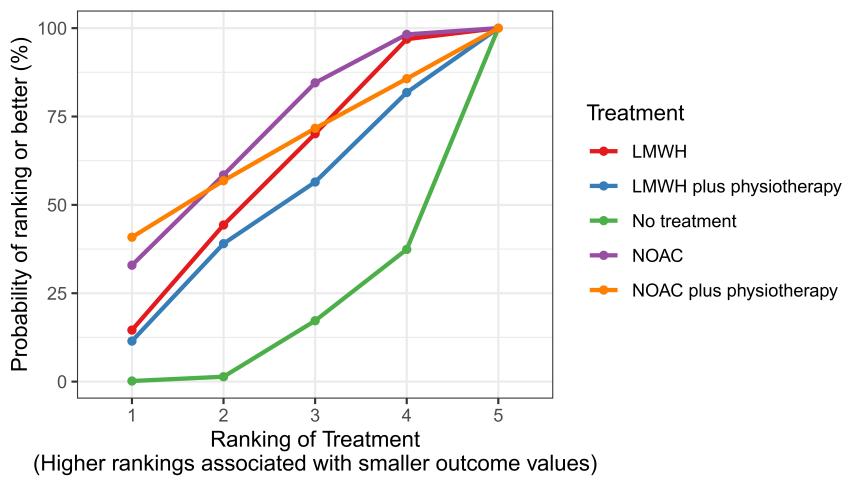

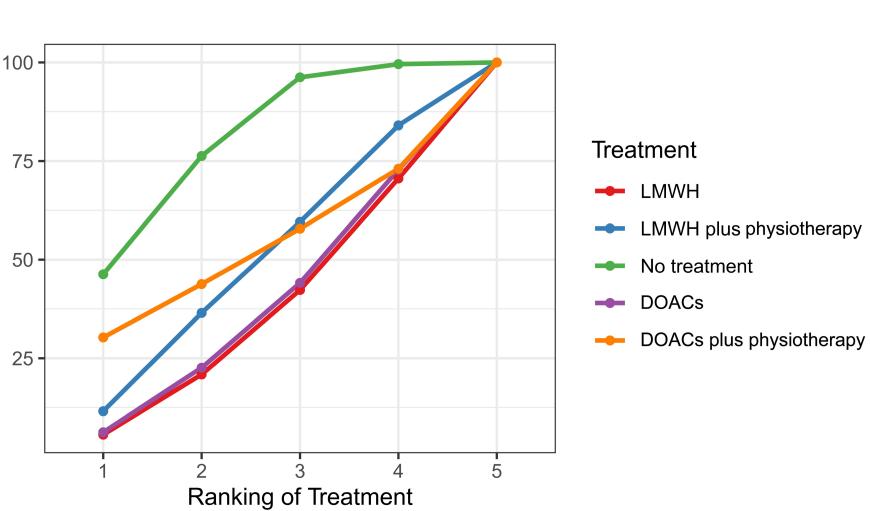


**(B) Non-extended administration time**

a) Venous thromboembolism b) Major bleeding


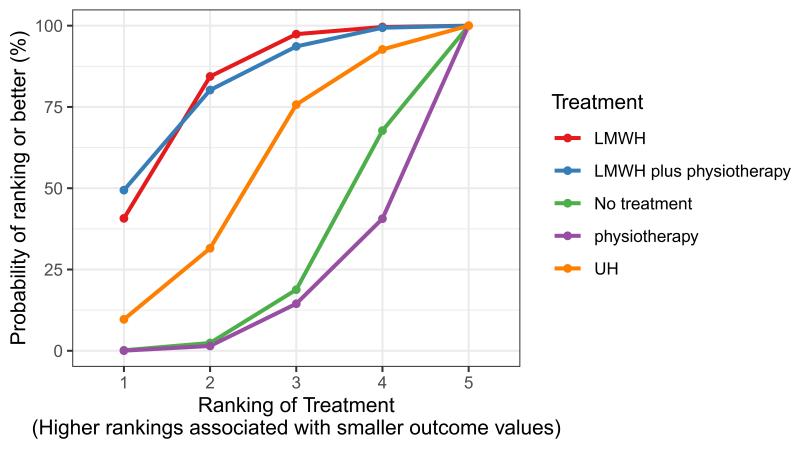

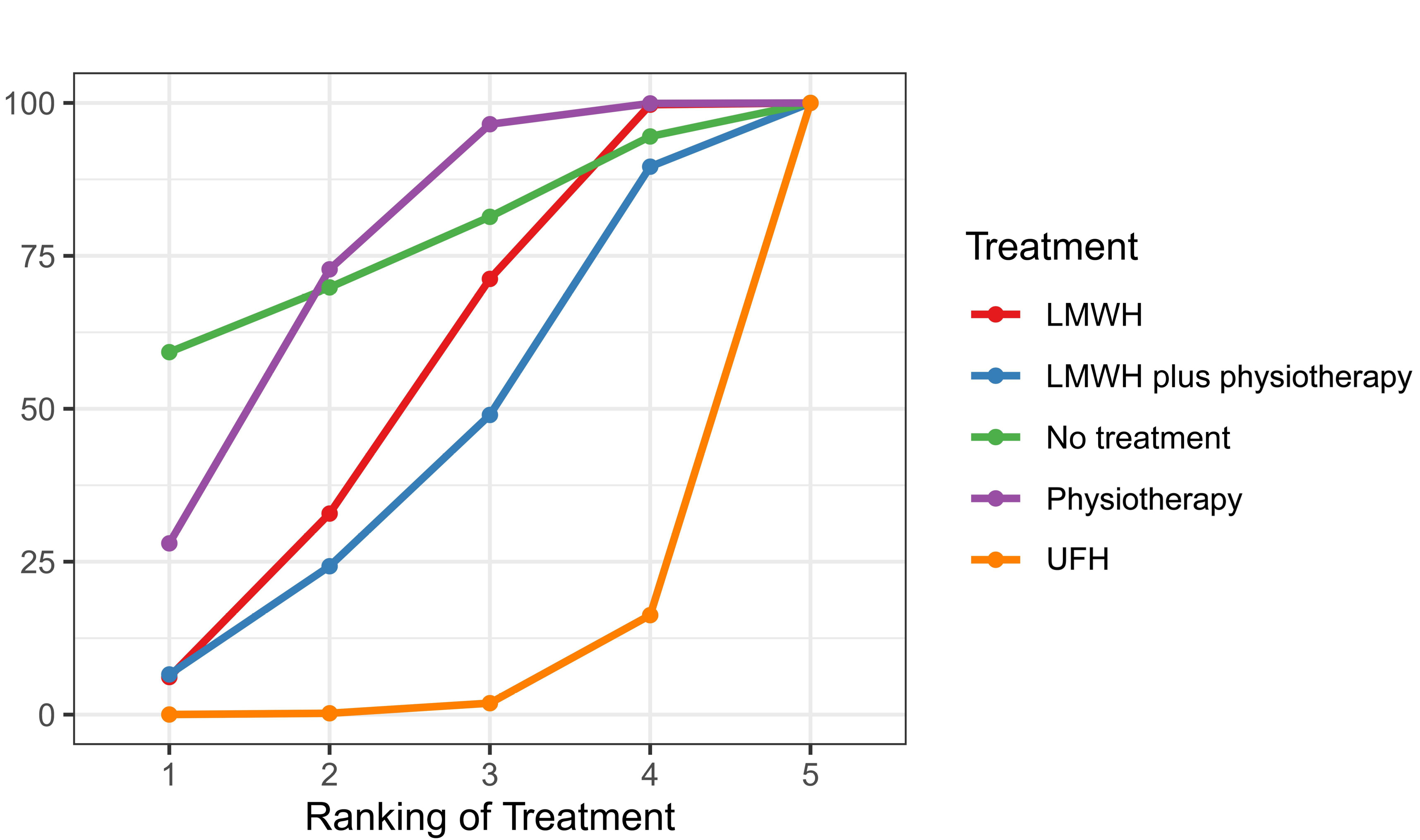


**Supplementary Figure** **20.** SUCRA plots of subgroup 1: duration of administration

**Abbreviation:** LMWH: low molecular weight heparin; UFH: unfractionated heparin; None: no treatment; DOACs: direct oral anticoagulants

**Note:** Due to our inclination to focus on which medication has a better effect on the primary outcome, we have chosen to present the SUCRA results. Extended administration time was defined as more than 4 weeks

**(A) Gyne**


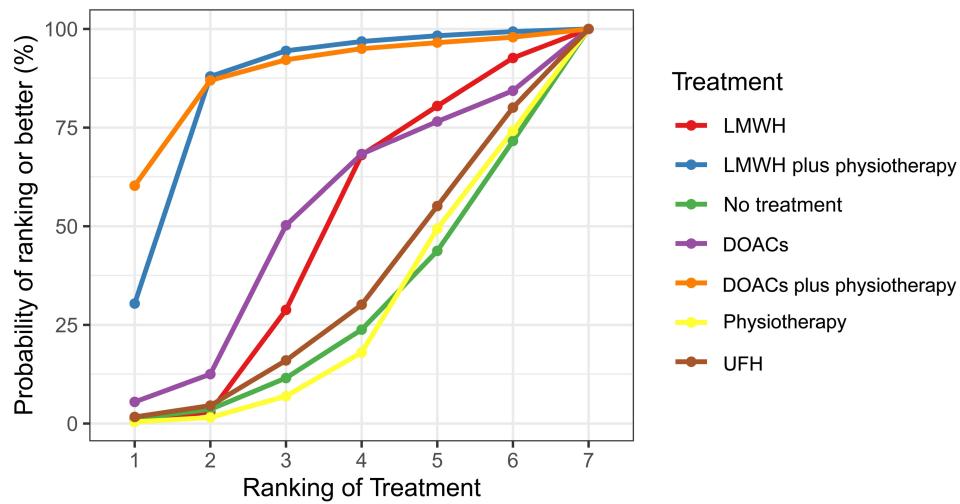


**(B) Multiple sites(GI/GU/Gyne)**


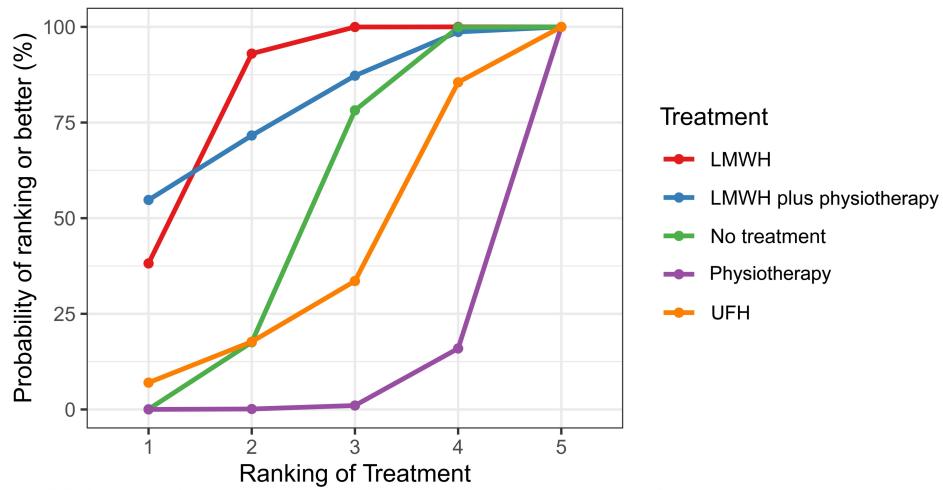


**(C) GI**


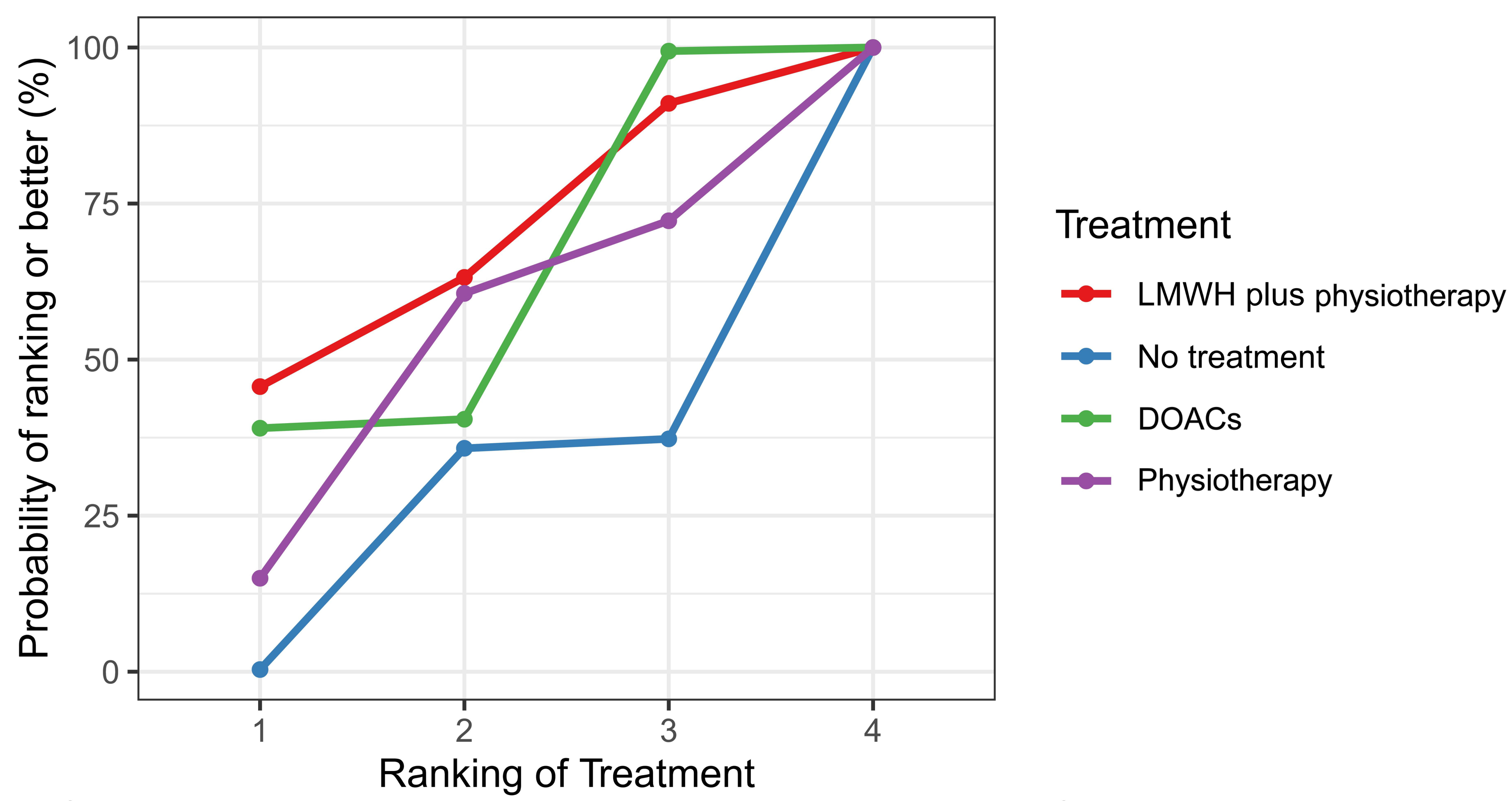


**Supplementary Figure 21.** SUCRA plots of subgroup 1: duration of administration

**Abbreviation:** LMWH: low molecular weight heparin; UFH: unfractionated heparin; GI: gastrointestinal; Gyne: gynecological; DOACs: direct oral anticoagulants; SUCRA: surface under the cumulative ranking curve;

**Note:** Due to the varying risks of cancer-related thrombosis in different anatomical sites, we focused solely on VTE outcomes and excluded GU studies from subgroup analysis due to insufficient research volume


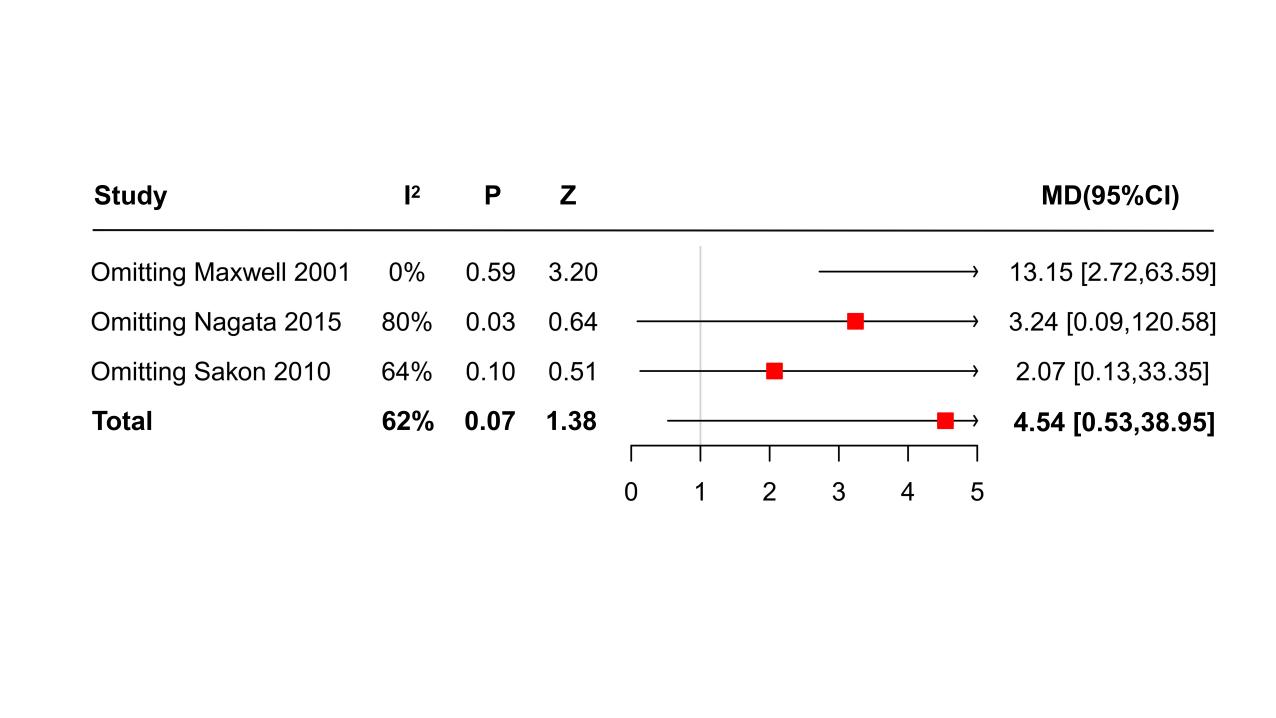


**Supplementary Figure 22.** Forest plot for sensitivity analysis

**Abbreviation:** MD (95%CI): Mean Difference (95% Confidence Interval)

**Note:** The graph presents the sensitivity analysis results comparing physical therapy with low molecular weight heparin. The Z-value represents the result of a Z-test on the population in question

**a) Venous thromboembolism b) Major bleeding**


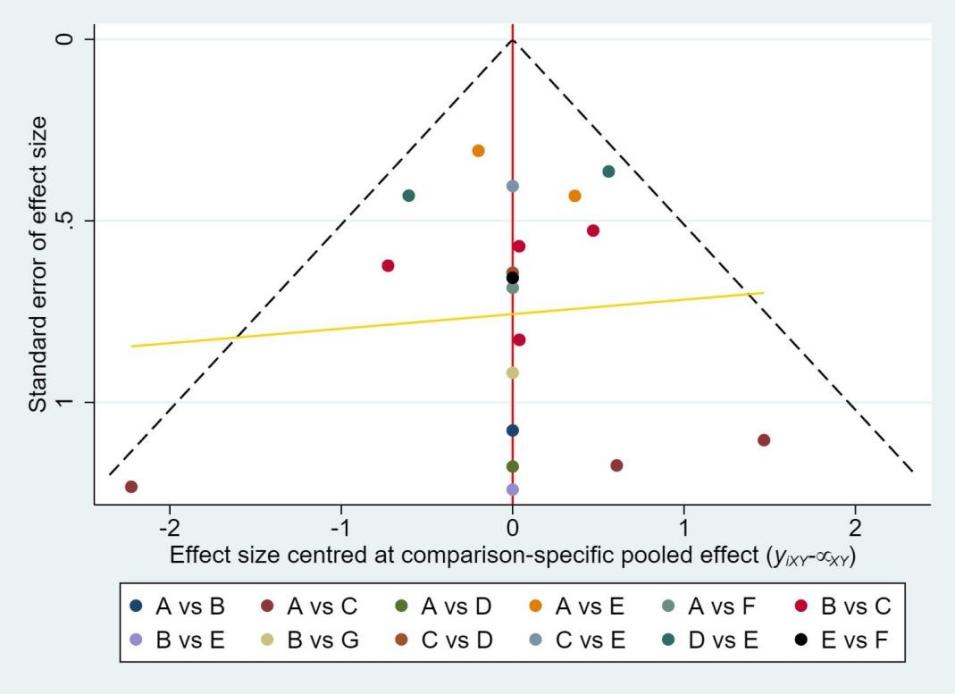

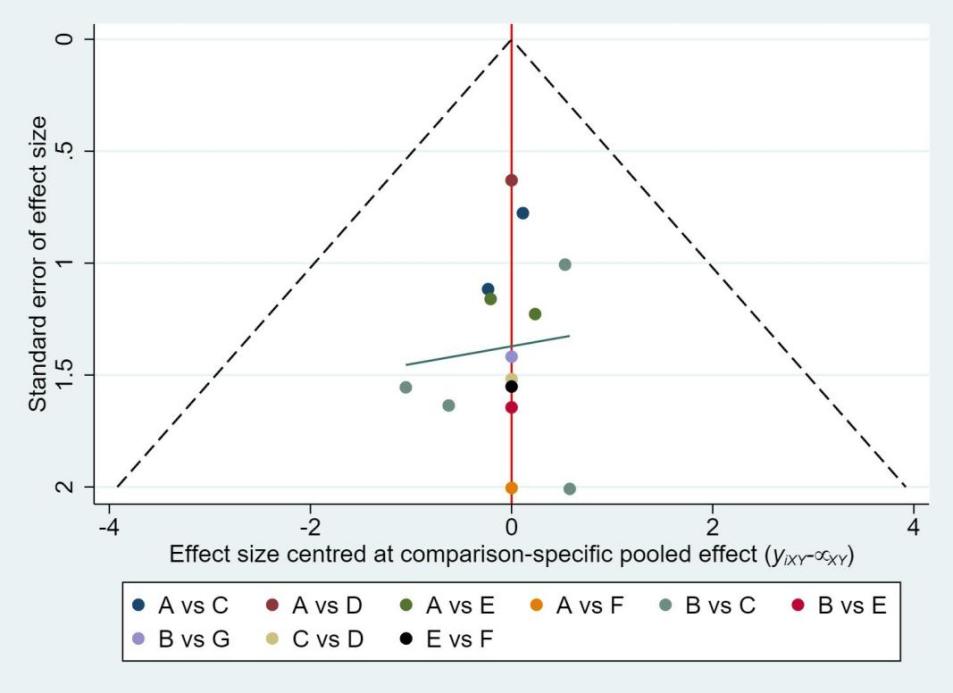


**Supplementary Figure 23**. Funnel plots of venous thromboembolism and major bleeding

**Abbreviation:** A: low molecular weight heparin (LMWH); B: low molecular weight heparin (LMWH) plus physiotherapy; C: Physiotherapy; D: unfractionated heparin (UFH); E: No treatment; F: Direct oral anticoagulants (DOACs); G: Direct oral anticoagulants (DOACs) plus physiotherapy.

**c) Bleeding d) Adverse events**


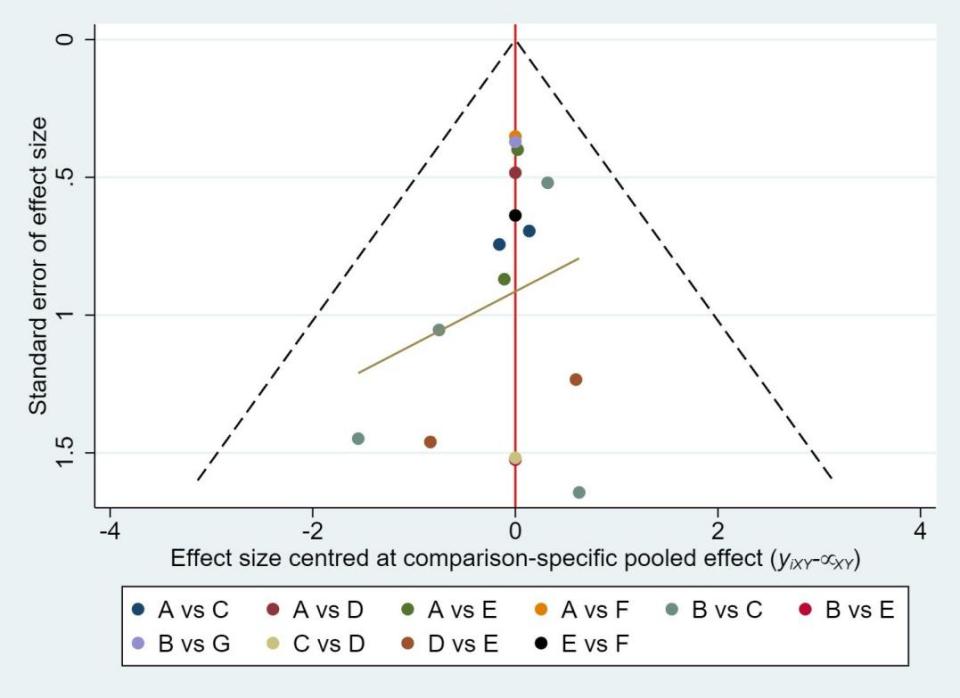

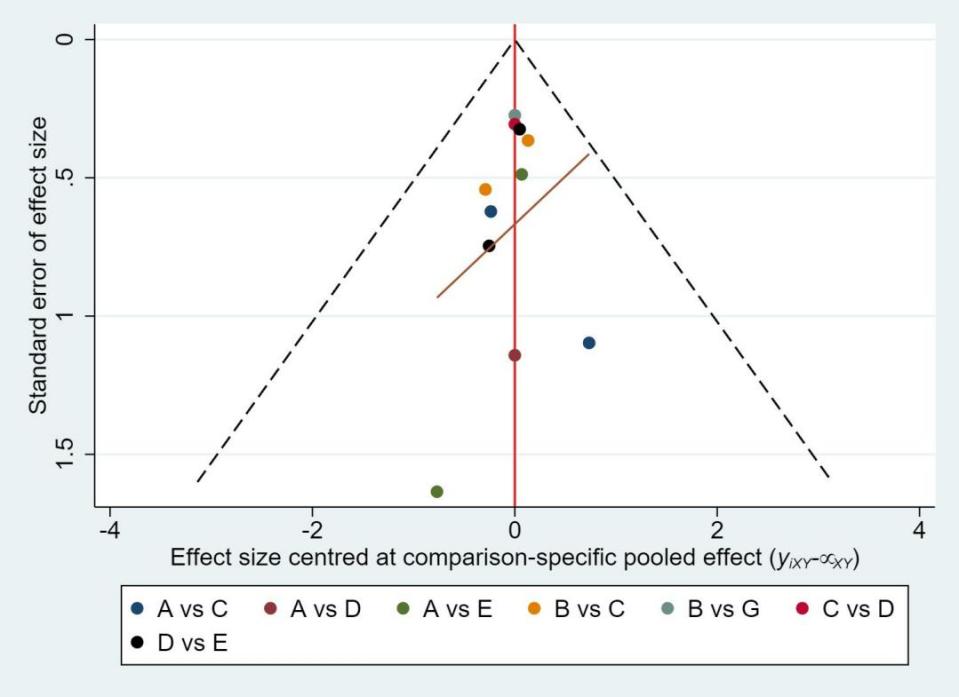


**Supplementary Figure 24**. Funnel plots of bleeding and adverse events

**Abbreviation:** A: low molecular weight heparin (LMWH); B: low molecular weight heparin (LMWH) plus physiotherapy; C: Physiotherapy; D: unfractionated heparin (UFH); E: No treatment; F: direct oral anticoagulants (DOACs); G: direct oral anticoagulants (DOACs) plus physiotherapy.
