# Supplementary material for: Comparison of efficacy and safety of thrombus prevention strategies after abdominal and pelvic cancer surgery: Bayesian network based meta-analysis
Source: Front Oncol. 2025 Feb 11;15:1445485. doi: 10.3389/fonc.2025.1445485 (PMC11851121; doi:10.3389/fonc.2025.1445485)
Supplement: Supplementary file 2 [file Table1.docx]

***Supplementary*** ***material***

Supplementary Table 1. PRISMA 2020 Checklist

Supplementary Table 2. Search Strategy

Supplementary Table 3. Outcome of included studies

Supplementary Table 4. Characteristics of included studies

**Supplementary Table 1.** PRISMA 2020 Checklist

| **Section and Topic** | **Item #** | **Checklist item** | **Location where item is reported** |
| --- | --- | --- | --- |
| **TITLE** | | |  |
| Title | 1 | Identify the report as a systematic review. | Page 1 |
| **ABSTRACT** | | |  |
| Abstract | 2 | See the PRISMA 2020 for Abstracts checklist. | Page 2 |
| **INTRODUCTION** | | |  |
| Rationale | 3 | Describe the rationale for the review in the context of existing knowledge. | Page 3 |
| Objectives | 4 | Provide an explicit statement of the objective(s) or question(s) the review addresses. | Page 3 |
| **METHODS** | | |  |
| Eligibility criteria | 5 | Specify the inclusion and exclusion criteria for the review and how studies were grouped for the syntheses. | Page 4 |
| Information sources | 6 | Specify all databases, registers, websites, organisations, reference lists and other sources searched or consulted to identify studies. Specify the date when each source was last searched or consulted. | Page 4 |
| Search strategy | 7 | Present the full search strategies for all databases, registers and websites, including any filters and limits used. | Supplementary materials |
| Selection process | 8 | Specify the methods used to decide whether a study met the inclusion criteria of the review, including how many reviewers screened each record and each report retrieved, whether they worked independently, and if applicable, details of automation tools used in the process. | Page 5 |
| Data collection process | 9 | Specify the methods used to collect data from reports, including how many reviewers collected data from each report, whether they worked independently, any processes for obtaining or confirming data from study investigators, and if applicable, details of automation tools used in the process. | Page 5 |
| Data items | 10a | List and define all outcomes for which data were sought. Specify whether all results that were compatible with each outcome domain in each study were sought (e.g. for all measures, time points, analyses), and if not, the methods used to decide which results to collect. | Page 4-5 |
|  | 10b | List and define all other variables for which data were sought (e.g. participant and intervention characteristics, funding sources). Describe any assumptions made about any missing or unclear information. | Page 4-5 |
| Study risk of bias assessment | 11 | Specify the methods used to assess risk of bias in the included studies, including details of the tool(s) used, how many reviewers assessed each study and whether they worked independently, and if applicable, details of automation tools used in the process. | Page 4 |
| Effect measures | 12 | Specify for each outcome the effect measure(s) (e.g. risk ratio, mean difference) used in the synthesis or presentation of results. | Page 4 |
| Synthesis methods | 13a | Describe the processes used to decide which studies were eligible for each synthesis (e.g. tabulating the study intervention characteristics and comparing against the planned groups for each synthesis (item #5)). | Page 3 |
|  | 13b | Describe any methods required to prepare the data for presentation or synthesis, such as handling of missing summary statistics, or data conversions. | Page 4-5 |
|  | 13c | Describe any methods used to tabulate or visually display results of individual studies and syntheses. | Page 4-5 |
|  | 13d | Describe any methods used to synthesize results and provide a rationale for the choice(s). If meta-analysis was performed, describe the model(s), method(s) to identify the presence and extent of statistical heterogeneity, and software package(s) used. | Page 4-5 |
|  | 13e | Describe any methods used to explore possible causes of heterogeneity among study results (e.g. subgroup analysis, meta-regression). | Page 4-5 |
|  | 13f | Describe any sensitivity analyses conducted to assess robustness of the synthesized results. | Page 4-5 |
| Reporting bias assessment | 14 | Describe any methods used to assess risk of bias due to missing results in a synthesis (arising from reporting biases). | Page 5 |
| Certainty assessment | 15 | Describe any methods used to assess certainty (or confidence) in the body of evidence for an outcome. | Page 6 |
| **RESULTS** | | |  |
| Study selection | 16a | Describe the results of the search and selection process, from the number of records identified in the search to the number of studies included in the review, ideally using a flow diagram. | Page 6-7 |
|  | 16b | Cite studies that might appear to meet the inclusion criteria, but which were excluded, and explain why they were excluded. | Page 6-7 |
| Study characteristics | 17 | Cite each included study and present its characteristics. | Page 6-7 |
| Risk of bias in studies | 18 | Present assessments of risk of bias for each included study. | Supplementary materials |
| Results of individual studies | 19 | For all outcomes, present, for each study: (a) summary statistics for each group (where appropriate) and (b) an effect estimate and its precision (e.g. confidence/credible interval), ideally using structured tables or plots. | Page 10-13 |
| Results of syntheses | 20a | For each synthesis, briefly summarise the characteristics and risk of bias among contributing studies. | Page 7-8 |
|  | 20b | Present results of all statistical syntheses conducted. If meta-analysis was done, present for each the summary estimate and its precision (e.g. confidence/credible interval) and measures of statistical heterogeneity. If comparing groups, describe the direction of the effect. | Page 5-6 |
|  | 20c | Present results of all investigations of possible causes of heterogeneity among study results. | Page 8 |
|  | 20d | Present results of all sensitivity analyses conducted to assess the robustness of the synthesized results. | Supplementary materials |
| Reporting biases | 21 | Present assessments of risk of bias due to missing results (arising from reporting biases) for each synthesis assessed. | Supplementary materials |
| Certainty of evidence | 22 | Present assessments of certainty (or confidence) in the body of evidence for each outcome assessed. | Supplementary materials |
| **DISCUSSION** | | |  |
| Discussion | 23a | Provide a general interpretation of the results in the context of other evidence. | Page 14 |
|  | 23b | Discuss any limitations of the evidence included in the review. | Page 15-16 |
|  | 23c | Discuss any limitations of the review processes used. | Page 15-16 |
|  | 23d | Discuss implications of the results for practice, policy, and future research. | Page 14-16 |
| **OTHER INFORMATION** | | |  |
| Registration and protocol | 24a | Provide registration information for the review, including register name and registration number, or state that the review was not registered. | Page 4 |
|  | 24b | Indicate where the review protocol can be accessed, or state that a protocol was not prepared. | Page 4 |
|  | 24c | Describe and explain any amendments to information provided at registration or in the protocol. | Not applicabile |
| Support | 25 | Describe sources of financial or non-financial support for the review, and the role of the funders or sponsors in the review. | Page 17 |
| Competing interests | 26 | Declare any competing interests of review authors. | Page 17 |
| Availability of data, code and other materials | 27 | Report which of the following are publicly available and where they can be found: template data collection forms; data extracted from included studies; data used for all analyses; analytic code; any other materials used in the review. | Supplementary materials |
| **Section and Topic** | **Item #** | **Checklist item** | **Location where item is reported** |
| **TITLE** | | |  |
| Title | 1 | Identify the report as a systematic review. | Page 1 |
| **ABSTRACT** | | |  |
| Abstract | 2 | See the PRISMA 2020 for Abstracts checklist. | Page 1-2 |
| **INTRODUCTION** | | |  |
| Rationale | 3 | Describe the rationale for the review in the context of existing knowledge. | Page 2 |
| Objectives | 4 | Provide an explicit statement of the objective(s) or question(s) the review addresses. | Page 2 |
| **METHODS** | | |  |
| Eligibility criteria | 5 | Specify the inclusion and exclusion criteria for the review and how studies were grouped for the syntheses. | Page 3 |
| Information sources | 6 | Specify all databases, registers, websites, organisations, reference lists and other sources searched or consulted to identify studies. Specify the date when each source was last searched or consulted. | Page 3 |
| Search strategy | 7 | Present the full search strategies for all databases, registers and websites, including any filters and limits used. | Supplementary materials |
| Selection process | 8 | Specify the methods used to decide whether a study met the inclusion criteria of the review, including how many reviewers screened each record and each report retrieved, whether they worked independently, and if applicable, details of automation tools used in the process. | Page 4 |
| Data collection process | 9 | Specify the methods used to collect data from reports, including how many reviewers collected data from each report, whether they worked independently, any processes for obtaining or confirming data from study investigators, and if applicable, details of automation tools used in the process. | Page 4 |
| Data items | 10a | List and define all outcomes for which data were sought. Specify whether all results that were compatible with each outcome domain in each study were sought (e.g. for all measures, time points, analyses), and if not, the methods used to decide which results to collect. | Page 3-4 |
|  | 10b | List and define all other variables for which data were sought (e.g. participant and intervention characteristics, funding sources). Describe any assumptions made about any missing or unclear information. | Page 3-4 |
| Study risk of bias assessment | 11 | Specify the methods used to assess risk of bias in the included studies, including details of the tool(s) used, how many reviewers assessed each study and whether they worked independently, and if applicable, details of automation tools used in the process. | Page 4 |
| Effect measures | 12 | Specify for each outcome the effect measure(s) (e.g. risk ratio, mean difference) used in the synthesis or presentation of results. | Page 4 |
| Synthesis methods | 13a | Describe the processes used to decide which studies were eligible for each synthesis (e.g. tabulating the study intervention characteristics and comparing against the planned groups for each synthesis (item #5)). | Page 3 |
|  | 13b | Describe any methods required to prepare the data for presentation or synthesis, such as handling of missing summary statistics, or data conversions. | Page 4-5 |
|  | 13c | Describe any methods used to tabulate or visually display results of individual studies and syntheses. | Page 4-5 |
|  | 13d | Describe any methods used to synthesize results and provide a rationale for the choice(s). If meta-analysis was performed, describe the model(s), method(s) to identify the presence and extent of statistical heterogeneity, and software package(s) used. | Page 4-5 |
|  | 13e | Describe any methods used to explore possible causes of heterogeneity among study results (e.g. subgroup analysis, meta-regression). | Page 4-5 |
|  | 13f | Describe any sensitivity analyses conducted to assess robustness of the synthesized results. | Page 4-5 |
| Reporting bias assessment | 14 | Describe any methods used to assess risk of bias due to missing results in a synthesis (arising from reporting biases). | Page 4 |
| Certainty assessment | 15 | Describe any methods used to assess certainty (or confidence) in the body of evidence for an outcome. | Page 5 |
| **RESULTS** | | |  |
| Study selection | 16a | Describe the results of the search and selection process, from the number of records identified in the search to the number of studies included in the review, ideally using a flow diagram. | Page 5-6 |
|  | 16b | Cite studies that might appear to meet the inclusion criteria, but which were excluded, and explain why they were excluded. | Page 5-6 |
| Study characteristics | 17 | Cite each included study and present its characteristics. | Page 5-6 |
| Risk of bias in studies | 18 | Present assessments of risk of bias for each included study. | Supplementary materials |
| Results of individual studies | 19 | For all outcomes, present, for each study: (a) summary statistics for each group (where appropriate) and (b) an effect estimate and its precision (e.g. confidence/credible interval), ideally using structured tables or plots. | Page 10-13 |
| Results of syntheses | 20a | For each synthesis, briefly summarise the characteristics and risk of bias among contributing studies. | Page 5 |
|  | 20b | Present results of all statistical syntheses conducted. If meta-analysis was done, present for each the summary estimate and its precision (e.g. confidence/credible interval) and measures of statistical heterogeneity. If comparing groups, describe the direction of the effect. | Page 4-5 |
|  | 20c | Present results of all investigations of possible causes of heterogeneity among study results. | Page 7 |
|  | 20d | Present results of all sensitivity analyses conducted to assess the robustness of the synthesized results. | Supplementary materials  Page 25 |
| Reporting biases | 21 | Present assessments of risk of bias due to missing results (arising from reporting biases) for each synthesis assessed. | Supplementary materials  Page 3-6 |
| Certainty of evidence | 22 | Present assessments of certainty (or confidence) in the body of evidence for each outcome assessed. | Supplementary materials  Page 11-20 |
| **DISCUSSION** | | |  |
| Discussion | 23a | Provide a general interpretation of the results in the context of other evidence. | Page 14 |
|  | 23b | Discuss any limitations of the evidence included in the review. | Page 15-16 |
|  | 23c | Discuss any limitations of the review processes used. | Page 15-16 |
|  | 23d | Discuss implications of the results for practice, policy, and future research. | Page 14-16 |
| **OTHER INFORMATION** | | |  |
| Registration and protocol | 24a | Provide registration information for the review, including register name and registration number, or state that the review was not registered. | Page 3 |
|  | 24b | Indicate where the review protocol can be accessed, or state that a protocol was not prepared. | Page 3 |
|  | 24c | Describe and explain any amendments to information provided at registration or in the protocol. | Not applicabile |
| Support | 25 | Describe sources of financial or non-financial support for the review, and the role of the funders or sponsors in the review. | Page 10 |
| Competing interests | 26 | Declare any competing interests of review authors. | Page 10 |
| Availability of data, code and other materials | 27 | Report which of the following are publicly available and where they can be found: template data collection forms; data extracted from included studies; data used for all analyses; analytic code; any other materials used in the review. | Page |

**Supplementary Table 2.** Search Strategy

| **PubMed** **(January 12, 2024)** | | |
| --- | --- | --- |
| **#1** | Neoplasm[MeSH] OR carcinoma[MeSH] OR sarcoma[MeSH]OR Neoplasms[tiab] OR Neoplasm[tiab] OR Tumor[tiab] OR Neoplasm[tiab] OR Neoplasia[tiab] OR Cancer[tiab] OR Malignant[tiab] OR Malignancy[tiab] OR Malignancies[tiab] OR Benign Neoplasm[tiab] OR oncoma[tiab] OR carcinoma[tiab] OR ade-nocarcinoma[tiab] OR tumour[tiab] OR oncology[tiab] OR arcin*[tiab] OR cancer*[tiab] OR neoplas*[tiab] OR tumour*[tiab] OR tumor*[tiab] OR cyst*[tiab] OR growth*[tiab] OR adenocarcin*[tiab] OR malignant[tiab] OR malig*[tiab] OR sarcoma[tiab] OR glioblastoma[MeSH] OR glioma[MeSH] OR melanoma[MeSH] OR osteosarcoma[MeSH] OR hepatoblastoma[MeSH] OR Lymphoma[MeSH] OR chondrosarcoma[MeSH] OR meningioma[MeSH] OR choriocarcinoma[MeSH] OR fibrosarcoma[MeSH] OR liposarcoma[MeSH] OR Hemangiosarcoma[MeSH] OR mesothelioma[MeSH] OR leiomyosarcoma[MeSH] OR rhabdomyosarcoma[MeSH] OR cystadenocarcinoma[MeSH] OR seminoma[MeSH] OR glioblastoma[tiab] OR glioblastomas[tiab] OR glioma[tiab] OR Gliomas[tiab] OR melanoma[tiab] OR osteosarcoma[tiab] OR hepatoblastoma[tiab] OR Lymphoma[tiab] OR chondrosarcoma[tiab] OR meningioma[tiab] OR choriocarcinoma[tiab] OR fibrosarcoma[tiab] OR liposarcoma[tiab] OR Hemangiosarcoma[tiab] OR Angiosarcoma[tiab] OR mesothelioma[tiab] OR leiomyosarcoma[tiab] OR rhabdomyosarcoma[tiab] OR cystadenocarcinoma[tiab] OR seminoma[tiab] | 6720180 |
| **#2** | apixaban [Supplementary Concept] OR edoxaban [Supplementary Concept] OR Warfarin[MeSH] OR dalteparin[MeSH] OR Heparin, Low-Molecular-Weight[MeSH] OR Fondaparinux[MeSH] OR Heparin[Mesh] OR Nadroparin[MeSH] OR tinzaparin[MeSH] OR Enoxaparin[Mesh] OR dalteparin[tiab] OR Tedelparin[tiab] OR Dalteparin Sodium[tiab] OR Fragmin[tiab] OR Fragmine[tiab] OR Low molecular weight heparin[tiab] OR LMWH[tiab] OR Heparin[tiab] OR Unfractionated Heparin[tiab] OR Heparinic Acid[tiab] OR Liquaemin[tiab] OR Sodium Heparin[tiab] OR Heparin Sodium[tiab] OR alpha Heparinheparin [tiab] OR Oral Factor Xa Inhibitor[tiab] OR Fondaparinux[tiab] OR Fondaparinux Sodium[tiab] OR Quixidar[tiab] OR Arixtra[tiab] OR Arixtra[tiab] OR Nadroparin[tiab] OR Nadroparine[tiab] OR Nadroparin Calcium[tiab] OR Fraxiparine OR Enoxaparin[tiab] OR fraxiparin[tiab] OR clexane[tiab] OR lovenox [tiab] OR fragmin[tiab] OR ardeparin[tiab] OR normiﬂo[tiab] OR tinzaparin[tiab] OR logiparin[tiab] OR innohep[tiab] OR certoparin[tiab] OR sandoparin[tiab] OR reviparin[tiab] OR clivarin[tiab] OR danaproid[tiab] OR orgaran[tiab] OR Pradaxa[tiab] OR Dabigatran[tiab] OR rivaroxaban[tiab] OR Xarelto[tiab] OR apixaban[tiab] OR edoxaban[tiab] OR ximelagatran[tiab] OR Exanta[tiab] OR melagatran[tiab] OR blood clotting factor 10a inhibitor[tiab] OR betrixaban[tiab] OR Coumarins[tiab] OR Warfarin[tiab] OR Eliquis[tiab] OR coumadin[tiab] OR acenocoumarol[tiab] OR phenprocumon[tiab] OR oral anticoagulant[tiab] OR vitamin K antagonist[tiab] OR VKA[tiab] OR Bivalirudin[tiab] OR Angiomax[tiab] | 156558 |
| **#3** | Perioperative Period[MeSH] OR Surgical Procedures, Operative[MeSH] OR Perioperative Period[tiab] OR Surgical Procedures, Operative[tiab] OR neurosurgical[tiab] OR resection[tiab] OR Colectomies[tiab] OR surgical[tiab] OR surgery[tiab] OR postoperative[tiab] OR operation[tiab] OR removal[tiab] OR perioperative[tiab] OR Postoperative Period[tiab] OR Colectomy[tiab] OR Proctectomy[tiab] OR Surgical Oncology[tiab] OR Gastrectomy[tiab] OR Denervation[tiab] OR Endoscopic Mucosal Resection[tiab] OR prostatectomy[tiab] OR craniotomy[tiab] OR Neurosurgical Procedures[tiab] OR polypectomy[tiab] OR postpolypectomy[tiab] OR endoscopic submucosal dissection[tiab] | 5963062 |
| **#4** | (Prospective Studies[MeSH] OR Observational Study [Publication Type] OR Observational Studies as Topic[MeSH] OR Case-Control Studies[MeSH] OR Retrospective Studies[MeSH] OR randomized controlled trial[Publication Type] OR controlled clinical trial[Publication Type] OR randomized[tiab] OR placebo[tiab] OR randomly[tiab] OR trial[tiab] OR Clinical Trials[tiab] OR Prospective Studies[tiab] OR Prospective Study[tiab] OR prospective[tiab] OR Case-Control Studies[tiab] OR case control study[tiab] OR Cohort Studies[tiab] OR cohort study[tiab] OR cohort analy*[tiab] OR Retrospective Studies[tiab] OR retrospective[tiab] OR observational study[tiab] OR Follow up study[tiab] OR Follow up studies[tiab] OR longitudinal[tiab] OR observational studies[tiab] OR case control) NOT (Animals[Mesh] NOT Humans[Mesh]) | 4549352 |
| **#5** | #1 AND #2 AND #3 AND #4 | 1643 |
| **Web of science (January 12, 2024)** | | |
| **#1** | (((((((((((((((((((((((((((((((((((((((((((((((TS=(carcinoma)) OR TS=(sarcoma) OR TS=(Neoplasms)) OR TS=(Neoplasm)) OR TS=(Tumor)) OR TS=(Neoplasia)) OR TS=(Cancer)) OR TS=(Malignancy)) OR TS=(Malignancies)) OR TS=(Benign Neoplasm)) OR TS=(oncomap)) OR TS=(ade-nocarcinoma)) OR TS=(tumour)) OR TS=(oncology)) OR TS=(arcin*)) OR TS=(cancer*)) OR TS=(neoplas*)) OR TS=(tumour*)) OR TS=(tumor*)) OR TS=(cyst*)) OR TS=(growth*)) OR TS=(adenocarcin*)) OR TS=(malignant)) OR TS=(malig*)) OR TS=(sarcoma)) OR TS=(melanoma)) OR TS=(osteosarcoma)) OR TS=(mesothelioma)) OR TS=(glioblastoma)) OR TS=(glioblastomas)) OR TS=(glioma)) OR TS=(Gliomas)) OR TS=(melanoma)) OR TS=(osteosarcoma)) OR TS=(hepatoblastoma)) OR TS=(Lymphoma)) OR TS=(chondrosarcoma)) OR TS=(meningioma)) OR TS=(choriocarcinoma)) OR TS=(fibrosarcoma)) OR TS=(liposarcoma)) OR TS=(Hemangiosarcoma)) OR TS=(Angiosarcoma)) OR TS=(mesothelioma)) OR TS=(leiomyosarcoma)) OR TS=(rhabdomyosarcoma)) OR TS=(cystadenocarcinoma)) OR TS=(seminoma)) | 12703455 |
| **#2** | (((((((((((((((((((((((((((((((((((((((((((((((((((((((((((((TS=(dalteparin)) OR TS=(Tedelparin)) OR TS=(Dalteparin Sodium)) OR TS=(Fragmin)) OR TS=(Fragmine)) OR TS=(Low molecular weight heparin)) OR TS=(LMWH)) OR TS=(Heparin)) OR TS=(Unfractionated Heparin)) OR TS=(Heparinic Acid)) OR TS=(Liquaemin)) OR TS=(Sodium Heparin)) OR TS=(Heparin Sodium)) OR TS=(alpha Heparinheparin )) OR TS=(Oral Factor Xa Inhibitor)) OR TS=(Fondaparinux)) OR TS=(Fondaparinux Sodium)) OR TS=(Quixidar)) OR TS=(Arixtra)) OR TS=(Arixtra)) OR TS=(Nadroparin)) OR TS=(Nadroparine)) OR TS=(Nadroparin Calcium)) OR TS=(Fraxiparine OR Enoxaparin)) OR TS=(fraxiparin)) OR TS=(clexane)) OR TS=(lovenox )) OR TS=(fragmin)) OR TS=(ardeparin)) OR TS=(normiﬂo)) OR TS=(tinzaparin)) OR TS=(logiparin)) OR TS=(innohep)) OR TS=(certoparin)) OR TS=(sandoparin)) OR TS=(reviparin)) OR TS=(clivarin)) OR TS=(danaproid)) OR TS=(orgaran)) OR TS=(Pradaxa)) OR TS=(Dabigatran)) OR TS=(rivaroxaban)) OR TS=(Xarelto)) OR TS=(apixaban)) OR TS=(edoxaban)) OR TS=(ximelagatran)) OR TS=(Exanta)) OR TS=(melagatran)) OR TS=(blood clotting factor 10a inhibitor)) OR TS=(betrixaban)) OR TS=(Coumarins)) OR TS=(Warfarin)) OR TS=(Eliquis)) OR TS=(coumadin)) OR TS=(acenocoumarol)) OR TS=(phenprocumon)) OR TS=(oral anticoagulant)) OR TS=(vitamin K antagonist)) OR TS=(VKA)) OR TS=(Bivalirudin)) OR TS=(Angiomax)) | 269598 |
| **#3** | ((((((((((((((((((((((((((TS=(Perioperative Period)) OR TS=(Surgical Procedures, Operative)) OR TS=(Perioperative Period)) OR TS=(Surgical Procedures, Operative)) OR TS=(neurosurgical)) OR TS=(resection)) OR TS=(Colectomies)) OR TS=(surgical)) OR TS=(surgery)) OR TS=(postoperative)) OR TS=(operation)) OR TS=(removal)) OR TS=(perioperative)) OR TS=(Postoperative Period)) OR TS=(Colectomy)) OR TS=(Proctectomy)) OR TS=(Surgical Oncology)) OR TS=(Gastrectomy)) OR TS=(Denervation)) OR TS=(Endoscopic Mucosal Resection)) OR TS=(prostatectomy)) OR TS=(craniotomy)) OR TS=(Neurosurgical Procedures)) OR TS=(polypectomy)) OR TS=(postpolypectomy)) OR TS=(endoscopic submucosal dissection)) | 7163555 |
| **#4** | (((((((((((((((((((((((TS=(randomized controlled trial)) OR TS=(controlled clinical trial)) OR TS=(randomized)) OR TS=(placebo)) OR TS=(randomly)) OR TS=(trial)) OR TS=(Clinical Trials)) OR TS=(Prospective Studies)) OR TS=(Prospective Study)) OR TS=(prospective)) OR TS=(Case-Control Studies)) OR TS=(case control study)) OR TS=(Cohort Studies)) OR TS=(cohort study)) OR TS=( cohort analy*)) OR TS=(Retrospective Studies)) OR TS=(retrospective)) OR TS=(observational study)) OR TS=(Follow up study)) OR TS=(Follow up studies)) OR TS=(longitudinal)) OR TS=(observational studies)) OR TS=(case control)) | 8710401 |
| **#5** | #1 AND #2 AND #3 AND #4 | 3988 |
| **Embase (January 12, 2024)** | | |
| **#1** | 'neoplasms'/exp | 6066872 |
| **#2** | 'Neoplasms':ab,kw,ti OR 'Neoplasm':ab,kw,ti OR 'Tumor':ab,kw,ti OR 'Neoplasm':ab,kw,ti OR 'Neoplasia':ab,kw,ti OR 'Cancer':ab,kw,ti OR 'Malignant':ab,kw,ti OR 'Malignancy':ab,kw,ti OR 'Malignancies':ab,kw,ti OR 'Benign Neoplasm':ab,kw,ti OR 'oncoma':ab,kw,ti OR 'carcinoma':ab,kw,ti OR 'ade-nocarcinoma':ab,kw,ti OR 'tumour':ab,kw,ti OR 'oncology':ab,kw,ti OR 'arcin*':ab,kw,ti OR 'cancer*':ab,kw,ti OR 'neoplas*':ab,kw,ti OR 'tumour*':ab,kw,ti OR 'tumor*':ab,kw,ti OR 'cyst*':ab,kw,ti OR 'growth*':ab,kw,ti OR 'adenocarcin*':ab,kw,ti OR 'malignant':ab,kw,ti OR 'malig*':ab,kw,ti OR 'sarcoma':ab,kw,ti OR 'glioblastoma':ab,kw,ti OR 'glioblastomas':ab,kw,ti OR 'glioma':ab,kw,ti OR 'Gliomas':ab,kw,ti OR 'melanoma':ab,kw,ti OR 'osteosarcoma':ab,kw,ti OR 'hepatoblastoma':ab,kw,ti OR 'Lymphoma':ab,kw,ti OR 'chondrosarcoma':ab,kw,ti OR 'meningioma':ab,kw,ti OR 'choriocarcinoma':ab,kw,ti OR 'fibrosarcoma':ab,kw,ti OR 'liposarcoma':ab,kw,ti OR 'Hemangiosarcoma':ab,kw,ti OR 'Angiosarcoma':ab,kw,ti OR 'mesothelioma':ab,kw,ti OR 'leiomyosarcoma':ab,kw,ti OR 'rhabdoyosarcoma':ab,kw,ti OR 'cystadenocarcinoma':ab,kw,ti OR 'seminoma':ab,kw,ti | 7706804 |
| **#3** | #1 OR #2 | 9001709 |
| **#4** | 'apixaban'/exp | 20712 |
| **#5** | 'rivaroxaban'/exp | 27590 |
| **#6** | 'dalteparin':ab,kw,ti OR 'Tedelparin':ab,kw,ti OR 'Dalteparin Sodium':ab,kw,ti OR 'Fragmin':ab,kw,ti OR 'Fragmine':ab,kw,ti OR 'Low molecular weight heparin':ab,kw,ti OR 'LMWH':ab,kw,ti OR 'Heparin':ab,kw,ti OR 'Unfractionated Heparin':ab,kw,ti OR 'Heparinic Acid':ab,kw,ti OR 'Liquaemin':ab,kw,ti OR 'Sodium Heparin':ab,kw,ti OR 'Heparin Sodium':ab,kw,ti OR 'alpha Heparinheparin ':ab,kw,ti OR 'Oral Factor Xa Inhibitor':ab,kw,ti OR 'Fondaparinux':ab,kw,ti OR 'Fondaparinux Sodium':ab,kw,ti OR 'Quixidar':ab,kw,ti OR 'Arixtra':ab,kw,ti OR 'Arixtra':ab,kw,ti OR 'Nadroparin':ab,kw,ti OR ' Nadroparine':ab,kw,ti OR ' Nadroparin Calcium':ab,kw,ti OR ' Fraxiparine':ab,kw,ti OR Enoxaparin':ab,kw,ti OR 'fraxiparin':ab,kw,ti OR 'clexane':ab,kw,ti OR 'lovenox':ab,kw,ti OR 'fragmin':ab,kw,ti OR 'ardeparin':ab,kw,ti OR ' normiﬂo':ab,kw,ti OR 'tinzaparin':ab,kw,ti OR 'logiparin':ab,kw,ti OR 'innohep':ab,kw,ti OR 'certoparin':ab,kw,ti OR 'sandoparin':ab,kw,ti OR 'reviparin':ab,kw,ti OR 'clivarin':ab,kw,ti OR 'danaproid':ab,kw,ti OR 'orgaran':ab,kw,ti OR 'Pradaxa':ab,kw,ti OR 'Dabigatran':ab,kw,ti OR 'rivaroxaban':ab,kw,ti OR 'Xarelto':ab,kw,ti OR 'apixaban':ab,kw,ti OR 'edoxaban':ab,kw,ti OR 'ximelagatran':ab,kw,ti OR 'Exanta':ab,kw,ti OR 'melagatran':ab,kw,ti OR 'blood clotting factor 10a inhibitor':ab,kw,ti OR 'betrixaban':ab,kw,ti OR 'Coumarins':ab,kw,ti OR 'Warfarin':ab,kw,ti OR 'Eliquis':ab,kw,ti OR 'coumadin':ab,kw,ti OR 'acenocoumarol':ab,kw,ti OR 'phenprocumon':ab,kw,ti OR 'oral anticoagulant':ab,kw,ti OR 'vitamin K antagonist':ab,kw,ti OR 'VKA':ab,kw,ti OR 'Bivalirudin':ab,kw,ti OR 'Angiomax':ab,kw,ti | 197613 |
| **#7** | #4 OR #5 OR #6 | 209710 |
| **#8** | 'surgery'/exp | 6168481 |
| **#9** | 'Perioperative Period':ab,kw,ti OR 'Surgical Procedures, Operative':ab,kw,ti OR 'neurosurgical':ab,kw,ti OR 'resection':ab,kw,ti OR 'Colectomies':ab,kw,ti OR 'surgical':ab,kw,ti OR 'surgery':ab,kw,ti OR 'postoperative':ab,kw,ti OR 'operation':ab,kw,ti OR 'removal':ab,kw,ti OR 'perioperative':ab,kw,ti OR 'Postoperative Period':ab,kw,ti OR 'Colectomy':ab,kw,ti OR 'Proctectomy':ab,kw,ti OR 'Surgical Oncology':ab,kw,ti OR 'Gastrectomy':ab,kw,ti OR 'Denervation':ab,kw,ti OR 'Endoscopic Mucosal Resection':ab,kw,ti OR 'prostatectomy':ab,kw,ti OR 'craniotomy':ab,kw,ti OR 'Neurosurgical Procedures':ab,kw,ti OR 'polypectomy':ab,kw,ti OR 'postpolypectomy':ab,kw,ti OR 'endoscopic submucosal dissection':ab,kw,ti | 4254424 |
| **#10** | #8 OR #9 | 7537997 |
| **#11** | 'randomized controlled trial'/exp | 789807 |
| **#12** | 'randomized controlled trial':ab,kw,ti OR 'controlled clinical trial':ab,kw,ti OR 'randomized':ab,kw,ti OR 'placebo':ab,kw,ti OR 'randomly':ab,kw,ti OR 'trial':ab,kw,ti OR 'Clinical Trials':ab,kw,ti OR 'Prospective Studies':ab,kw,ti OR 'Prospective Study':ab,kw,ti OR 'prospective':ab,kw,ti OR 'Case-Control Studies':ab,kw,ti OR 'case control study':ab,kw,ti OR 'Cohort Studies':ab,kw,ti OR 'cohort study':ab,kw,ti OR ' cohort analy*':ab,kw,ti OR 'Retrospective Studies':ab,kw,ti OR 'retrospective':ab,kw,ti OR 'observational study':ab,kw,ti OR 'Follow up study':ab,kw,ti OR 'Follow up studies':ab,kw,ti OR 'longitudinal':ab,kw,ti OR 'observational studies':ab,kw,ti OR 'case control':ab,kw,ti | 5138471 |
| **#13** | #11 OR #12 | 5246499 |
| **#18** | #3 AND #7 AND #10 AND #13 | 3375 |
| **Cochranne (January 12, 2024)** | | |
| **#1** | MeSH descriptor: [Neoplasms] explode all trees | 114562 |
| **#2** | ("Neoplasms" OR "Neoplasm" OR "Tumor" OR "Neoplasm" OR "Neoplasia" OR "Cancer" OR "Malignant" OR "Malignancy" OR "Malignancies" OR "Benign Neoplasm" OR "oncoma" OR "carcinoma" OR "ade-nocarcinoma" OR "tumour" OR "oncology" OR "malignant" OR "sarcoma" OR "glioblastoma" OR "glioblastomas" OR "glioma" OR "Gliomas" OR "melanoma" OR "osteosarcoma" OR "hepatoblastoma" OR "Lymphoma" OR "chondrosarcoma" OR "meningioma" OR "choriocarcinoma" OR "fibrosarcoma" OR "liposarcoma" OR "Hemangiosarcoma" OR "Angiosarcoma" OR "mesothelioma" OR "leiomyosarcoma" OR "rhabdoyosarcoma" OR "cystadenocarcinoma" OR "seminoma"):ab,kw,ti | 273422 |
| **#3** | #1 OR #2 | 284257 |
| **#4** | MeSH descriptor: [Rivaroxaban] explode all trees | 819 |
| **#5** | MeSH descriptor: [Heparin, Low-Molecular-Weight] explode all trees | 2421 |
| **#6** | MeSH descriptor: [Heparin] explode all trees | 5633 |
| **#7** | (“dalteparin" OR "Tedelparin" OR "Dalteparin Sodium" OR "Fragmin" OR "Fragmine" OR "Low molecular weight heparin" OR "LMWH" OR "Heparin" OR "Unfractionated Heparin" OR "Heparinic Acid" OR "Liquaemin" OR "Sodium Heparin" OR "Heparin Sodium" OR "alpha Heparinheparin " OR "Oral Factor Xa Inhibitor" OR "Fondaparinux" OR "Fondaparinux Sodium" OR "Quixidar" OR "Arixtra" OR "Arixtra" OR "Nadroparin" OR " Nadroparine" OR " Nadroparin Calcium" OR " Fraxiparine OR Enoxaparin" OR "fraxiparin" OR "clexane" OR "lovenox " OR "fragmin" OR "ardeparin" OR " normiﬂo" OR "tinzaparin" OR "logiparin" OR "innohep" OR "certoparin" OR "sandoparin" OR "reviparin" OR "clivarin" OR "danaproid" OR "orgaran" OR "Pradaxa" OR "Dabigatran" OR "rivaroxaban" OR "Xarelto" OR " apixaban" OR "edoxaban" OR "ximelagatran" OR "Exanta" OR "melagatran" OR "blood clotting factor 10a inhibitor" OR "betrixaban" OR "Coumarins" OR "Warfarin" OR "Eliquis" OR "coumadin" OR "acenocoumarol" OR "phenprocumon" OR "oral anticoagulant" OR "vitamin K antagonist" OR "VKA" OR "Bivalirudin" OR "Angiomax”):ab,kw,ti | 21670 |
| **#8** | #5 OR #6 OR #7 OR #4 | 21889 |
| **#9** | MeSH descriptor: [General Surgery] explode all trees | 502 |
| **#10** | ('Perioperative Period" OR "Surgical Procedures, Operative" OR "neurosurgical" OR "resection" OR "Colectomies" OR "surgical" OR "surgery" OR "postoperative" OR "operation" OR "removal" OR "perioperative" OR "Postoperative Period" OR "Colectomy" OR "Proctectomy" OR "Surgical Oncology" OR "Gastrectomy" OR "Denervation" OR "Endoscopic Mucosal Resection" OR "prostatectomy" OR "craniotomy" OR "Neurosurgical Procedures" OR "polypectomy" OR "postpolypectomy" OR "endoscopic submucosal dissection'):ab,kw,ti | 1253318 |
| **#11** | #9 OR #10 | 1253319 |
| **#12** | MeSH descriptor: [Randomized Controlled Trial] explode all trees | 25734 |
| **#13** | MeSH descriptor: [Controlled Clinical Trial] explode all trees | 38479 |
| **#14** | MeSH descriptor: [Random Allocation] explode all trees | 24166 |
| **#15** | MeSH descriptor: [Placebos] explode all trees | 26240 |
| **#16** | (((((((((((((((((((((((TS=(randomized controlled trial)) OR TS=(controlled clinical trial)) OR TS=(randomized)) OR TS=(placebo)) OR TS=(randomly)) OR TS=(trial)) OR TS=(Clinical Trials)) OR TS=(Prospective Studies)) OR TS=(Prospective Study)) OR TS=(prospective)) OR TS=(Case-Control Studies)) OR TS=(case control study)) OR TS=(Cohort Studies)) OR TS=(cohort study)) OR TS=( cohort analy*)) OR TS=(Retrospective Studies)) OR TS=(retrospective)) OR TS=(observational study)) OR TS=(Follow up study)) OR TS=(Follow up studies)) OR TS=(longitudinal)) OR TS=(observational studies)) OR TS=(case contro | 1803171 |
| **#17** | #12 OR #13 OR #14 OR #15 OR #16 | 1807136 |
| **#18** | #3 AND #8 AND #11 AND #17 | 1611 |

**Supplementary Table 3.** Outcome of included studies

| **Author** | **Year** | **Intervention type** | **Total (n)** | **Venous thromboembolism** | | | **Major bleeding** | **Bleeding** | **Adverse events** | **Outcomes** | **The outcome time** |
| --- | --- | --- | --- | --- | --- | --- | --- | --- | --- | --- | --- |
|  |  |  |  | **PE** | **DVT** | **Total** |  |  |  |  |  |
| **Clarke-pearson [17]** | 1982 | UFH | 88 | 4 | 3 | 13 | NR | NR | NR | a,b | 42 |
|  |  | No treatment | 97 | 1 | 1 | 12 | NR | NR | NR |  | 42 |
| **Clarke-Pearson [18]** | 1984 | Physiotherapy | 97 | 4 | 14 | 18 | NR | NR | NR | a,b | 42 |
|  |  | No treatment | 97 | 1 | 11 | 12 | NR | NR | NR |  | 42 |
| **Clarke-Pearson [19]** | 1984 | UFH | 88 | NR | NR | NR | NR | 2 | 5 | c,d | NR |
|  |  | No treatment | 94 | NR | NR | NR | NR | 1 | 3 |  | NR |
| **Fricker [20]** | 1988 | LMWH | 40 | 0 | 0 | 0 | 4 | 10 | 4 | a, b, c, d | 28 |
|  |  | UFH | 40 | 2 | 0 | 2 | 12 | 21 | 1 |  | 28 |
| **Clarke-Pearson [21]** | 1990 | UFH | 201 | 3 | 13 | 16 | NR | 8 | 40 | a, b, c, d | 7 |
|  |  | No treatment | 103 | 0 | 19 | 19 | NR | 0 | 16 |  | 7 |
| **Clarke-Pearson [22]** | 1993 | UFH | 107 | 0 | 7 | 7 | 3 | 3 | 31 | a, c, d | 30 |
|  |  | Physiotherapy | 101 | 0 | 4 | 4 | 0 | 0 | 29 |  | 30 |
| **Maxwell [23]** | 2001 | LMWH | 105 | 0 | 2 | 2 | 4 | 5 | 6 | a, b, c, d | 3~5 |
|  |  | Physiotherapy | 106 | 0 | 1 | 1 | 3 | 3 | 5 |  | 3~5 |
| **Bergqvist [24]** | 2002 | LMWH | 165 | 0 | 8 | 8 | 3 | 18 | 10 | a, b, c, d | 25-31 |
|  |  | Placebo | 167 | 1 | 20 | 21 | 1 | 11 | 8 |  | 25-31 |
| **Sakon [25]** | 2010 | LMWH | 83 | 0 | 1 | 1 | 5 | 10 | 103 | a, b, c, d | 14 |
|  |  | Physiotherapy | 31 | 0 | 6 | 6 | 1 | 3 | 37 |  | 14 |
| **Kakkar [26]** | 2010 | LMWH | 315 | 0 | 19 | 19 | 2 | 4 | 1 | a, b, c, d | 18~22 |
|  |  | Placebo | 310 | 0 | 29 | 29 | 1 | 2 | 0 |  | 18~22 |
| **Song [27]** | 2014 | LMWH＋Physiotherapy | 108 | 0 | 3 | 3 | 2 | 11 | 17 | a, c, d | 30 |
|  |  | Physiotherapy | 112 | 0 | 3 | 3 | 0 | 1 | 19 |  | 30 |
| **Nagata [28]** | 2015 | LMWH | 16 | 0 | 1 | 1 | NR | NR | NR | a | 9~11 |
|  |  | Physiotherapy | 14 | 3 | 3 | 5 | NR | NR | NR |  | 9~11 |
| **Pootracool [29]** | 2017 | LMWH＋Physiotherapy | 54 | 0 | 1 | 1 | 1 | 2 | NR | a, c | 35 |
|  |  | No treatment | 54 | 0 | 2 | 2 | 0 | 0 | NR |  | 35 |
| **Hata [30]** | 2019 | LMWH＋Physiotherapy | 145 | 1 | 3 | 4 | 2 | 19 | NR | a, b, c | 7~16 |
|  |  | Physiotherapy | 157 | 3 | 5 | 8 | 2 | 5 | NR |  | 7~16 |
| **Nakagawa [31]** | 2020 | LMWH＋Physiotherapy | 57 | 0 | 0 | 7 | 0 | 1 | 9 | a, c, d | 28 |
|  |  | Physiotherapy | 59 | 0 | 0 | 7 | 0 | 0 | 7 |  | 28 |
| **Guntupalli [14]** | 2020 | DOACs＋Physiotherapy | 204 | NR | NR | 2 | 1 | 13 | 41 | a, b, c, d | 30 |
|  |  | LMWH＋Physiotherapy | 196 | NR | NR | 3 | 1 | 20 | 26 |  | 30 |
| **Zhou [32]** | 2020 | LMWH＋Physiotherapy | 46 | NR | 1 | 1 | NR | NR | NR | a | 7 |
|  |  | LMWH | 46 | NR | 9 | 9 | NR | NR | NR |  | 7 |
| **Kamach [33]** | 2020 | LMWH＋Physiotherapy | 182 | 1 | 5 | 6 | 1 | 11 | NR | a, b, c | 7 |
|  |  | Physiotherapy | 208 | 6 | 6 | 10 | 0 | 0 | NR |  | 7 |
| **Longo de Oliveira [13]** | 2022 | DOACs | 114 | 0 | 0 | 4 | 0 | 20 | NR | a, b, c | 30 |
|  |  | LMWH | 114 | 0 | 0 | 5 | 0 | 19 | NR |  | 30 |
| **Becattini [12]** | 2022 | DOACs | 287 | 3 | 0 | 3 | 2 | 5 | NR | a, b, c | 28±2 |
|  |  | Placebo | 282 | 7 | 4 | 11 | 0 | 5 | NR |  | 28±2 |

**Abbreviation:** UFH: unfractionated heparin; LMWH: low molecular weight heparin; DOACs: new-oral-anticoagulants; n: number of patients; NR:not reported;

PE: pulmonary embolism; DVT: deep venous thromboembolismolism; a: venous thromboembolismolism; b: major bleeding; c: bleeding; d: adverse events.

**Supplementary Table 4.** Characteristics of included studies

| **Author** | **Year** | **Total (n)** | **Country** | **Study design** | **Intervention** | **Intervention type** | **Dosage of each**  **administration** | **Total dosage**  **per day** | **Route** | **Duration**  **(days)** | **Age**  **(years)** | **BMI**  **(kg/m2 )** | **Race/ethnicity** | **Type of**  **cancer** | **Cancer stage (n)** | | **Benign tumour** | **Surgery**  **Time (h)** |
| --- | --- | --- | --- | --- | --- | --- | --- | --- | --- | --- | --- | --- | --- | --- | --- | --- | --- | --- |
|  |  |  |  |  |  |  |  |  |  |  |  |  |  |  | **I/II** | **I/IV** |  |  |
| **Clarke-pearson [17]** | 1983 | 88 | USA | RCT | Sodium heparin(lose) | UFH | 5000 IU | 10000 IU | SC | 7 | 70.3 | NR | White, Black | Gyne | 41 | 9 | 17 | NR |
|  |  | 97 |  |  | No treatment | No treatment | NR | NR | NR | NR | 69.5 | NR |  |  | 54 | 8 | 12 | NR |
| **Clarke-Pearson [18]** | 1984 | 97 | USA | RCT | SCD | Physiotherapy | NR | NR | NR | Discharge | NR | NR | White, Black | Gyne | NR | NR | 0 | NR |
|  |  | 97 |  |  | No treatment | No treatment | NR | NR | NR | NR | NR | NR |  |  | NR | NR | 0 | NR |
| **Clarke-Pearson [19]** | 1984 | 88 | USA | RCT | Sodium heparin(lose) | UFH | 5000 IU | 10000 IU | SC | 7 | 53 | NR | White, Black | Gyne | NR | NR | 0 | NR |
|  |  | 94 |  |  | No treatment | No treatment | NR | NR | NR | NR | NR | NR |  |  | NR | NR | 0 | NR |
| **Fricker [20]** | 1988 | 40 | France | RCT | Dalteparin | LMWH | 5000 antiXaIU | 5000 antiXaIU | SC | 10 | 58.2 | 24.5 | NR | GI/GU/Gyne | NR | NR | 0 | 3.8 |
|  |  | 40 |  |  | Calcium heparin | UFH | 5000 IU | 15000 IU | SC | 10 | 57 | 25.2 |  |  | NR | NR | 0 | 3.4 |
| **Clarke-Pearson [21]** | 1990 | 201 | USA | RCT | Calcium heparin | UFH | 5000 IU | 15000 IU | SC | 7 | 60.8 | NR | White, Black, Other | Gyne | 254 | | 50 | 3.2 |
|  |  | 103 |  |  | No treatment | No treatment | NR | NR | NR | NR | 60 | NR |  |  |  |  |  | 3.2 |
| **Clarke-Pearson [22]** | 1993 | 107 | USA | RCT | Heparin(lose) | UFH | 5000 IU | 15000 IU | SC | 7 | 57 | NR | White, Black | Gyne | 158 | | 50 | 3.8 |
|  |  | 101 |  |  | IPC | Physiotherapy | NR | NR | NR | 5 | 55 | NR |  |  |  |  |  | 3.9 |
| **Maxwell [23]** | 2001 | 105 | USA | RCT | Dalteparin | LMWH | 5000 antiXaIU | 5000 antiXaIU | SC | 5 | 60 | 28 | White | Gyne | 85 | | 20 | 3.3 |
|  |  | 106 |  |  | EPC | Physiotherapy | NR | NR | NR | 5 | 25 | 27.1 |  |  | 83 | | 23 | 3.3 |
| **Bergqvist [24]** | 2002 | 165 | Sweden,  Italy, et al | RCT | Enoxaparin | LMWH | 40mg | 40mg | SC | 19-21 | 66 | 25 | NR | GI/Gyne | NR | NR | 0 | 3.2 |
|  |  | 167 |  |  | Placebo | Placebo | 40mg | 40mg | SC | 19-21 | 65 | 25 |  |  | NR | NR | 0 | 3.1 |
| **Sakon [25]** | 2010 | 83 | Japan | RCT | Enoxaparin | LMWH | 20mg | 40mg | SC | 14 | 67.7 | 22.5 | Asian | GI/GU/Gyne | NR | NR | 0 | 3.5 |
|  |  | 31 |  |  | IPC | Physiotherapy | NR | NR | NR | 14 | 66.1 | 22.5 |  |  | NR | NR | 0 | 3.7 |
| **Kakkar [26]** | 2010 | 315 | Romania Spain Russia | RCT | Bemiparin | LMWH | 3500IU | 3500IU | SC | 20 | 64.1 | 26 | NR | GI/GU/Gyne | 184 | 131 | 0 | 2.6 |
|  |  | 310 |  |  | 0.9%NaCl | Placebo | 0.2ml | 0.2ml 0.9%NaCl | SC | 20 | 64.6 | 25.7 |  |  | 161 | 149 | 0 | 2.6 |
| **Song [27]** | 2014 | 108 | Korea | RCT | Enoxaparin＋IPC | LMWH＋Physiotherapy | 40mg | 40mg | SC | 7~10 | 56.31 | 23.76 | Asian | GI | NR | NR | 0 | NR |
|  |  | 112 |  |  | IPC | Physiotherapy | NR | NR | NR | 7~10 | 58.77 | 23.84 |  |  | NR | NR | 0 | NR |
| **Nagata [28]** | 2015 | 16 | Japan | RCT | Enoxaparin | LMWH | 2000 antiXaIU | 4000 antiXaIU | SC | 7 | 60.5 | 23.3 | Asian | Gyne | 10 | 5 | 0 | 4.2 |
|  |  | 14 |  |  | SCD | Physiotherapy | NR | NR | NR | Ambulation | 53.2 | 21.2 |  |  | 10 | 4 | 0 | 3.7 |
| **Pootracool [29]** | 2017 | 54 | Thailand | RCT | LMWH＋SCD | LMWH＋Physiotherapy | 0.4ml | 0.4m | SC | 28 | 60 | 27.4 | NR | GU/Gyne | 33 | 20 | 1 | 3.0 |
|  |  | 54 |  |  | No treatment | No treatment | NR | NR | NR | 28 | 60.9 | 24.9 |  |  | 28 | 20 | 6 | 2.6 |
| **Hata [30]** | 2019 | 145 | Japan | RCT | Enoxaparin or FPX＋IPC＋GCS | LMWH＋Physiotherapy | 20000IU | 40000IU | SC | 6.0 ± 2.05 | 65.4 | 22.6 | Asian | CRC | 85 | 52 | 0 | 4.7±5.2 |
|  |  | 157 |  |  | IPC＋GCS | Physiotherapy | NR | NR | NR | 1.19 ± 0.58 | 64.9 | 22.5 |  |  | 98 | 52 | 0 | 5.0±0.2 |
| **Nakagawa [31]** | 2020 | 57 | Japan | RCT | Enoxaparin＋IPC | LMWH＋Physiotherapy | 20mg | 40mg | SC | Discharge | 69.0 | 22.3 | Asian | CRC | 39 | 17 | 0 | 3.0 |
|  |  | 59 |  |  | IPC | Physiotherapy | NR | NR | NR | Discharge | 67.0 | 22.5 |  |  | 44 | 14 | 0 | 2.9 |
| **Guntupalli [14]** | 2020 | 204 | USA | RCT | Apixaban＋IPC | DOACs＋Physiotherapy | 2.5mg | 5mg | PO | 28 | 58.0 | NR | Hispanic African, American, Asian | Gyne | 80 | 75 | 40 | 3.6 |
|  |  | 196 |  |  | Enoxaparin＋IPC | LMWH＋Physiotherapy | 40mg | 40mg | SC | 28 | 58.5 | NR |  |  | 84 | 80 | 37 | 3.4 |
| **Zhou [32]** | 2020 | 46 | China | RCT | Enoxaparin＋IPC＋ES | LMWH＋Physiotherapy | 3000IU | 3000IU | SC | 7 | 46.62±5.55 | NR | Asian | Gyne | 8 | 38 | 0 | 3.12±0.95 |
|  |  | 46 |  |  | Enoxaparin | LMWH | 3000IU | 3000IU | SC | 7 | 46.52±5.12 | NR |  |  | 7 | 39 | 0 | 3.14±0.83 |
| **Kamach [33]** | 2020 | 182 | Japan | RCT | Enoxaparin+IPC | LMWH＋Physiotherapy | 2000IU | 4000IU | SC | 7 | 65 | 23.7 | Asian | GI | 136 | 45 | 0 | 3.8 |
|  |  | 208 |  |  | IPC | Physiotherapy | NR | NR | NR | 7 | 64.8 | 23.3 |  |  | 150 | 55 | 0 | 3.7 |
| **Longo de Oliveira [13]** | 2022 | 114 | Brazil | RCT | Rivaroxaban | DOACs | 10mg | 10mg | PO | 30 | 54 | 28.5 | NR | Gyne | 75 | 20 | 5 | 2.4 |
|  |  | 114 |  |  | Enoxaparin | LMWH | 40mg | 40mg | SC | 30 | 56 | 28.1 |  |  | 67 | 35 | 2 | 2.3 |
| **Becattini [12]** | 2022 | 287 | Italian | RCT | Rivaroxaban | DOACs | 10mg | 10mg | PO | 21 | 65.8 ± 11.3 | NR | NR | CRC | 125 | 162 | 0 | 3.1 |
|  |  | 282 |  |  | Placebo | Placebo | NR | NR | NR | 21 | 64.5 ± 11.1 | NR |  |  | 114 | 168 | 0 | 3.1 |

Study-level characteristics that were judged to threaten transitivity and that formed the basis for exclusion, are bold and underscored.

**Abbreviation:** GCS: graduated compression stockings; ES: elastic stockings; IPC: intermittent pneumatic; SCD: sequential compression device; EPS:external pneumatic compression; UFH: unfractionated heparin; FPX:Fondaparinux, LMWH: low molecular weight heparin; DOACs:new-oral-anticoagulants; NR: not reported; antiXaIU: antifactor Xa international unit; IU: international unit; SC: Subcutaneous injection; PO: per os; BMI: body mass index; n: number of patients.
